# Supplementary material for: High throughput screening of novel AAV capsids identifies variants for transduction of adult NSCs within the subventricular zone
Source: Mol Ther Methods Clin Dev. 2021 Jul 16;23:33–50. doi: 10.1016/j.omtm.2021.07.001 (PMC8427210; doi:10.1016/j.omtm.2021.07.001)
Supplement: Document S1. Data S1, Tables S1−S6; and Figures S1–S6 — ▪▪▪ [file mmc1.pdf]

OMTM, Volume *23*

Supplemental information

High throughput screening of novel AAV capsids identifies variants for transduction of adult NSCs within the subventricular zone

Lukas P.M. Kremer, Santiago Cerrizuela, Sascha Dehler, Thomas Stiehl, Jonas Weinmann, Heike Abendroth, Susanne Kleber, Alexander Laure, Jihad El Andari, Simon Anders, Anna Marciniak-Czochra, Dirk Grimm, and Ana Martin-Villalba

Data S1. Mathematical Modeling of Labeling Dynamics with AAV1 P5 transduced v-SVZ cells

Mathematical Modeling of Labeling Dynamics with AAV1_P5 transduced v-SVZ cells

1 Mathematical Model of Neurogenesis

We extend our previously established mathematical model from [1–3]. The model describes time evolution of active NSC, quiescent NSC and TAPs. The model considers the following processes:

- Quiescent stem cells are activated at the rate r. As demonstrated in [1] the activation rate depends on the age of the organism.
- Division of active stem cells occurs at the rate p_{stem}. Upon division a stem cell gives rise to two progeny.
- The probability that a progeny is a stem cell is b. It is referred to as self-renewal probability. With probability $(1-b)$ the progeny differentiates into a TAP.
- TAPs divide a finite number of times before they further differentiate.

The part of the model describing stem cell dynamics has been parameterized in [1]. The TAP dynamics have not been calibrated so far. We assume a TAP doubling time of 20.15 hours taken from [4]. Assuming 4 TAP divisions we obtain the best agreement of model simulations with the TAP data from [1], see Figure 1. This number of TAP divisions is in agreement with the measurements from [4]. We then obtain the following model.

$$
\begin{aligned}
\frac{d}{dt}qNSC =& - r(t) \cdot qNSC + 2 \cdot b \cdot p_{stem} aNSC \\
\frac{d}{dt}aNSC =& r(t) \cdot qNSC - p_{stem} \cdot aNSC \\
\frac{d}{dt}TAP_0 =& - p_{prog} \cdot TAP_0 + 2 \cdot (1 - b) \cdot p_{stem} \cdot aNSC \\
\frac{d}{dt}TAP_1 =& - p_{TAP} \cdot TAP_1 + 2 \cdot p_{TAP} \cdot TAP_0 \\
\frac{d}{dt}TAP_2 =& - p_{TAP} \cdot TAP_2 + 2 \cdot p_{TAP} \cdot TAP_1 \\
\frac{d}{dt}TAP_3 =& - p_{TAP} \cdot TAP_3 + 2 \cdot p_{TAP} \cdot TAP_2 \\
r(t) =& r_{max} \exp(-\beta_r t)
\end{aligned}
\tag{1}
$$

As $qNSC(t)$ and $aNSC(t)$ we denote the amount of quiescent and active neural stem cells at time t. As $TAP_i(t)$, $i \in \{0,\ 1,\ 2,\ 3\}$ we denote the amount of TAPs that have performed i divisions at time t. Namely, $aNSC$ give rise to TAP_0. If TAP_0 divide, the progeny belong to TAP_1, i.e., TAP that have performed one division. Analogously TAP_1 give rise to TAP_2, TAP_2 give rise to TAP_3 and progeny of TAP_3 are neuroblasts. For notational convenience we omit the argument t and identify $qNSC(t) \equiv qNSC$, $aNSC(t) \equiv aNSC$, $TAP_i(t) \equiv TAP_i$. Proliferation rates of stem cells and TAP are denoted as p_{stem} and p_{TAP} respectively. By b we denote the probability of stem cell self-renewal. It is the probability with which a progeny of a stem cell is again a stem cell [5–7]. We note that the initial condition for TAPs has practically no impact on the cell counts at ages larger than 1 month. All model parameters are summarized in Table 1.

2 Modeling of Labeling Dynamics

The serotype is injected in mice of age $\tau = 56$ days. We denote the time of serotype injection as $t = 0$.

We make the following assumptions

- The transduced cell expresses a fluorescent label that is transmitted over the cell division. We assume that proliferation rates and self-renewal probability are not affected by the virus.

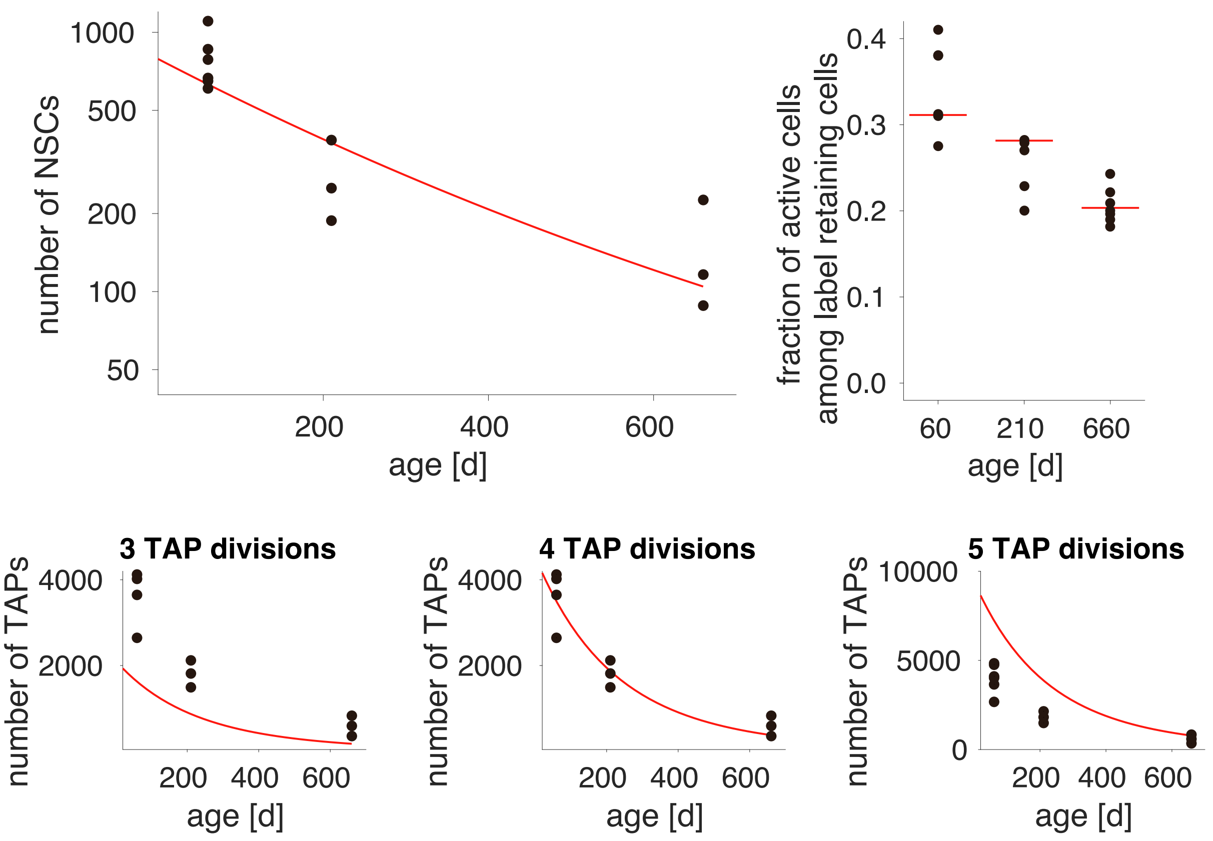

Figure 1: Simulation of NSC and TAP dynamics during aging. Upper: NSC dynamics. Lower: TAP dynamics assuming 3, 4 or 5 TAP divisions before further differentiation. We obtain the best agreement of model and data for 4 TAP divisions. Red curve: Model simulations, black dots: data from [1]. The figures are obtained from simulation of model (1) with the parameters specified in Table 1.

parameter	value
r_{max}	$0.453\ d^{-1}$
β_r	$9.5 \cdot 10^{-4}\ d^{-1}$
b	0.494
p_{stem}	$0.951\ d^{-1}$
p_{TAP}	$0.826\ d^{-1}$
$qNSC(0)$	535
aNSC(0)	255

Table 1: Parameters of the neurogenesis model. Parameters are taken from [1].

- The probability for a single TAP to be labeled is the same for TAP_0, TAP_1, TAP_2, and TAP_3.
- After the fourth division a TAP gives rise to two neuroblasts.
- Neuroblasts arrive after a delay θ in the olfactory bulb. During migration neuroblasts can divide and die. For this reason we assume that the amount of neuroblasts arriving in the olfactory bulb at time t is $\mu \cdot NB(t-\theta)$. If proliferation outweights death $\mu > 1$, otherwise $\mu < 1$. The factor μ also takes into account the different volumes of SVZ and OB.
- We neglect the death of labeled OB cells during the duration of the experiment.

Dynamics of labeled cells is given by the system of equations (1) supplemented by the following equation for olfactory bulb neurons

$$\frac{d}{dt}OB = \mu \cdot p_{TAP} \cdot TAP_3(t-\theta) \tag{2}$$

and the initial condition

$$\begin{aligned} qNSC(0) &= qNSC_0 \\ aNSC(0) &= aNSC_0 \\ TAP_i(0) &= TAP_{i,0},\ i \in \{0,\ 1,\ 2,\ 3\} \\ OB(0) &= 0. \end{aligned} \tag{3}$$

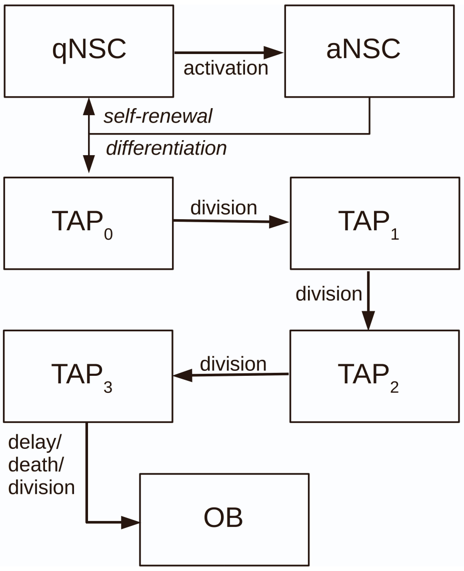

Figure 2: Model of serotype labeling. The scheme depicts the processes described by system (1)-(4). $qNSC$: labeled quiescent NSC, $aNSC$: labeled active NSC, TAP_i: labeled TAPs that have performed i divisions, OB: labeled cells in the olfactory bulb.

The age of the mice at the beginning of the experiment is $\tau = 56d$. Since we define the time when the experiment starts as $t = 0$, the equation for r is given by

$$r(t) = r_{max} \exp(-\beta_r (t + \tau)). \tag{4}$$

The model is visualized in Figure 2. In agreement with the quasi-steady state cell counts we set $TAP_{i,0} = 2 \cdot TAP_{i-1,0}$ for $i \in \{1,\ 2,\ 3\}$. For $t < 0$ all populations equal 0.

3 Data

We consider densities of labeled NSC (given per mm^2 of SVZ) and labeled olfactory bulb neurons (given per mm^3 of olfactory bulb). The data shows high heterogeneity among individual mice, with more than an order of magnitude between individual measurements.

Instead of fitting the model to average cell counts we subdivide the data into two groups. The data and the subgroups are shown in Figure 3. We ask whether we can fit the data of

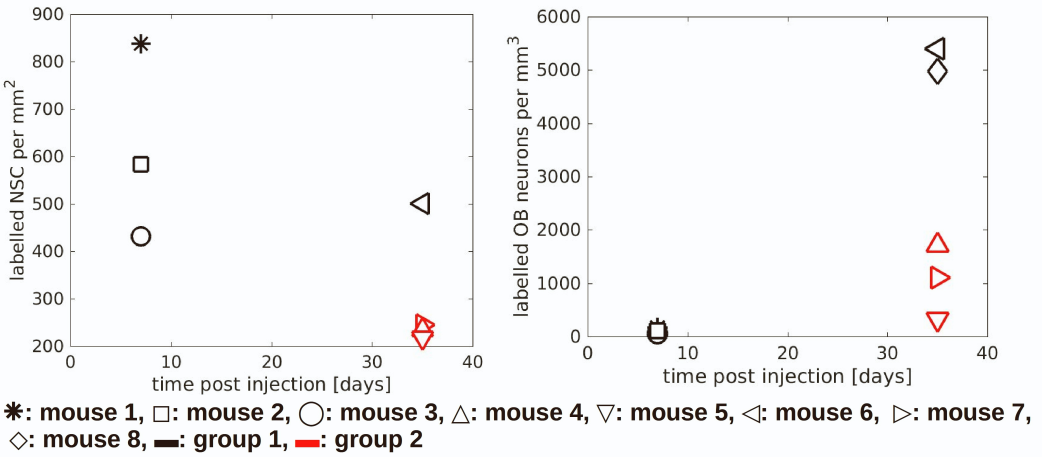

Figure 3: Time dynamics of labeled cells. Each mouse is identified by one symbol. Due to the heterogeneity among individual mice, each mouse was assigned to one of two groups. The color of the symbols indicates to which group the respective mouse belongs.

both groups assuming that they differ only with respect to the number of initially labeled NSC and TAP.

Taking into account the heterogeneity among mice the data was assigned to two different groups as follows:

- The data at day 35 was subdivided into one group of mice showing high numbers of labeled cells (group 1) and one group of mice showing low numbers of labeled cells (group 2).
- The mice studied at day 7 show less heterogeneity, therefore they are not subdivided.
- Taking into account that stem cell numbers practically do not vary in a time interval of 28 days [1], we assign the data points acquired at day 7 to group 1. Assignment of this data to group 2 leads to a worse fit.

4 Fitting

We use densities of labeled primitive cells and labeled of OB cells.

We assume:

- Counts of labeled primitive cells in the SVZ correspond to the sum of labeled aNSC and labeled qNSC.
- Labelled TAPs exist in the model but they do not contribute to the experimentally counted labeled primitive cells in the SVZ.

We assume that r_{max}, β_r, b, p_{stem} and p_{TAP} are not affected by the labeling and assume the values given in Table 1. These values are taken from [1]. We assume that μ and θ do not vary between group one and group two. The number of initially labeled cells, $qNSC_0$, $aNSC_0$, $\sum_{i=0}^{3} TAP_{i,0}$ may be different for both groups. We estimate the unknown parameters using fmincon from MATLAB (The MatWorks, Natic, USA). The cost functional passed to fmincon is a weighted least square functional with the inverse of the standard deviations as weights. We assume that the standard deviation of the labeled NSC count in group 1 at 35 days is equal to the standard deviation of the labeled NSC count in group 2 at 35 days. We choose a multi-start approach with Latin hypercube sampling.

Our stem cell data is given per mm^2 of SVZ. Since there exist around 1000 NSC per mm^2 [8], we set an upper bound of 1000 for the initial number of labeled NSC.

The data from [1] imply that there exist approximately 5000 TAPs per 1000 NSC (standard deviation 1000) at the age where the experiments start. For the fitting we set an upper bound of 7000 TAPs per 1000 NSC.

According to the model in [1] at an age of 56 days approx. 69% of the NSC are quiescent and 31% are active. We assume that active and quiescent NSC are labeled with the same probability. Assuming different labeling probabilities for active and quiescent cells increases the number of free parameters and leads to a worse AIC_c value ($\Delta AIC_c > 10$).

The fitted parameters are provided in Table 2. The fit is depicted in Figure 4. Assuming that the labeling does not affect cell kinetics and that the observed heterogeneity comes from different numbers of initially labeled NSC and TAP, we obtain that in group 1 approx. 57% of the NSC are labeled. This corresponds to approximately 393 qNSC per mm^2 and 177

parameter	value
$aNSC_0 + qNSC_0$	group 1: 570.6, group 2: 261.8
$\sum_{i=0}^{3} TAP_{i,0}$	group 1: 7000, group 2: 0.4
θ	6.7d
μ	0.1m

Table 2: Parameters obtained from the fit of the model to the experimental data. The model was fit to data from both groups simultaneously Only the number of initially labeled cells was allowed to be different for both groups. Other parameters are taken from [1].

aNSC per mm^2. In group 2 approximately 26% of NSC are labeled. This corresponds to approximately 179 qNSC per mm^2 and 81 aNSC per mm^2.

In the model we neglect the time between transduction and label expression. Shortly after the beginning of the experiment the number of experimentally detected labeled cells may differ from the labeled cell counts predicted by the model, since the cells transduced at day zero and counted as labelled cells in the model may not yet express sufficient label concentrations to be detected.

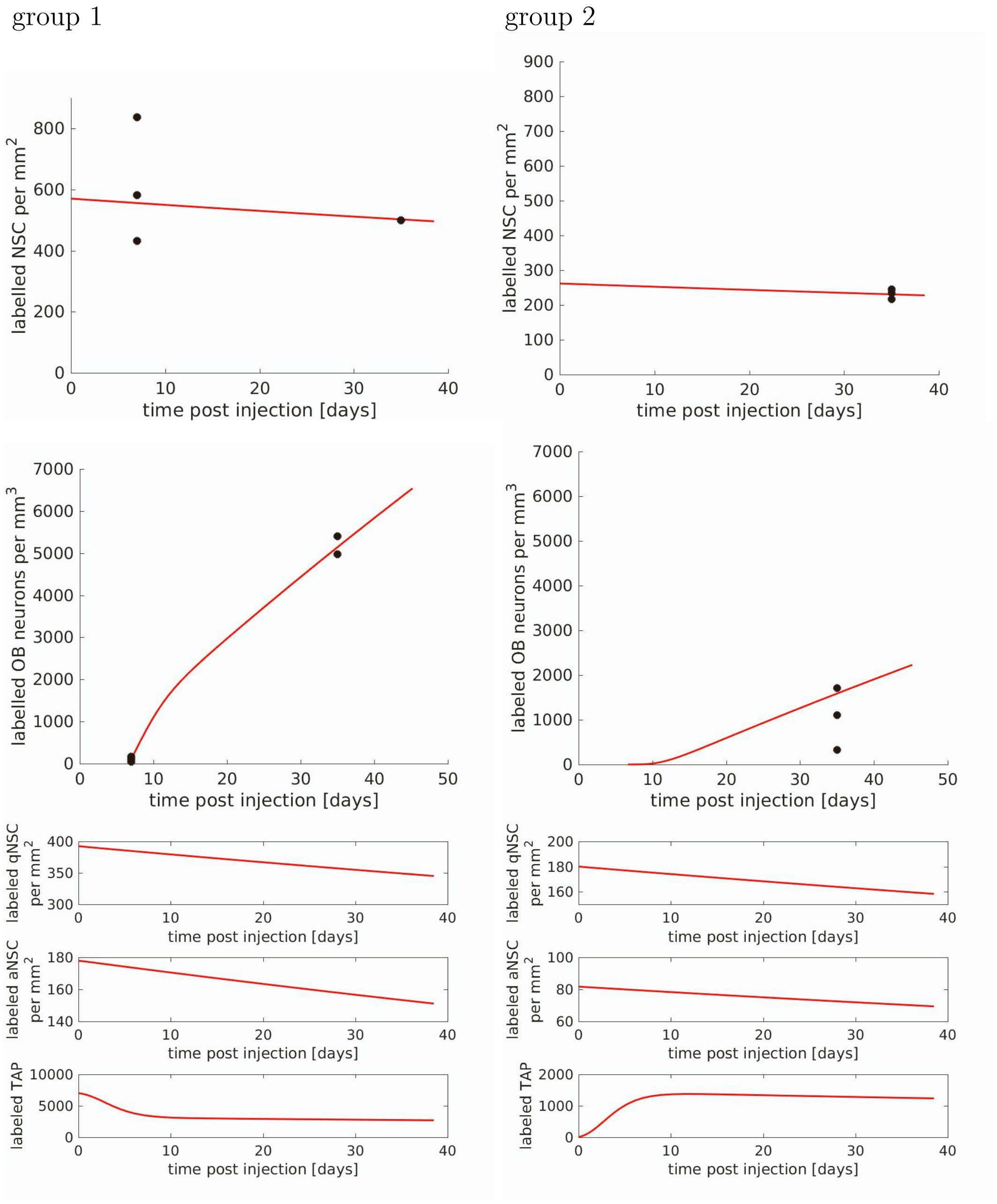

Figure 4: Comparison of model fit and data. The left column compares the fit to data from group 1, the right column to data from group 2. The model was fit to both groups simultaneously. Only the number of initially labeled NSC and TAP differs between the groups.

References

[1] Kalamakis G, Brüne D, Ravichandran S, Bolz J, Fan W, Ziebell F, Stiehl T, Catala-Martinez F, Kupke J, Zhao S, Llorens-Bobadilla E, Bauer K, Limpert S, Berger B, Christen U, Schmezer P, Mallm JP, Berninger B, Anders S, Del Sol A, Marciniak-Czochra A, Martin-Villalba A. Quiescence Modulates Stem Cell Maintenance and Regenerative Capacity in the Aging Brain. *Cell.* 2019, 176(6):1407-1419.

[2] Ziebell F, Martin-Villalba A, Marciniak-Czochra A. Mathematical modelling of adult hippocampal neurogenesis: effects of altered stem cell dynamics on cell counts and bromodeoxyuridine-labelled cells. *J R Soc Interface.* 2014, 11(94):20140144.

[3] Ziebell F, Dehler S, Martin-Villalba A, Marciniak-Czochra A. Revealing age-related changes of adult hippocampal neurogenesis using mathematical models. Development. 2018, 145(1). pii: dev153544.

[4] Ponti G, Obernier K, Guinto C, Jose L, Bonfanti L, Alvarez-Buylla A. Cell cycle and lineage progression of neural progenitors in the ventricular-subventricular zones of adult mice. *Proc Natl Acad Sci U S A.* 2013, 110(11):E1045-54.

[5] Marciniak-Czochra A, Stiehl T, Ho AD, Jäger W, Wagner W. Modeling of asymmetric cell division in hematopoietic stem cells–regulation of self-renewal is essential for efficient repopulation.*Stem Cells Dev.* 2009, 18(3):377-85.

[6] Stiehl T, Marciniak-Czochra A. Characterization of stem cells using mathematical models of multistage cell lineages. *Math Comput Modeling.* 2011, 53(7-8): 1505-1517.

[7] Stiehl T, Marciniak-Czochra A. Stem cell self-renewal in regeneration and cancer: Insights from mathematical modeling. *Curr Opinion Systems Biology.* 2018, 5: 112-120.

[8] Shook BA, Manz DH, Peters JJ, Kang S, Conover JC. Spatiotemporal changes to the subventricular zone stem cell pool through aging. *J Neurosci.* 2012, 32(20):6947-56.

Supplemental Material

Supplemental tables

Supplementary Table 1: Primers and probes used for PCR/qPCR

**Name Primer/Probe**	**Sequence**
Bar_fwd	5’-ATCACTCTCGGCATGGACGAGC-3’
Bar_rev	5’-GGCTGGCAACTAGAAGGCACA-3’
Cre_fwd	5‘-ACTGACGGTGGGAGAATGTT-3‘
Cre_probe	5’ FAM-ACCTGCGGTGCTAACCAGCGT-BHQ1 3’
Cre_rev	5‘-CCAGGCTAAGTGCCTTCTCT-3‘
GFP_fwd	5‘-GAGCGCACCATCTTCTTCAAG-3‘
GFP_probe	5’ FAM-ACGACGGCAACTACA-BHQ1 3’
GFP_rev	5‘-TGTCGCCCTCGAACTTCAC-3‘
ISPCR oligo	5’-AAGCAGTGGTATCAACGCAGAGT-3’
Locked Nucleic Acid-TSO	5’-iCiGiCAAGCAGTGGTATCAACGCAGAGTACATrGrG{G}-3’
Oligo-dT	5’-AAGCAGTGGTATCAACGCAGAGTACT$_{30}$VN-3’

Supplementary Table 2: Sequenced multiplex after Library1 transduction

	Celltype	Set	Sample Number	Total Cell Number	Cycle number
Library 1	qNSCs	Set1	1	443	15
Library 1	aNSCs	Set1	1	500	15
			2	500	15
Library 1	TAPs	Set1	1	500	15
			2	500	15
			3	400	15
Library 1	Neuroblasts	Set1	1	500	15
			2	500	15
			3	500	15
Library 1	qNSCs	Set 2	1	500	15
			2	500	15
			3	500	15
			4	500	15
			5	500	15
Library 1	aNSCs	Set 2	1	500	15
			2	500	15
			3	500	15
			4	500	15
			5	500	15
			6	500	15
Library 1	TAPs	Set 2	1	500	15
			2	500	15
			3	500	15
			4	500	15
Library 1	Neuroblasts	Set 2	1	500	15
			2	500	15
			3	500	15
			4	500	15
			5	500	15
Library 1	qNSCs	Set 3	1	500	15
			2	500	15
			3	500	15
Library 1	aNSCs	Set 3	1	500	15
			2	500	15
			3	500	15
			4	500	15
			5	500	15
Library 1	TAPs	Set 3	1	500	15
			2	500	15
			3	500	15
Library 1	Neuroblasts	Set 3	1	500	15
			2	500	15
			3	500	15
			4	500	15
			5	500	15
Library 1	Astrocytes	Set 4	1	500	15
			2	500	15
			3	500	15
			4	500	15
			5	500	15
Library 1	Oligodendrocytes	Set 4	1	500	15
			2	500	15
			3	500	15
			4	500	15
			5	500	15
Library 1	Ependymal cells	Set 4	1	500	15
Library 1	Astrocytes	Set 5	1	500	15
			2	500	15
			3	500	15
			4	500	15
			5	500	15
			6	500	15
Library 1	Oligodendrocytes	Set 5	1	500	15
			2	500	15
			3	500	15
			4	500	15
			5	500	15
			6	500	15
Library 1	Ependymal cells	Set 5	1	125	16
Library 1	Astrocytes	Set 6	1	500	15
			2	500	15
			3	500	15
			4	500	15
Library 1	Oligodendrocytes	Set 6	1	500	15
			2	500	15
			3	500	15
			4	268	16
Library 1	Ependymalcells	Set 6	1	199	16
Library 1	In vitro cultured NSCs	Set1	1	1500	14
Library 1	In vitro cultured NSCs	Set2	1	1500	14

Supplementary Table 3: Sequenced multiplex after Library3 transduction

	Celltype	Set	Batch Number	Cell Numbers	Cycle number
Library 3	qNSCs	Set1	1	500	15
			2	474	15
Library 3	aNSCs	Set1	1	500	15
			2	500	15
			3	380	15
Library 3	TAPs	Set1	1	500	15
Library 3	Neuroblasts	Set1	1	500	15
			2	500	15
			3	500	15
Library 3	qNSCs	Set2	1	500	15
			2	369	15
Library 3	aNSC	Set2	1	500	15
			2	500	15
			3	500	15
			4	345	15
Library 3	qNSC	Set3	1	500	15
			2	500	15
			3	415	15
Library 3	aNSCs	Set3	1	500	15
			2	500	15
			3	500	15
			4	376	15
Library 3	TAPs	Set3	1	500	15
			2	500	15
			3	500	15
			4	500	15
			5	500	15
Library 3	Neuroblasts	Set3	1	500	15
			2	500	15
			3	500	15
			4	500	15
			5	500	15
Library 3	Astrocytes	Set4	1	500	15
			2	500	15
			3	500	15
			4	500	15
			5	500	15
Library 3	Oligodendrocytes	Set4	1	500	15
			2	500	15
			3	500	15
			4	500	15
			5	500	15
Library 3	Ependymal cells	Set4	1	500	15
			2	500	15
			3	339	15
Library 3	Astrocytes	Set5	1	500	15
			2	500	15
			3	500	15
			4	500	15
			5	500	15
Library 3	Oligodendrocytes	Set5	1	500	15
			2	500	15
			3	500	15
			4	500	15
			5	500	15
Library 3	Ependymal cells	Set5	1	450	15
Library 3	Astrocytes	Set6	1	500	15
			2	500	15
			3	500	15
			4	500	15
			5	500	15
Library 3	Oligodendrocytes	Set6	1	500	15
			2	500	15
			3	500	15
			4	500	15
			5	500	15
Library 3	Ependymal cells	Set6	1	210	16
Library 3	In vitro cultured NSCs	Set1	1	1500	14
Library 3	In vitro cultured NSCs	Set1	1	1500	14

Supplementary Table S4: AAV variants used in library #1 and library #3. Modified from the accompanying manuscript by Weinmann *et al.*

Variant	Source	Library1	Library3
AAV1_wt	(Hoggan, Blacklow, & Rowe, 1966a)	✔	✔
AAV1_A1	Dirk Grimm Laboratory	✔	✔
AAV1_A2	Dirk Grimm Laboratory	✔	✔
AAV1_A6	Dirk Grimm Laboratory	✔	✔
AAV1_P2	Dirk Grimm Laboratory	✔	✖
AAV1_P4	Dirk Grimm Laboratory	✔	✔
AAV1_P5	Dirk Grimm Laboratory	✔	✔
AAV2_wt	(Hoggan, Blacklow, & Rowe, 1966b)	✔	✔
AAV2_A1	Dirk Grimm Laboratory	✔	✔
AAV2_A2	Dirk Grimm Laboratory	✔	✔
AAV2_A6	Dirk Grimm Laboratory	✔	✔
AAV2_P2	Dirk Grimm Laboratory	✔	✔
AAV2_P4	Dirk Grimm Laboratory	✔	✔
AAV2_P5	Dirk Grimm Laboratory	✔	✔
AAV2_7m8	(Dalkara et al., 2013)	✖	✔
AAV2_BR1	(Körbelin, Dogbevia, et al., 2016)	✖	✔
AAV2_L1	(Körbelin, Sieber, et al., 2016)	✖	✔
AAV2_L1mut1	(Körbelin, Sieber, et al., 2016)	✖	✔
AAV2_L1mut2	(Körbelin, Sieber, et al., 2016)	✖	✔
AAV2_MTP	(Yu et al., 2009)	✖	✔
AAV2HBKO	(Opie, Warrington, Agbandje-McKenna, Zolotukhin, & Muzyczka, 2003)	✖	✔
AAV2YF	(Li et al., 2010)	✖	✔
AAV3b_wt	(Rutledge, Halbert, & Russell, 1998)	✔	✔
AAV3b_A1	Dirk Grimm Laboratory	✔	✔
AAV3b_A2	Dirk Grimm Laboratory	✔	✔
AAV3b_A6	Dirk Grimm Laboratory	✔	✔
AAV3b_P2	Dirk Grimm Laboratory	✔	✖
AAV3b_P4	Dirk Grimm Laboratory	✔	✔
AAV3b_P5	Dirk Grimm Laboratory	✔	✔

AAV4_wt	(Parks, Melnick, Rongey, & Mayor, 1967)	✓	✓
AAV4_A1	Dirk Grimm Laboratory	✓	✓
AAV4_A2	Dirk Grimm Laboratory	✓	✓
AAV4_A6	Dirk Grimm Laboratory	✓	✓
AAV4_P2	Dirk Grimm Laboratory	✓	✓
AAV4_P4	Dirk Grimm Laboratory	✓	✓
AAV4_P5	Dirk Grimm Laboratory	✓	✓
AAV4_L1	Dirk Grimm Laboratory	✗	✓
AAV4mut_wt	Dirk Grimm Laboratory	✓	✗
AAV4mut_A1	Dirk Grimm Laboratory	✓	✗
AAV4mut_A2	Dirk Grimm Laboratory	✓	✗
AAV4mut_A6	Dirk Grimm Laboratory	✓	✗
AAV4mut_P2	Dirk Grimm Laboratory	✓	✗
AAV4mut_P4	Dirk Grimm Laboratory	✓	✗
AAV4mut_P5	Dirk Grimm Laboratory	✓	✗
AAV5_wt	(Bantel-Schaal & Zur Hausen, 1984)	✓	✓
AAV5_A1	Dirk Grimm Laboratory	✓	✓
AAV5_A2	Dirk Grimm Laboratory	✓	✓
AAV5_A6	Dirk Grimm Laboratory	✓	✓
AAV5_P2	Dirk Grimm Laboratory	✓	✗
AAV5_P4	Dirk Grimm Laboratory	✓	✓
AAV5_P5	Dirk Grimm Laboratory	✓	✓
AAV6_wt	(Rutledge et al., 1998)	✓	✓
AAV6_A1	Dirk Grimm Laboratory	✓	✗
AAV6_A2	Dirk Grimm Laboratory	✓	✗
AAV6_A6	Dirk Grimm Laboratory	✓	✗
AAV6_P2	Dirk Grimm Laboratory	✓	✗
AAV6_P4	Dirk Grimm Laboratory	✓	✓
AAV6_P5	Dirk Grimm Laboratory	✓	✗
AAV6.2	(Limberis, Vandenberghe, Zhang, Pickles, & Wilson, 2009)	✗	✓

AAV7_wt	(G.-P. Gao et al., 2002)	✓	✓
AAV7_A1	Dirk Grimm Laboratory	✓	✓
AAV7_A2	Dirk Grimm Laboratory	✓	✓
AAV7_A6	Dirk Grimm Laboratory	✓	✓
AAV7_P2	Dirk Grimm Laboratory	✓	✓
AAV7_P4	Dirk Grimm Laboratory	✓	✓
AAV7_P5	Dirk Grimm Laboratory	✓	✓
AAV8_wt	(G.-P. Gao et al., 2002)	✓	✓
AAV8__A1	Dirk Grimm Laboratory	✓	✓
AAV8__A2	Dirk Grimm Laboratory	✓	✓
AAV8_A6	Dirk Grimm Laboratory	✓	✓
AAV8_P2	Dirk Grimm Laboratory	✓	✓
AAV8_P4	Dirk Grimm Laboratory	✓	✓
AAV8_P5	Dirk Grimm Laboratory	✓	✓
AAV9_wt	(G. Gao et al., 2004)	✓	✓
AAV9_A1	Dirk Grimm Laboratory	✓	✓
AAV9_A2	Dirk Grimm Laboratory	✓	✓
AAV9_A6	Dirk Grimm Laboratory	✓	✓
AAV9_P1	(Kunze et al., 2018)	✗	✓
AAV9_P2	Dirk Grimm Laboratory	✓	✗
AAV9_P3	Dirk Grimm Laboratory	✗	✓
AAV9_P4	Dirk Grimm Laboratory	✓	✓
AAV9_P5	Dirk Grimm Laboratory	✓	✓
AAV9_BR1	Dirk Grimm Laboratory	✗	✓
AAV9_K1	(Varadi et al., 2012)	✗	✓
AAV9_K3	(Varadi et al., 2012)	✗	✓
AAV9K449R_PHP.A	(Deverman et al., 2016)	✗	✓
AAV9K449R_PHP.B	(Deverman et al., 2016)	✗	✓
AAV9K449R_PHP.eB	(Chan et al., 2017)	✗	✓
AAV9K449R_PHP.S	(Chan et al., 2017)	✗	✓
AAV9BI	Boehringer Ingelheim	✗	✓

AAV9LD	(Adachi, Enoki, Kawano, Veraz, & Nakai, 2014)	✗	✓
AAV10_wt	(G. Gao et al., 2004)	✓	✓
AAV10_A1	Dirk Grimm Laboratory	✓	✓
AAV10_A2	Dirk Grimm Laboratory	✓	✓
AAV10_A6	Dirk Grimm Laboratory	✓	✓
AAV10_P2	Dirk Grimm Laboratory	✓	✓
AAV10_P4	Dirk Grimm Laboratory	✓	✓
AAV10_P5	Dirk Grimm Laboratory	✓	✓
AAVpo1_wt	(Bello et al., 2009)	✓	✓
AAVpo1_A1	Dirk Grimm Laboratory	✓	✓
AAVpo1_A2	Dirk Grimm Laboratory	✓	✓
AAVpo1_A6	Dirk Grimm Laboratory	✓	✓
AAVpo1_P2	Dirk Grimm Laboratory	✓	✗
AAVpo1_P4	Dirk Grimm Laboratory	✓	✓
AAVpo1_P5	Dirk Grimm Laboratory	✓	✓
AAV12_wt	(Schmidt et al., 2008)	✓	✓
AAV12_A1	Dirk Grimm Laboratory	✓	✗
AAV12_A2	Dirk Grimm Laboratory	✓	✗
AAV12_A6	Dirk Grimm Laboratory	✓	✗
AAV12_P2	Dirk Grimm Laboratory	✓	✗
AAV12_P4	Dirk Grimm Laboratory	✓	✗
AAV12_P5	Dirk Grimm Laboratory	✓	✗
AAVAnc80L65	(Zinn et al., 2015)	✗	✓
AAVB1	(Choudhury et al., 2016)	✗	✓
AAVDJ	(Grimm et al., 2008)	✗	✓
AAVDJYF	Dirk Grimm Laboratory	✗	✓
AAVLK03	(Lisowski et al., 2014)	✗	✓
AAVM41	(Yang et al., 2009)	✗	✓
AAVshH10	(Klimczak, Koerber, Dalkara, Flannery, & Schaffer, 2009)	✗	✓
AAVAH4-N9	Dirk Grimm Laboratory	✗	✓

AAVAH4-N7	Dirk Grimm Laboratory	✗	✓
AAVAH4-N6	Dirk Grimm Laboratory	✗	✓
AAVAH4-N5	Dirk Grimm Laboratory	✗	✓
AAVAH4-N4	Dirk Grimm Laboratory	✗	✓
AAVAH4-N3	Dirk Grimm Laboratory	✗	✓
AAVAH4-N12	Dirk Grimm Laboratory	✗	✓
AAVAH4-N11	Dirk Grimm Laboratory	✗	✓
AAVAH4-N1	Dirk Grimm Laboratory	✗	✓
AAVAH4b-10	Dirk Grimm Laboratory	✗	✓
AAVAH4-7	Dirk Grimm Laboratory	✗	✓
AAVAH4-6	Dirk Grimm Laboratory	✗	✓
AAVAH4-3	Dirk Grimm Laboratory	✗	✓
AAVAH4-1	Dirk Grimm Laboratory	✗	✓
AAVAH3-N9	Dirk Grimm Laboratory	✗	✓
AAVAH3-N8	Dirk Grimm Laboratory	✗	✓
AAVAH3-N6	Dirk Grimm Laboratory	✗	✓
AAVAH3-N5	Dirk Grimm Laboratory	✗	✓
AAVAH3-N4	Dirk Grimm Laboratory	✗	✓
AAVAH3-N2	Dirk Grimm Laboratory	✗	✓
AAVAH3-N12	Dirk Grimm Laboratory	✗	✓
AAVAH3-N10	Dirk Grimm Laboratory	✗	✓
AAVAH3-N1	Dirk Grimm Laboratory	✗	✓
AAVAH3-5	Dirk Grimm Laboratory	✗	✓
AAVAH3-3	Dirk Grimm Laboratory	✗	✓
AAVAH3-21	Dirk Grimm Laboratory	✗	✓
AAVAH3-18	Dirk Grimm Laboratory	✗	✓
AAVAH3-17	Dirk Grimm Laboratory	✗	✓
AAVAH3-10	Dirk Grimm Laboratory	✗	✓
AAVJEA3-S8	Dirk Grimm Laboratory	✗	✓
AAVJEA3-S7	Dirk Grimm Laboratory	✗	✓
AAVJEA3-S5	Dirk Grimm Laboratory	✗	✓
AAVJEA3-S4	Dirk Grimm Laboratory	✗	✓

AAVJEA3-S2	Dirk Grimm Laboratory	✗	✓
AAVJEA3-S10	Dirk Grimm Laboratory	✗	✓
AAVJEA3-S1	Dirk Grimm Laboratory	✗	✓
AAVJEA3-H5	Dirk Grimm Laboratory	✗	✓
AAVJEA3-H4	Dirk Grimm Laboratory	✗	✓
AAVJEA3-H3	Dirk Grimm Laboratory	✗	✓
AAVJEA3-H20	Dirk Grimm Laboratory	✗	✓
AAVJEA3-H19	Dirk Grimm Laboratory	✗	✓
AAVJEA3-H15	Dirk Grimm Laboratory	✗	✓
AAVJEA3-H13	Dirk Grimm Laboratory	✗	✓
AAVJEA3-D7	Dirk Grimm Laboratory	✗	✓
AAVJEA3-D5	Dirk Grimm Laboratory	✗	✓
AAVJEA3-D4	Dirk Grimm Laboratory	✗	✓
AAVJEA3-D20	Dirk Grimm Laboratory	✗	✓
AAVJEA3-D2	Dirk Grimm Laboratory	✗	✓
AAVJEA3-D16	Dirk Grimm Laboratory	✗	✓
AAVJEA3-D12	Dirk Grimm Laboratory	✗	✓
AAVJEA2-S11	Dirk Grimm Laboratory	✗	✓
AAVJEA2-H8	Dirk Grimm Laboratory	✗	✓
AAVJEA2-H2	Dirk Grimm Laboratory	✗	✓
AAVJEA2-H17	Dirk Grimm Laboratory	✗	✓
AAVJEA2-H11	Dirk Grimm Laboratory	✗	✓
AAVJEA2-H1	Dirk Grimm Laboratory	✗	✓
AAVJEA2-D7	Dirk Grimm Laboratory	✗	✓
AAVJEA2-D3	Dirk Grimm Laboratory	✗	✓
AAVJEA2-D16	Dirk Grimm Laboratory	✗	✓
AAVJEA2-D10	Dirk Grimm Laboratory	✗	✓
AAVJEA2-D1	Dirk Grimm Laboratory	✗	✓

Supplementary Table S5_differentially-expressed-genes

geneSymbol	geneID	foldChange	log2FoldChange	meanExpression	adjustedPvalue
**Sparcl1**	ENSMUSG00000029309.7	0.4586443309084443	-1.1245522875247096	569.6949982621883	5.481135346975202e-4
**Nr2f1**	ENSMUSG00000069171.14	0.563400502846002	-0.8277672435403163	81.34569380445812	0.002657252798171417
**A830082K12Rik**	ENSMUSG00000087143.9	0.6435593143024395	-0.6358549728494258	118.01421227043072	0.010666606992532435
**Ntsr2**	ENSMUSG00000020591.11	0.564793019355379	-0.8242058373753183	196.08528815354265	0.011409209023240174
**Fjx1**	ENSMUSG00000075012.4	0.6591223494942214	-0.6013818047299784	160.55578280494055	0.01369812888728773
**Cdh19**	ENSMUSG00000047216.8	0.5153151771265769	-0.9564730113372258	62.700375544987914	0.019105903951354777
**Trps1**	ENSMUSG00000038679.16	0.7828015113716924	-0.35328155347765244	480.71259614096226	0.026182598611121745
**Ncald**	ENSMUSG00000051359.15	0.5974398127275874	-0.7431347144351399	40.6614874099513	0.026182598611121745
**Adgrl3**	ENSMUSG00000037605.16	0.7417745392837614	-0.4309473453525118	453.20421824442883	0.027474332239434
**Pla2g7**	ENSMUSG00000023913.17	0.5039862617870257	-0.9885436871976164	208.61311157145997	0.032032920538555325
**Plpp3**	ENSMUSG00000028517.8	0.718722646538464	-0.4764929496184573	876.0664633949602	0.032032920538555325
**Rora**	ENSMUSG00000032238.17	0.7871722104845883	-0.34524880472817765	682.6794750293336	0.032032920538555325
**Nkain2**	ENSMUSG00000069670.8	0.6475559011785621	-0.6269233540233836	294.28899085669553	0.04750224896887462
**Rmst**	ENSMUSG00000112117.1	0.549166725361834	-0.8646838812919768	106.73728603443496	0.04750224896887462
**Abr**	ENSMUSG00000017631.18	0.7122887560091385	-0.4894658783999692	115.36379717416378	0.04854367969120873
**Zhx2**	ENSMUSG00000071757.10	0.6853963040813458	-0.5449896825719882	94.5402551032734	0.04854367969120873
**Shisa9**	ENSMUSG00000022494.15	0.3075413864236202	-1.7011475251820585	42.884210839461865	0.04854367969120873
**Ptprt**	ENSMUSG00000053141.16	0.5883901763676116	-0.7651549350112068	377.971608777912	0.04854367969120873

Supplementary Table S6_input-library-info

Variant	Barcode	PercentOfLibrary	Library
**AAV12_A1**	TGTTTAGGTGAGCCT	0.003466195	Library #1
**AAV12_A2**	TGTGGTGTGACTCAG	0.0011020722	Library #1
**AAV12_A6**	TCGGGTTGGTCTTTG	0.001116885	Library #1
**AAV12_P2**	AGCCTAATCTTTGAC	0.0144365539	Library #1
**AAV12_P4**	AAGCACTAAAGAACA	0.011716924	Library #1
**AAV12_P5**	GGTATGGCCTGCCGC	0.0035017457	Library #1
**AAV12_WT**	GTAGCTGAGGTTGGT	0.2081672263	Library #1
**AAV1_A1**	TTGCCGTCCTTCGAG	0.2329845897	Library #1
**AAV1_A2**	TTCAGCGGACGGGCC	0.0506064464	Library #1
**AAV1_A6**	GTCAGTCCGCTCTTT	0.0923489138	Library #1
**AAV1_P2**	TAGAGATTTAAACCG	0.008250729	Library #1
**AAV1_P4**	CGTGACAGCGGATGG	0.3035231383	Library #1
**AAV1_P5**	TGGGCGGTCAGGGTC	0.4734644556	Library #1
**AAV1_WT**	AGACTCGTTGTATAT	0.8238582524	Library #1
**AAV2_A1**	GTGCTTCTGGCGGAT	1.0157254748	Library #1
**AAV2_A2**	CGGCTGTCGGTCGCC	0.8408692707	Library #1
**AAV2_A6**	ATCGTACGTTACTGA	0.803570643	Library #1
**AAV2_P2**	TCAACATGGGCAACG	0.4276218054	Library #1
**AAV2_P4**	CTTGATCGACGCCCA	0.7037175652	Library #1
**AAV2_P5**	TACGCTATTCAATCT	0.6199926629	Library #1
**AAV2_WT**	TTAAGATCCTGGTCG	1.9217710609	Library #1
**AAV3_A1**	TAACGTTGGGTTGCC	1.1127611582	Library #1
**AAV3_A2**	GACCACTAGAAGGGC	0.8236893865	Library #1
**AAV3_A6**	CTGCATGGCGGAGTT	1.3856958666	Library #1
**AAV3_P2**	CGTATCGGGTCCGGA	0.1889728014	Library #1
**AAV3_P4**	TGGTTGGGTTTGTGG	1.6613146543	Library #1
**AAV3_P5**	TCGTTGTAACGGTAC	1.8278519904	Library #1
**AAV3_WT**	GATTCGAAAGCATAG	2.2215258232	Library #1
**AAV4_A1**	ACCATAGCGCCACGA	0.2361367533	Library #1
**AAV4_A2**	GTCCCGACTAGGACT	0.0979303765	Library #1
**AAV4_A6**	GTCTTGATTGCTTCG	0.2611377954	Library #1
**AAV4mut_A1**	TGAGAGTCATCCAAG	0.3431651509	Library #1

**AAV4mut_A2**	CCTAATCTCAGGCGG	3.110688e-4	Library #1
**AAV4mut_A6**	CGTGACCCAGGAAGT	0.4054055695	Library #1
**AAV4mut_P2**	AGACTTGGCGTTATG	0.1218767473	Library #1
**AAV4mut_P4**	ACGTGTCGTAGTAAG	0.4234949596	Library #1
**AAV4mut_P5**	TATATTGAGGCGTGT	0.4400645565	Library #1
**AAV4mut_WT**	ACATTGTGGTCATAG	0.4698797583	Library #1
**AAV4_P2**	TGGTAGGTTCGAAAT	0.1096443379	Library #1
**AAV4_P4**	ACGTCGCACCGTTTG	0.507003595	Library #1
**AAV4_P5**	CAGGCTTAACGCGGG	0.3978510421	Library #1
**AAV4_WT**	TCAACGATTGTCTGG	0.3478311826	Library #1
**AAV5_A1**	TTGGACTCACAGATG	4.1719685377	Library #1
**AAV5_A2**	AAGGTGACCTAGTGT	3.8578127723	Library #1
**AAV5_A6**	CCCTCATGAGGTCCG	4.2980550825	Library #1
**AAV5_P2**	GGCCACCGTGTGTGA	0.0616745697	Library #1
**AAV5_P4**	ATGAGCAGCGAATGA	4.6984124501	Library #1
**AAV5_P5**	ATGTTTAACGGCATA	5.3407398513	Library #1
**AAV5_WT**	ATTTGGCACAGGATG	3.9133785438	Library #1
**AAV6_A1**	GTTAACGCGGCCATT	0.0071634696	Library #1
**AAV6_A2**	AGCGGCGTTTATCGT	0.0059191945	Library #1
**AAV6_A6**	TTGGTATGTGTCAAT	0.0210252868	Library #1
**AAV6_P2**	GCGAGGTCGTTAGTT	0.0036913495	Library #1
**AAV6_P4**	TAAGACTGTTCCGGG	0.0389132229	Library #1
**AAV6_P5**	GTTTGTAATCTCTAC	0.0330740175	Library #1
**AAV6_WT**	ATGACAATGTGCAGG	0.1905785088	Library #1
**AAV7_A1**	ACGATCGTACGTCTT	1.3022968459	Library #1
**AAV7_A2**	GTTCAGGTCAGGTCT	0.9521726421	Library #1
**AAV7_A6**	TAAGGAGGGCTGTAG	1.0177489031	Library #1
**AAV7_P2**	GAGCGTAATTGTGAG	0.7489469655	Library #1
**AAV7_P4**	CGTTAACCCGAAAGC	1.2039842993	Library #1
**AAV7_P5**	GTGACATGCAGGTAG	1.2799295195	Library #1
**AAV7_WT**	GTCGACTTCATGGCA	2.2909711872	Library #1
**AAV8_A1**	GGGCCCTAGCGCGTG	0.5194019078	Library #1
**AAV8_A2**	GATAGGCTGGTCCAA	0.4331706799	Library #1
**AAV8_A6**	TATTTGTGTCGTTCC	0.4140769821	Library #1

**AAV8_P2**	GCTCTGGATGTAGTA	0.1294609003	Library #1
**AAV8_P4**	TAGATGTGGCGGACA	0.6946550948	Library #1
**AAV8_P5**	GTCAACATCGTTACA	0.6880722869	Library #1
**AAV8_WT**	TATCAAGCTAACGTT	0.8518870306	Library #1
**AAV9_A1**	GCCGGAGTCCCGGTA	3.1439484398	Library #1
**AAV9_A2**	CGAGTCGTATGTGGC	2.5158502133	Library #1
**AAV9_A6**	AGTAATTGGTCTTGG	3.5015234816	Library #1
**AAV9_P2**	GCGGAACATAGGCGG	0.218802816	Library #1
**AAV9_P4**	GCCCTTCAGTCAGCT	4.4970294857	Library #1
**AAV9_P5**	CGGTCGCGTGACGTG	3.8997418811	Library #1
**AAV9_WT**	AGTTAGGGCGCTGCG	5.411580581	Library #1
**AAVpo1_A1**	TTGGAACGTGGGCTT	1.7915784083	Library #1
**AAVpo1_A2**	AGATTCAAAGCTGCG	0.9802014203	Library #1
**AAVpo1_A6**	TGTTGGAAGGTATCA	1.728911381	Library #1
**AAVpo1_P2**	TGTCCGGAAAGGACA	0.0635291322	Library #1
**AAVpo1_P4**	GTTGTGCCCTGAGTG	2.8682881764	Library #1
**AAVpo1_P5**	ACCGTATCTCTCCGG	2.2795416316	Library #1
**AAVpo1_WT**	TGGTTTACAAATTAT	1.8485276952	Library #1
**AAVrh10_A1**	CTACCTATTTACTCT	0.505166808	Library #1
**AAVrh10_A2**	ACCGGGCGTTGAGGC	0.6713041985	Library #1
**AAVrh10_A6**	ACTGTGATGGGTTAG	0.612764017	Library #1
**AAVrh10_P2**	GACTTGGTTGTGACG	0.0944789943	Library #1
**AAVrh10_P4**	TTGTTGTATGAGCAG	0.6312622404	Library #1
**AAVrh10_P5**	TCCACGGAGGCTGCG	0.6370421945	Library #1
**AAVrh10_WT**	GGTCTTTGCTCGGTG	0.9843875173	Library #1
**AAV12_WT**	GTAGCTGAGGTTGGT	0.8797413038	Library #3
**AAV1_A1**	TTGCCGTCCTTCGAG	1.0446285517	Library #3
**AAV1_A2**	TTCAGCGGACGGGCC	0.3012612638	Library #3
**AAV1_A6**	GTCAGTCCGCTCTTT	0.9191866476	Library #3
**AAV1_P4**	CGTGACAGCGGATGG	1.1194400931	Library #3
**AAV1_P5**	TGGGCGGTCAGGGTC	0.9804635819	Library #3
**AAV1_WT**	AGACTCGTTGTATAT	1.7084198554	Library #3
**AAV2_7m8**	CGTGACCCAGGAAGT	0.7650928206	Library #3
**AAV2_A1**	GTGCTTCTGGCGGAT	0.255705359	Library #3

**AAV2_A2**	CGGCTGTCGGTCGCC	0.2813060806	Library #3
**AAV2_A6**	ATCGTACGTTACTGA	0.3667152439	Library #3
**AAV2_BR1**	ACGTGTCGTAGTAAG	1.0737699722	Library #3
**AAV2HBKO**	CCTAATCTCAGGCGG	1.1479533251	Library #3
**AAV2_L1**	TATATTGAGGCGTGT	0.5147066682	Library #3
**AAV2_L1mut1**	ATGATCAGCGATATC	0.1970553949	Library #3
**AAV2_L1mut2**	GGTGCCGGACAGCTC	0.2362233568	Library #3
**AAV2_MTP**	AGCGGCGTTTATCGT	0.5277517778	Library #3
**AAV2_P2**	TCAACATGGGCAACG	0.6886006709	Library #3
**AAV2_P4**	CTTGATCGACGCCCA	0.4527444373	Library #3
**AAV2_P5**	TACGCTATTCAATCT	0.6625186101	Library #3
**AAV2_WT**	TTAAGATCCTGGTCG	0.3617305272	Library #3
**AAV2YF**	CTTATGTGAAGAGAT	0.6318352723	Library #3
**AAV3_A1**	TAACGTTGGGTTGCC	0.5328343939	Library #3
**AAV3_A2**	GACCACTAGAAGGGC	0.1884483964	Library #3
**AAV3_A6**	CTGCATGGCGGAGTT	0.0984134813	Library #3
**AAV3_P4**	TGGTTGGGTTTGTGG	0.622591927	Library #3
**AAV3_P5**	TCGTTGTAACGGTAC	0.8738754916	Library #3
**AAV3_WT**	GATTCGAAAGCATAG	0.7399326468	Library #3
**AAV4_A1**	ACCATAGCGCCACGA	0.3741556065	Library #3
**AAV4_A2**	GTCCCGACTAGGACT	0.2107042181	Library #3
**AAV4_A6**	GTCTTGATTGCTTCG	0.4466501929	Library #3
**AAV4_L1**	GGTATGGCCTGCCGC	0.456668576	Library #3
**AAV4_P2**	TGGTAGGTTCGAAAT	3.4295503304	Library #3
**AAV4_P4**	ACGTCGCACCGTTTG	0.7385620536	Library #3
**AAV4_P5**	CAGGCTTAACGCGGG	0.2088359691	Library #3
**AAV4_WT**	TCAACGATTGTCTGG	0.269656039	Library #3
**AAV5_A1**	TTGGACTCACAGATG	0.2461519986	Library #3
**AAV5_A2**	AAGGTGACCTAGTGT	0.2486647527	Library #3
**AAV5_A6**	CCCTCATGAGGTCCG	0.2369576031	Library #3
**AAV5_P4**	ATGAGCAGCGAATGA	0.2925726824	Library #3
**AAV5_P5**	ATGTTTAACGGCATA	0.5152532738	Library #3
**AAV5_WT**	ATTTGGCACAGGATG	0.1734371384	Library #3
**AAV6.2**	TGTGGTGTGACTCAG	2.553529208	Library #3

**AAV6_P4**	TAAGACTGTTCCGGG	2.080568522	Library #3
**AAV6shH10**	AGCCTAATCTTTGAC	0.6027835931	Library #3
**AAV6_WT**	ATGACAATGTGCAGG	0.6671688368	Library #3
**AAV7_A1**	ACGATCGTACGTCTT	0.4132746186	Library #3
**AAV7_A2**	GTTCAGGTCAGGTCT	0.3045653723	Library #3
**AAV7_A6**	TAAGGAGGGCTGTAG	1.0100210753	Library #3
**AAV7_P2**	GAGCGTAATTGTGAG	0.9190724315	Library #3
**AAV7_P4**	CGTTAACCCGAAAGC	0.6356859863	Library #3
**AAV7_P5**	GTGACATGCAGGTAG	0.7268141126	Library #3
**AAV7_WT**	GTCGACTTCATGGCA	0.223145614	Library #3
**AAV8_A1**	GGGCCCTAGCGCGTG	0.4964239349	Library #3
**AAV8_A2**	GATAGGCTGGTCCAA	0.3521037422	Library #3
**AAV8_A6**	TATTTGTGTCGTTCC	0.5162812186	Library #3
**AAV8_P2**	GCTCTGGATGTAGTA	0.553678831	Library #3
**AAV8_P4**	TAGATGTGGCGGACA	0.3955303327	Library #3
**AAV8_P5**	GTCAACATCGTTACA	0.8540997908	Library #3
**AAV8_WT**	TATCAAGCTAACGTT	0.3275309654	Library #3
**AAV9_A1**	GCCGGAGTCCCGGTA	0.4303091332	Library #3
**AAV9_A2**	CGAGTCGTATGTGGC	0.3886447339	Library #3
**AAV9_A6**	AGTAATTGGTCTTGG	0.6598345319	Library #3
**AAV9BI**	ACATTGTGGTCATAG	1.4045071465	Library #3
**AAV9_BR1**	AAGCACTAAAGAACA	0.2648263299	Library #3
**AAV9_K1**	GTTTGTAATCTCTAC	0.3277675559	Library #3
**AAV9_K3**	TTGGTATGTGTCAAT	0.3422158917	Library #3
**AAV9K449R_P**	TCGTTAGTAGCGATC	0.1926417587	Library #3
**AAV9K449R_P**	TGAGAGTCATCCAAG	0.3460013394	Library #3
**AAV9K449R_P**	CGTATCGGGTCCGGA	0.5888900211	Library #3
**AAV9K449R_P**	TAGAGATTTAAACCG	0.4132664604	Library #3
**AAV9LD**	GGCCACCGTGTGTGA	0.5675397698	Library #3
**AAV9_P1**	GCGAGGTCGTTAGTT	0.1599433227	Library #3
**AAV9_P3**	GTTAACGCGGCCATT	0.2386137364	Library #3
**AAV9_P4**	GCCCTTCAGTCAGCT	0.6900365304	Library #3
**AAV9_P5**	CGGTCGCGTGACGTG	0.3978717626	Library #3
**AAV9_WT**	AGTTAGGGCGCTGCG	1.0773025128	Library #3

**AAVAH3-10**	TTCCGTGTGTTGTCT	0.3847450701	Library #3
**AAVAH3-17**	GGACTCAGGCCTGGT	0.3819957255	Library #3
**AAVAH3-18**	CAATCCGGCGCGGGT	0.1895171327	Library #3
**AAVAH3-21**	CCCGTATGTCGGGTA	0.4870174237	Library #3
**AAVAH3-3**	AGTTTCACATGACGG	1.3268646777	Library #3
**AAVAH3-5**	TCGGGTTGGTCTTTG	0.5787003139	Library #3
**AAVAH3-N1**	GGTCAGGACCATTGG	1.2869135197	Library #3
**AAVAH3-N10**	TGGGTTTCGGCATCA	0.8057619082	Library #3
**AAVAH3-N12**	TCGCACGCTGATGTG	0.2506064263	Library #3
**AAVAH3-N2**	TAGTTTATCGCAGGG	0.4156649983	Library #3
**AAVAH3-N4**	GTACCTATCCGTTGT	0.5063607351	Library #3
**AAVAH3-N5**	TGGTCGGCGAGTTTG	0.9596354614	Library #3
**AAVAH3-N6**	ATGTCGAACCCAATC	0.7456108183	Library #3
**AAVAH3-N8**	TTTGGTTGGAGTCTT	0.2409470081	Library #3
**AAVAH3-N9**	AGTTCACGACTGCGA	1.0275858789	Library #3
**AAVAH4-1**	GTTCGTTGCGGGATC	0.8892293979	Library #3
**AAVAH4-3**	GAATCCATGACTTTG	0.6495469252	Library #3
**AAVAH4-6**	GTGTAGGTTATCATC	0.3769131094	Library #3
**AAVAH4-7**	TTACGATTTATGCGC	0.9018258014	Library #3
**AAVAH4b-10**	GTTTACGGATCTCGG	0.8244036064	Library #3
**AAVAH4-N1**	TTACCTTCTAAGGGC	0.414539154	Library #3
**AAVAH4-N11**	TCTGTATGGGCCAGC	0.7840445338	Library #3
**AAVAH4-N12**	TGATCTGACCGTGTG	0.4651287252	Library #3
**AAVAH4-N3**	GGTTGGTTAGGCTGT	0.4590915888	Library #3
**AAVAH4-N4**	ACCGGCAATCCTAGC	0.4980311185	Library #3
**AAVAH4-N5**	GTGTGTTACCTAACA	0.6201689142	Library #3
**AAVAH4-N6**	TCATCTAGCATCGGG	0.4211228959	Library #3
**AAVAH4-N7**	GCCACAGGCATCGTG	0.3821996829	Library #3
**AAVAH4-N9**	TATATAGTCGGTTTG	0.7664797303	Library #3
**AAVAnc80L65**	GCGGAACATAGGCGG	0.5427304026	Library #3
**AAVB1**	TGTCCGGAAAGGACA	0.4654387403	Library #3
**AAVDJ**	AGACTTGGCGTTATG	0.6410296679	Library #3
**AAVDJYF**	GTGCTTGTCATGCCG	0.5324835874	Library #3
**AAVJEA2-D1**	GGTTGACAGTGGGCT	0.9752912245	Library #3

**AAVJEA2-D10**	GTGCGCAGGTTAGTG	0.3474290406	Library #3
**AAVJEA2-D16**	TATAACTTAGCTGAT	0.2695744561	Library #3
**AAVJEA2-D3**	CTTCTTCAGGCAACC	0.2289951097	Library #3
**AAVJEA2-D7**	ACCAACCGGTGTGGG	0.2463396393	Library #3
**AAVJEA2-H1**	GCAATTATCATAGTC	1.7229416159	Library #3
**AAVJEA2-H11**	CCACTAGGATCCGGA	0.186873846	Library #3
**AAVJEA2-H17**	ATCTCGAAGCGCGTA	0.1896395071	Library #3
**AAVJEA2-H2**	TTCATCGGCCGCTAA	0.411610327	Library #3
**AAVJEA2-H8**	CGTCCTGTAAGGAGT	1.2539458601	Library #3
**AAVJEA2-S11**	CAAGGCTTTCTGATC	0.2213099982	Library #3
**AAVJEA3-D12**	TTGGCAGAGGATCAC	0.4518062337	Library #3
**AAVJEA3-D16**	TCGGCTCTGTTCTAG	0.5226854782	Library #3
**AAVJEA3-D2**	TACGTATCGCGTGAT	0.3017181282	Library #3
**AAVJEA3-D20**	TTTAGGCGCGGCTTG	0.2806615755	Library #3
**AAVJEA3-D4**	TCGGCGTGGCGGTCG	0.1824438932	Library #3
**AAVJEA3-D5**	GGTACAGGACGCAGG	0.2046833983	Library #3
**AAVJEA3-D7**	CTAGGCAGGACACCG	0.3269435684	Library #3
**AAVJEA3-H13**	TACATTTAACTGAAG	0.5045577524	Library #3
**AAVJEA3-H15**	CTCGCGGCCTGAGGG	1.2482921635	Library #3
**AAVJEA3-H19**	CTAGATAAATGCGGT	0.4140822896	Library #3
**AAVJEA3-H20**	ACCTGAGTTTGGTGG	0.7553844526	Library #3
**AAVJEA3-H3**	TAGAGTATGAGTGGT	0.2878408728	Library #3
**AAVJEA3-H4**	GAGCGGGCAGACGAT	4.6929271766	Library #3
**AAVJEA3-H5**	GACTTTGACATGTCA	0.1530169325	Library #3
**AAVJEA3-S1**	CCGTCGAAGAAGGGA	0.5183860581	Library #3
**AAVJEA3-S10**	CATGCCATGTGTATC	2.3684665029	Library #3
**AAVJEA3-S2**	CGTCCGTCTAATGAA	0.274118625	Library #3
**AAVJEA3-S4**	GGCAGCGGACACGTG	0.6733365058	Library #3
**AAVJEA3-S5**	GACCACTTATCGCCA	0.1938573443	Library #3
**AAVJEA3-S7**	ATCCTCTCCGCTACC	1.5773813627	Library #3
**AAVJEA3-S8**	TAGCACCATTTACGG	0.2471554686	Library #3
**AAVLK03**	TGTTTAGGTGAGCCT	0.9184931928	Library #3
**AAVM41**	GAGGTCCAGAGGAAG	0.5447536591	Library #3
**AAVpo1_A1**	TTGGAACGTGGGCTT	0.5666749908	Library #3

**AAVpo1_A2**	AGATTCAAAGCTGCG	0.4246717531	Library #3
**AAVpo1_A6**	TGTTGGAAGGTATCA	0.3657851985	Library #3
**AAVpo1_P4**	GTTGTGCCCTGAGTG	0.4853286572	Library #3
**AAVpo1_P5**	ACCGTATCTCTCCGG	0.5556449795	Library #3
**AAVpo1_WT**	TGGTTTACAAATTAT	1.2975356165	Library #3
**AAVrh10_A1**	CTACCTATTTACTCT	0.8566614946	Library #3
**AAVrh10_A2**	ACCGGGCGTTGAGGC	0.17443245	Library #3
**AAVrh10_A6**	ACTGTGATGGGTTAG	0.8216624201	Library #3
**AAVrh10_P2**	GACTTGGTTGTGACG	0.4624283304	Library #3
**AAVrh10_P4**	TTGTTGTATGAGCAG	0.3186384267	Library #3
**AAVrh10_P5**	TCCACGGAGGCTGCG	1.1113307505	Library #3
**AAVrh10_WT**	GGTCTTTGCTCGGTG	0.4966686837	Library #3

Supplemental Figures

a

FACS-sorting strategy to isolate qNSCs; aNSCs; TAPs and neuroblasts

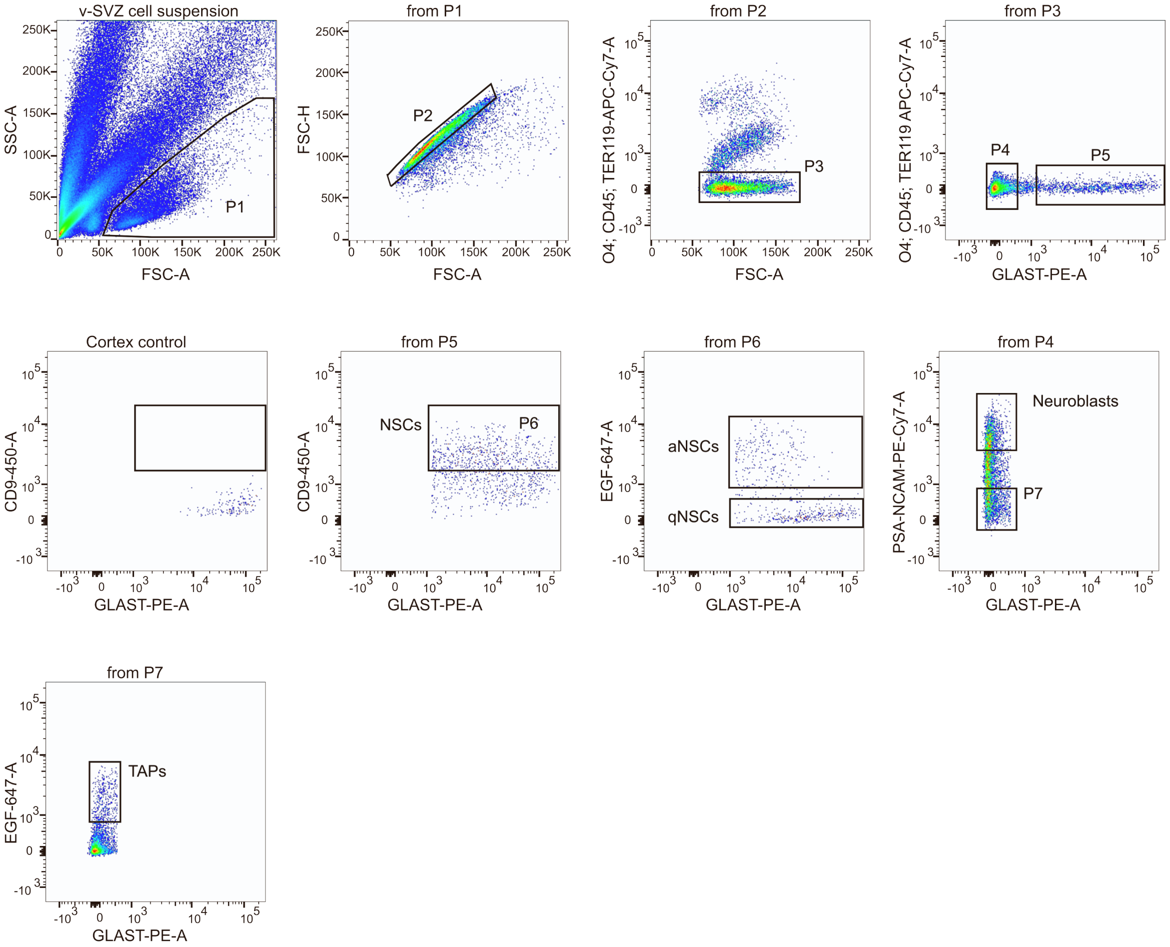

b

FACS-sorting strategy to isolate oligodendrocytes; astrocytes and ependymal cells

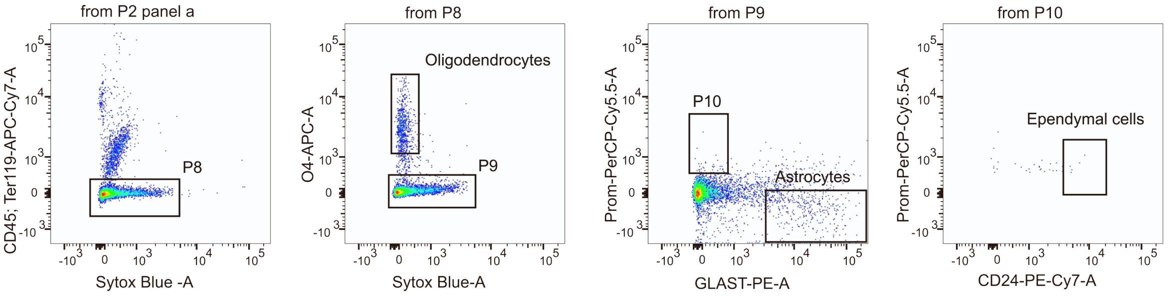

Figure S1, related to Figure 1:

a FACS sorting strategy to isolate total NSCs, qNSCs, aNSCs, TAPs and neuroblasts. **b** FACS sorting strategy to isolate oligodendrocytes, astrocytes and ependymal cells.

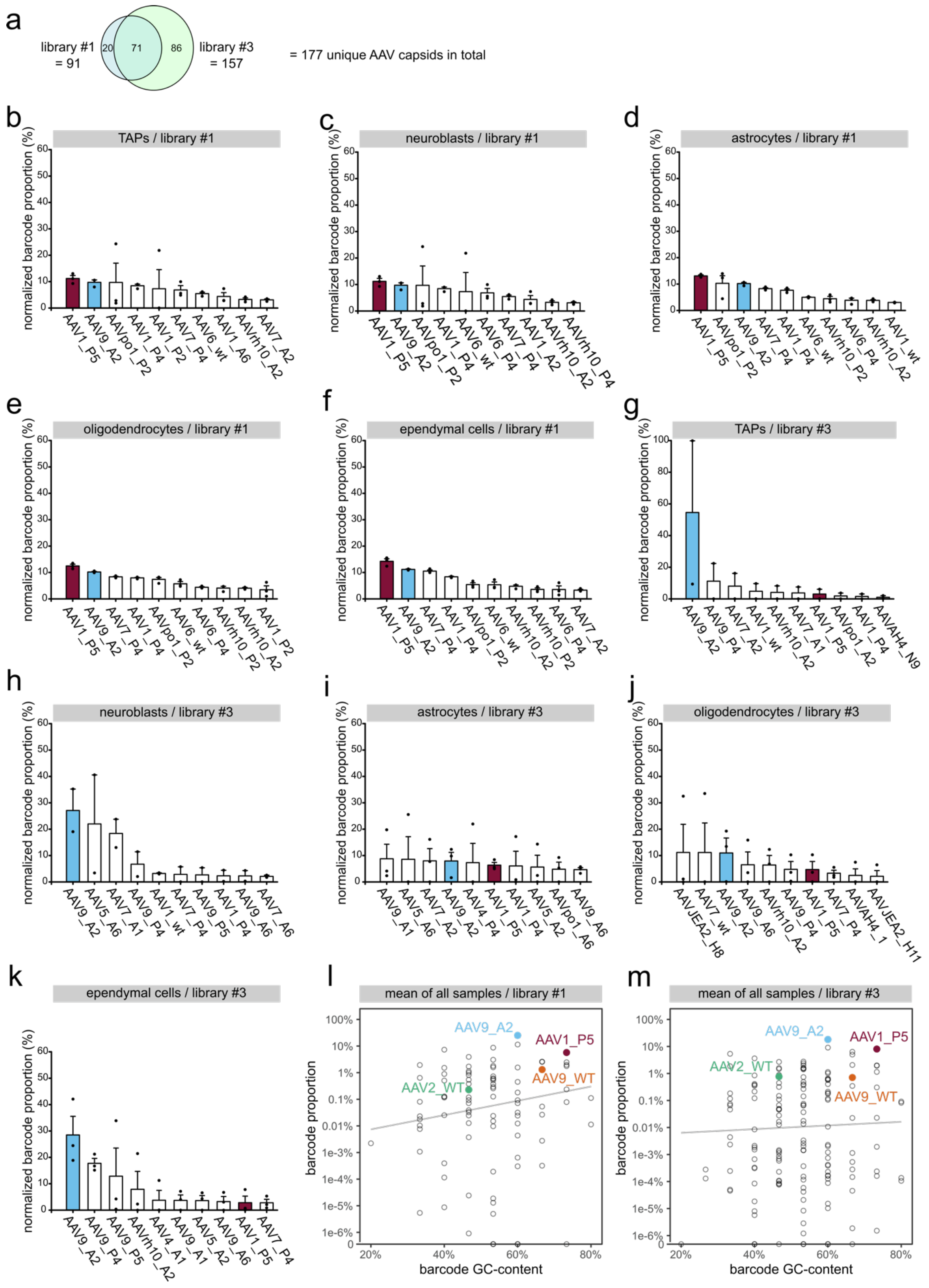

Figure S2, related to Figure 1:

a Number of barcoded AAV capsids in library #1 and library #3. 71 capsids are contained in both libraries. **b-f** Normalized barcode proportion over different FACS-sorted cell types seven days after library #1 transduction of **b** TAPs, **c** neuroblasts, **d** astrocytes, **e** oligodendrocytes and **f** ependymal cells. **g-k** Normalized barcode proportion over different FACS-sorted cell types seven days after library #3 transduction of **g** TAPs; n=2 sets, **h** neuroblasts; n=2 sets, **i** astrocytes, **j** oligodendrocytes and **k** ependymal cells. **l,m** Correlation of barcode GC-content and mean barcode proportion across all samples of library #1 (**l**; Spearman's ρ=0.10; p=0.33) and library #3 (**m**; Spearman's ρ=0.07; p=0.36). All mice were eight weeks old at the time of AAV injection, and all values are given as mean ± SEM; n=3 sets unless stated otherwise.

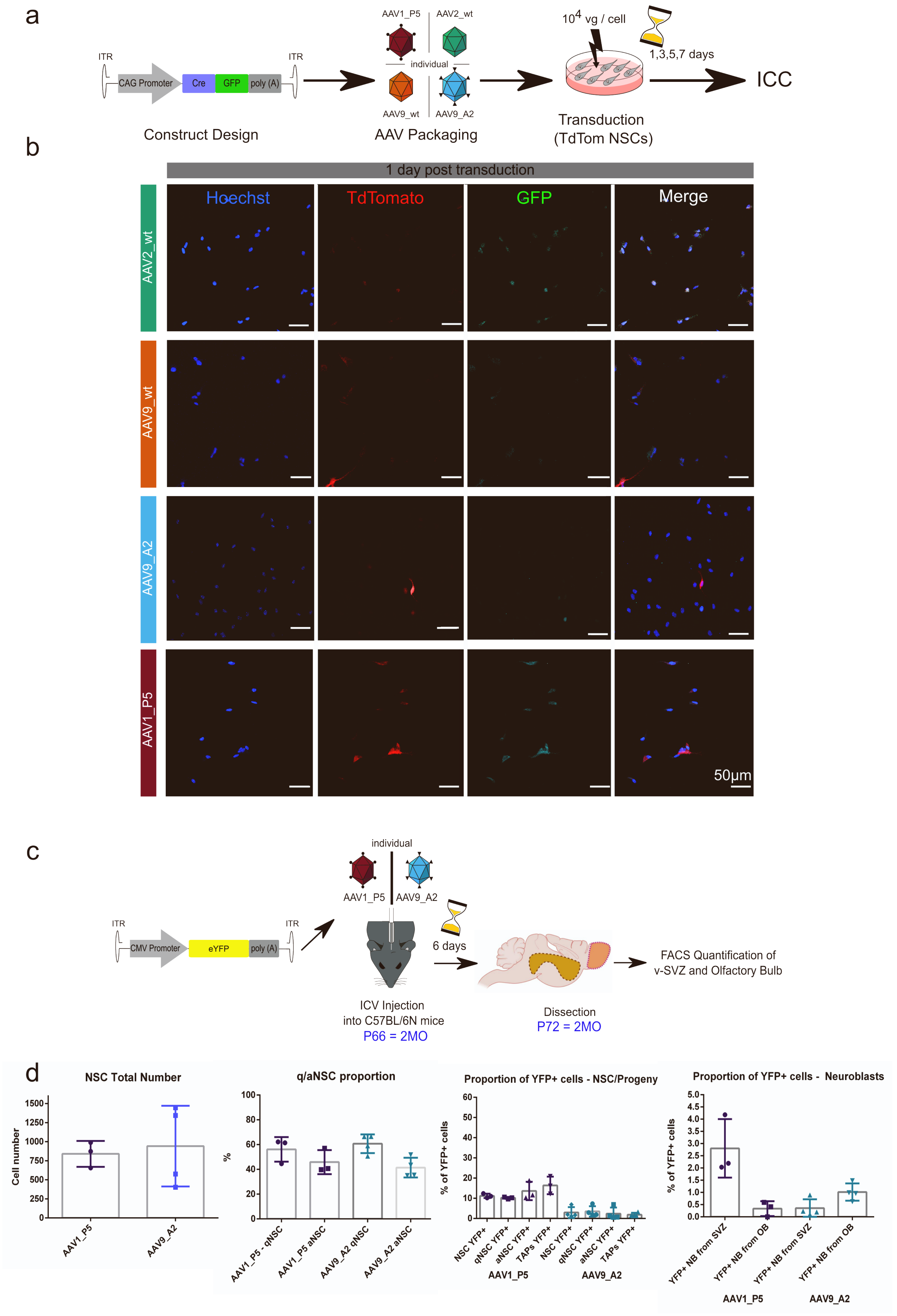

Figure S3, related to Figure 2

a Schematic illustration of the experimental outline to *in vitro* validate different AAV capsids. **b** Representative images of NSCs *in vitro* transduced with different AAV capsids at day 1 after transduction; scale bar 50 µm. **c** Schematic illustration of the experimental outline to perform labeling efficiency analysis of the SVZ and olfactory bulb by FACS Quantification using either AAV1_P5_eYFP or AAV9_A2_eYFP (10^{10}vg/mouse). **d** Quantification of total NSC number in the v-SVZ; proportion of quiescent to active NSCs; labeling efficiency of NSC and TAPs in the v-SVZ; and labeling efficiency of neuroblasts in the v-SVZ and olfactory bulb. The overall NSC labeling efficiency was higher with AAV1_P5 (11.19% ± 0.63, n=3) compared to AAV9_A2 (2.96 ± 1.34, n=4) ($p<0.01$, two-sided Student's t-test). All values are given as mean ± SEM.

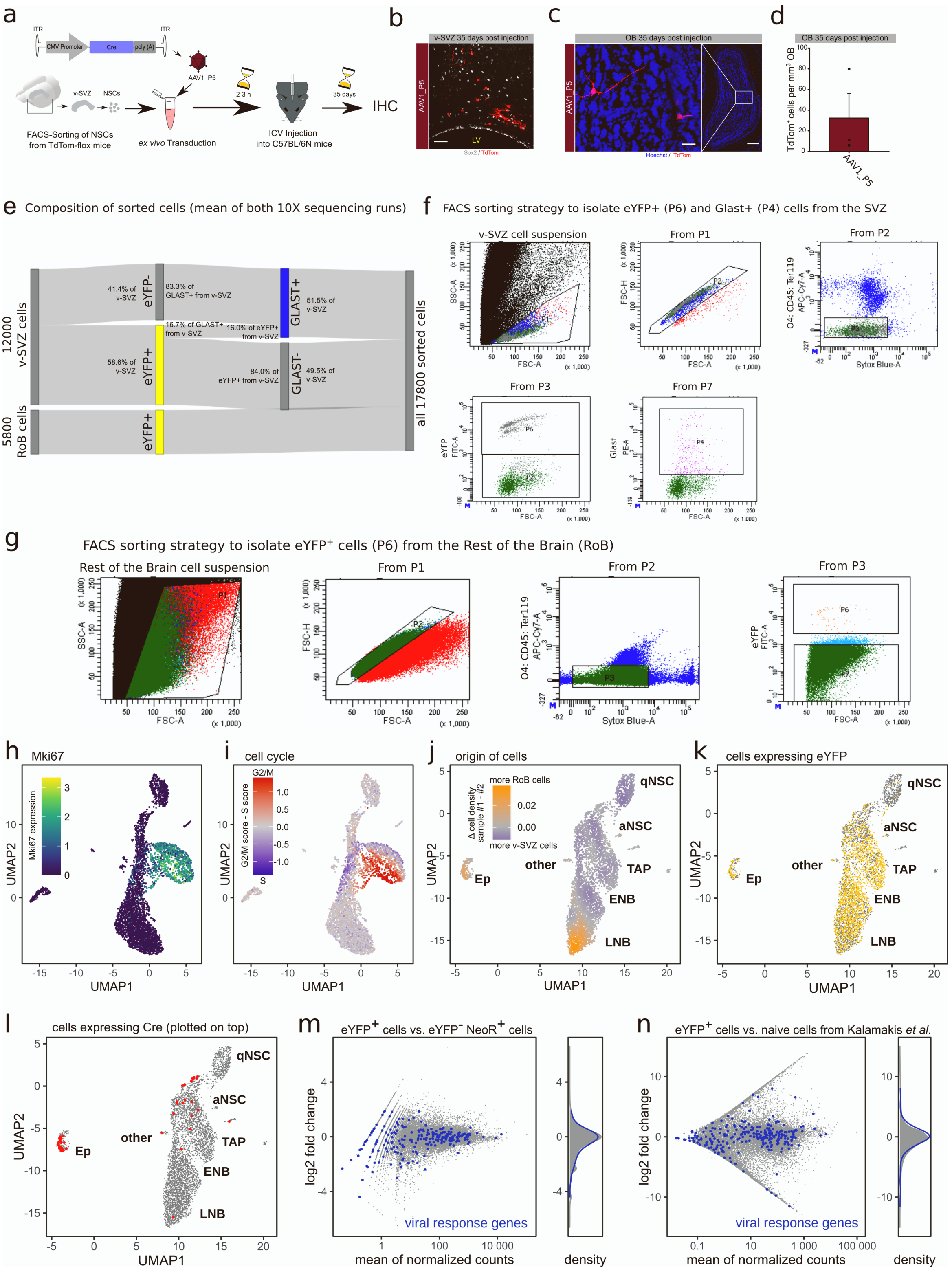

Figure S4, related to Figure 3

a Schematic illustration of the experimental outline to perform *ex vivo* manipulation and transplantation of NSCs. **b** IHC of the v-SVZ (scale bar 50 µm) and **c** Olfactory bulb (OB) neurons (scale bar 200 µm and 30 µm). **d** Quantification of tdTomato-positive cells in the OB; n=3. For this part, all mice were eight weeks old at the time of stereotactic injection of AAVs. **e** Composition of cells that were sorted for scRNA-seq. Cells are grouped according to their tissue of origin (v-SVZ or RoB) and their surface marker (eYFP and/or GLAST) expression. Shown is the average of both single-cell RNA sequencing runs. **f** FACS sorting strategy to isolate $eYFP^+$ and also $eYFP^-/GLAST^+$ cells from the v-SVZ. **g** FACS sorting strategy to isolate $eYFP^+$ cells from the RoB. **h-l** 2D representation of single-cell transcriptomes before regressing out the effects of cell cycle heterogeneity (h,i) and after (j-l). **h** Cells expressing Mki67 (proliferation marker protein Ki-67, log-normalized UMI counts) form a distinct group. **i** Cell cycle phase scores highlight cells expressing canonical markers of S phase (blue) and G2/M phase (red). **j** Putative RoB cells are located at the end of the NSC lineage. Cell color indicates whether nearby cells mostly stem from sample #1 or sample #2 (see Methods for details). Sample #1 contains more cells from RoB, hence orange cells in the main lineage are mostly from RoB. **k,l** Cells with at least one eYFP (**k**) or Cre (**l**) transcript are highlighted in yellow or red. **m,n** MA plots of gene expression differences between $eYFP^+$ cells and $eYFP^-$ $NeoR^+$ cells (**m**) or $eYFP^+$ cells and untransduced cells from[2] (**n**). Right: $\log_2$ fold change distribution for all genes (gray) and viral response genes (blue). RoB, rest of the brain (entails the striatum, rostral migratory stream and olfactory bulb).

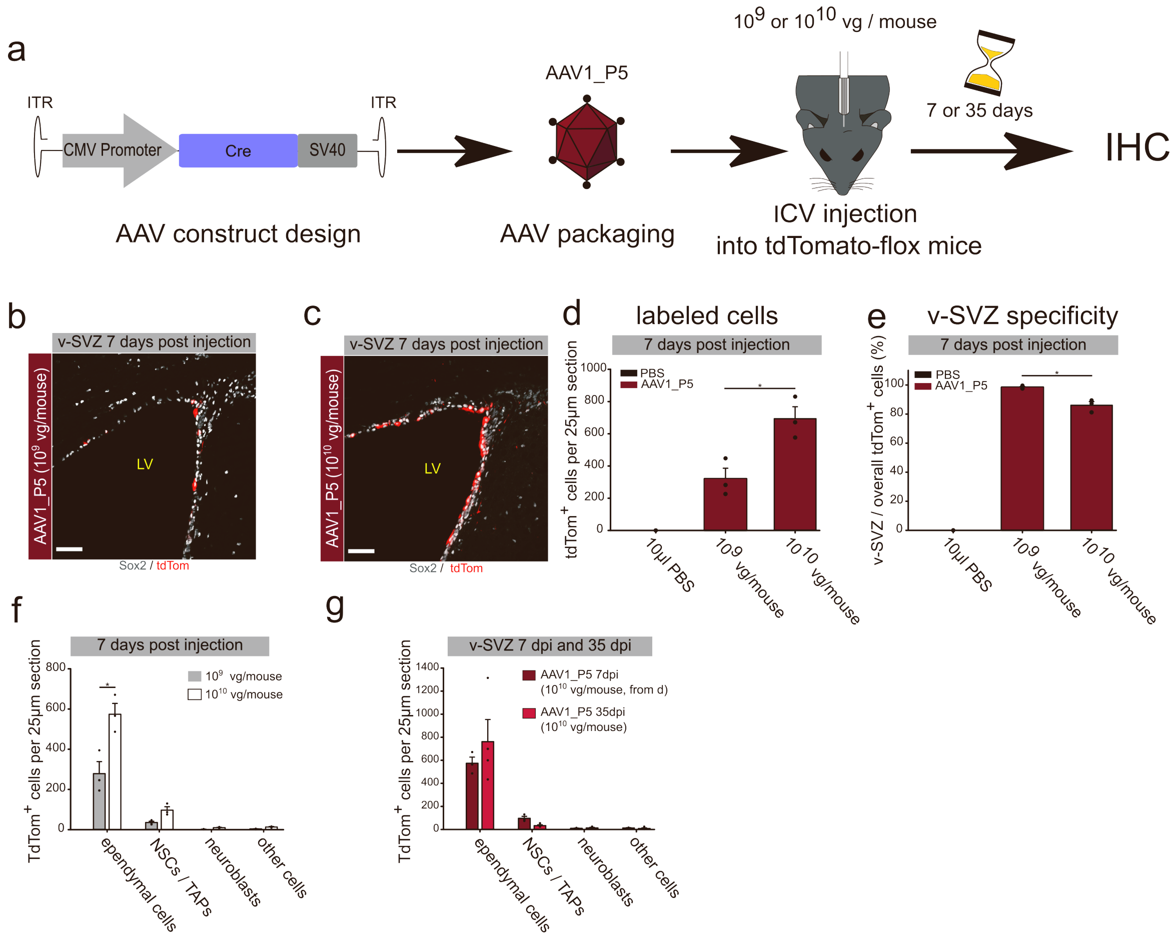

Figure S5 related to Figure 4

a Schematic illustration of the experimental outline to test v-SVZ labeling with different AAV concentrations. **b,c** IHC of the v-SVZ after injecting **b** 10^9 or **c** 10^{10} vg per mouse (scale bar 50 µm). **d** Quantification of the total number of tdTomato-labeled cells within the v-SVZ injected with 10^9 vg per mouse (319.89 ± 66.2) vs. 10^{10} vg per mouse (694 ± 73.92). **e** Quantification of tdTomato-labeled cells located in the v-SVZ among all tdTomato-positive cells in a 25µm thick coronal brain section (10^9 vg/mouse (98.4% ± 0.612) vs. 10^{10} vg/mouse (86.0 % ± 2.51)). **f** Quantification of ependymal cells (10^9 vg per mouse (278.67 ± 59.82) vs. 10^{10} vg per mouse (574.22 ± 53.74)), NSCs, neuroblasts and other cells in the v-SVZ. **g** Quantification of the total number of tdTomato-labeled cells within the v-SVZ of mice at 35dpi; n=4. All mice were eight weeks old at the time of AAV injection; n=3. All values are given as mean ± SEM; $**p \leq 0.01$

and ***$p \leq 0.001$ (Student's t-test). Cre, Cre recombinase; SV40, Simian-Virus 40 polyA signal; ICV, Intracerebroventricular; IHC, immunohistochemistry.

Supplementary Figure S6 related to Figure 4

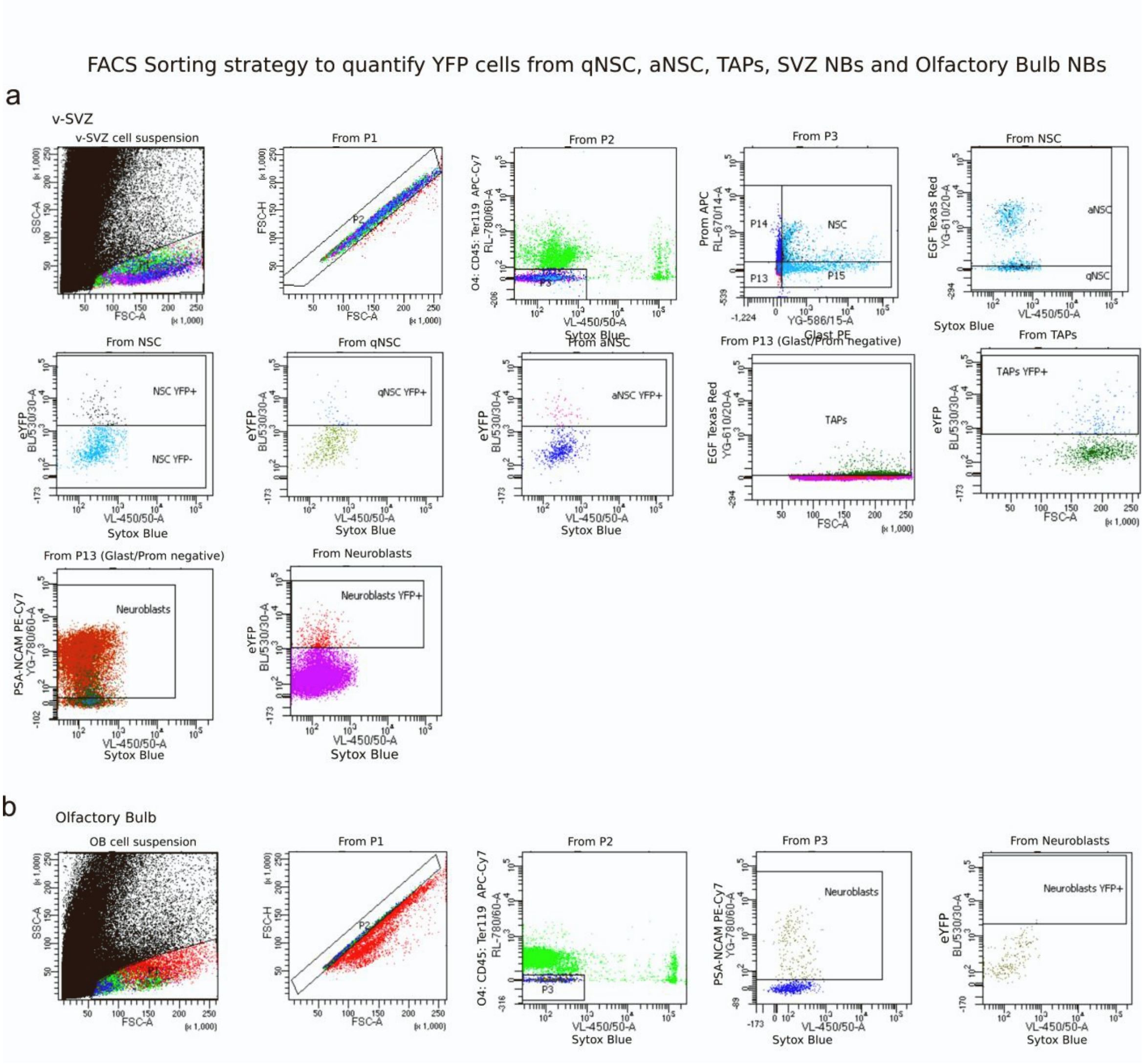

a FACS sorting strategy to quantify the percentage of YFP$^+$ cells from qNSCs, aNSCs, TAPs and NBs in the v-SVZ (related to Fig S3c-d and 4f-g). **b** FACS sorting strategy to quantify YFP$^+$ cells from NBs in the olfactory bulb.
