# Supplementary material for: High throughput screening of novel AAV capsids identifies variants for transduction of adult NSCs within the subventricular zone
Source: Mol Ther Methods Clin Dev. 2021 Jul 16;23:33–50. doi: 10.1016/j.omtm.2021.07.001 (PMC8427210; doi:10.1016/j.omtm.2021.07.001)
Supplement: Document S2. Article plus supplemental information [file mmc2.pdf]

Molecular Therapy
Methods & Clinical Development
Original Article

High throughput screening of novel AAV capsids identifies variants for transduction of adult NSCs within the subventricular zone

Lukas P.M. Kremer,[1,5,6] Santiago Cerrizuela,[1,6] Sascha Dehler,[1,7] Thomas Stiehl,[4,7] Jonas Weinmann,[2] Heike Abendroth,[1] Susanne Kleber,[1] Alexander Laure,[1] Jihad El Andari,[2] Simon Anders,[5] Anna Marciniak-Czochra,[4] Dirk Grimm,[2,3] and Ana Martin-Villalba[1]

[1]Molecular Neurobiology, German Cancer Research Center (DKFZ), 69120 Heidelberg, Germany; [2]Virus-Host Interaction Group, Department of Infectious Diseases/Virology, Heidelberg University Hospital, Cluster of Excellence Cell Networks, BioQuant, 69120 Heidelberg, Germany; [3]German Center for Infection Research (DZIF) and German Center for Cardiovascular Research (DZHK), partner site Heidelberg, 69120 Heidelberg, Germany; [4]Institute of Applied Mathematics, Interdisciplinary Center for Scientific Computing and BioQuant, Heidelberg University, 69120 Heidelberg, Germany; [5]Center for Molecular Biology of Heidelberg University (ZMBH), 69120 Heidelberg, Germany

The adult mammalian brain entails a reservoir of neural stem cells (NSCs) generating glial cells and neurons. However, NSCs become increasingly quiescent with age, which hampers their regenerative capacity. New means are therefore required to genetically modify adult NSCs for re-enabling endogenous brain repair. Recombinant adeno-associated viruses (AAVs) are ideal gene-therapy vectors due to an excellent safety profile and high transduction efficiency. We thus conducted a high-throughput screening of 177 intraventricularly injected barcoded AAV variants profiled by RNA sequencing. Quantification of barcoded AAV mRNAs identified two synthetic capsids, peptide-modified derivative of wild-type AAV9 (AAV9_A2) and peptide-modified derivative of wild-type AAV1 (AAV1_P5), both of which transduce active and quiescent NSCs. Further optimization of AAV1_P5 by judicious selection of the promoter and dose of injected viral genomes enabled labeling of 30%–60% of the NSC compartment, which was validated by fluorescence-activated cell sorting (FACS) analyses and single-cell RNA sequencing. Importantly, transduced NSCs readily produced neurons. The present study identifies AAV variants with a high regional tropism toward the ventricular-subventricular zone (v-SVZ) with high efficiency in targeting adult NSCs, thereby paving the way for preclinical testing of regenerative gene therapy.

INTRODUCTION

The adult brain has long been considered a tissue with no regenerative capacity, partly due to the absence of pluripotent cells. In the late 1990s, a reservoir of neural stem cells (NSCs) with the potential to generate glia and neuronal progeny was identified in the adult mammalian brain.[1,2] The largest reservoir of NSCs in rodents is located along the walls of the lateral ventricles, the so-called ventricular-subventricular zone (v-SVZ). The potential of these NSCs to produce different glia and neuronal subtypes has been demonstrated by lineage-tracing studies.[3–5] NSCs get activated to provide progeny for tissue homeostasis but also in the frame of a traumatic brain injury.[6–11] However, the ability to activate NSCs highly declines with age,[12] hampering repair of the brain. This fairly limited endogenous-regenerative capacity calls for new strategies to specifically target and genetically modify adult NSCs within the natural environment of the brain.

Many different viral and transgenic approaches have been developed in the past to manipulate adult NSCs and their progeny.[13] For a long time, onco-retroviruses and lentiviruses that integrate their genomes into the host cellular chromatin were the tools of choice. However, limitations of integrating viruses,[14] such as insertional mutagenesis,[15,16] gradual silencing of the inserted transgene,[17,18] and the fact that not all non-dividing cells are equally transduced *in vivo*,[19] hamper their use for targeting of especially quiescent (q)NSCs within the v-SVZ. Over the last few years, the non-enveloped adeno-associated viral (AAV) vectors have taken center stage as a gene-delivery vehicle for human gene therapy with two gene therapeutic approaches that have gained regulatory approval for commercial use in patients: Glybera (uniQure) and Luxturna (Novartis), and with a large amount of AAV gene therapeutic strategies even in the CNS under clinical development, as reviewed in Hocquemiller et al.,[20] Deverman et al.,[21] Foust et al.,[22] and Wang et al.[23]

AAVs are small virus particles, belonging to the dependoviruses within the parvoviridae family with a capsid diameter of ~22 nm that is sterically limiting its genome to ~4.7 kb.[24] The original AAV genome consists of only two genes, the *rep* and *cap* gene, which are organized in three open reading frames. The *cap* gene determines

Received 5 February 2021; accepted 9 July 2021;
https://doi.org/10.1016/j.omtm.2021.07.001.

[6]These authors contributed equally

[7]These authors contributed equally

Correspondence: Ana Martin-Villalba, Molecular Neurobiology, German Cancer Research Center (DKFZ), 69120 Heidelberg, Germany.
E-mail: a.martin-villalba@dkfz.de

This is an open access article under the CC BY license (http://creativecommons.org/licenses/by/4.0/).

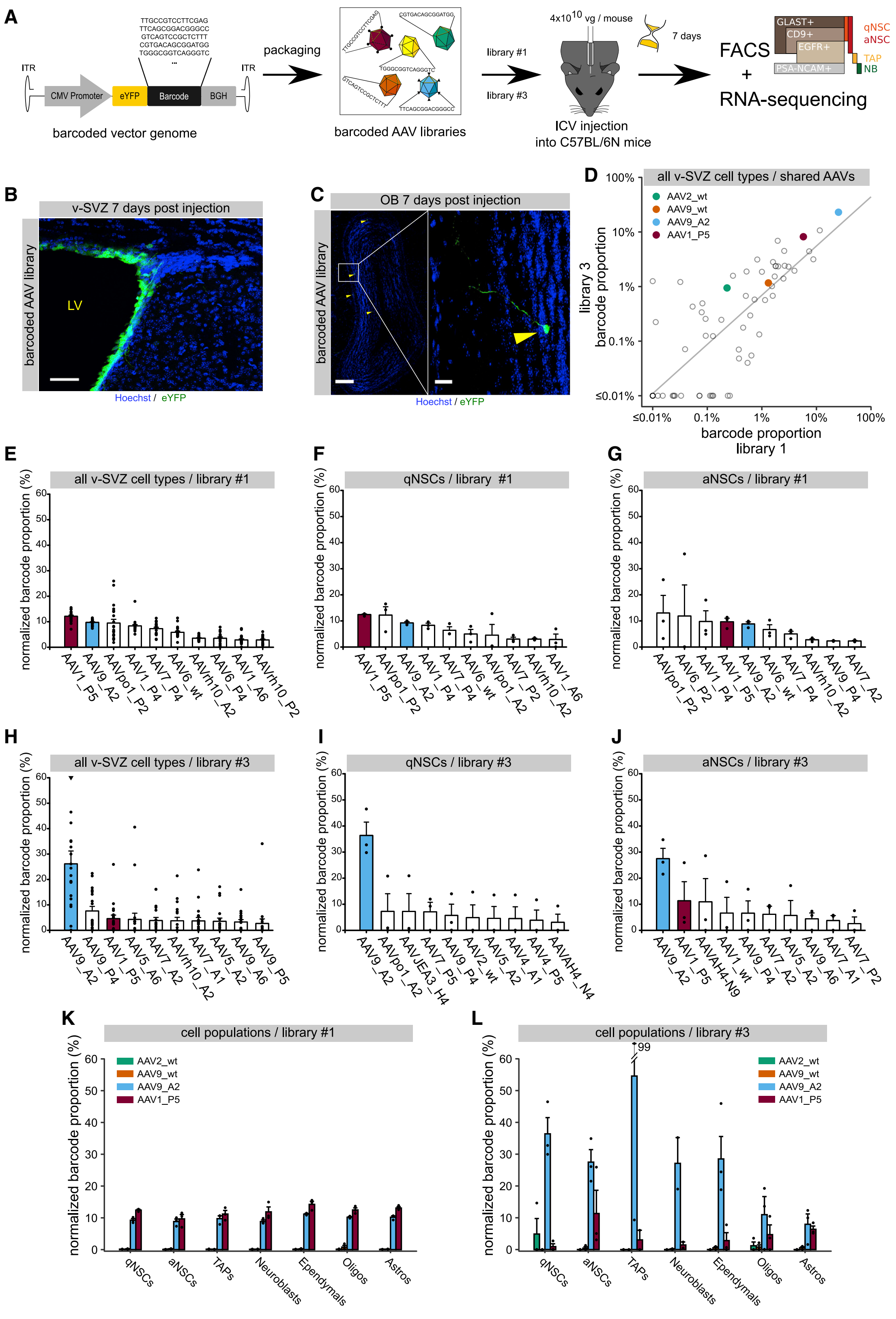

(legend on next page)

the structure of the AAV capsid, whereas the *rep* gene is involved in several processes ranging from transcription initiation to packaging of the AAV genome. For vector production, these genes are commonly delivered in *trans* and thus can be easily modified.[25–33] Over the last decades, hundreds of AAV isolates were identified in various species, with an interestingly high homology regarding their capsid protein amino acid sequences, e.g., up to 99% for the primate isolate AAV1 compared to the human isolate AAV6.[34] Favorable safety profiles combined with the ability to mediate long-term transgene expression and to efficiently target many different human tissues are major assets that make AAVs a preferred technology.[25,35–38]

Nonetheless, specific targeting of NSCs in the v-SVZ has remained challenging to date. Whereas the most efficient wild-type (WT) serotype, AAV9, shows high transduction efficiency upon intravenous and intracranial injection, it mainly targets neurons and astrocytes, but not NSCs.[22,39–41] Just recently, the power of structure-guided DNA shuffling was used to develop the newly engineered AAV variant SCH9. This new variant was able to target cells in the v-SVZ including NSCs.[42] However, to date, the usefulness of AAV vectors for transduction of stem cells remains debated, mainly based on conflicting reports concerning their transduction efficiency as reviewed.[43] The variable regions of the viral protein (VP), which is encoded by the *cap* gene, are involved in receptor binding and antibody recognition and thus modifications thereof can be used to guide targeting of specific cell types. Engineering of the AAV capsid for optimization of organ, region, or cell specificity can be achieved by methods such as random *cap* gene mutation, DNA family shuffling, or peptide display, combined with *in vivo* selection.[42,44–50] Most recently, barcoding of double-stranded encapsidated DNA and next-generation sequencing (NGS) were shown to allow for high-throughput screening of AAV capsid libraries.[51,52] Taking these advances as a platform, we apply here these barcoded AAV libraries by intracerebroventricular injection of the adult rodent brain in order to find an optimal candidate to transduce NSCs from the v-SVZ. By using a combination of NGS, immunohistochemistry (IHC), flow cytometry, and mathematical modeling, we validate transduction of the NSCs within the v-SVZ and their neurogenic lineage by the novel AAV capsid peptide-modified derivative of WT AAV1 (AAV1_P5).

RESULTS

To identify AAV capsids able to transduce NSCs in the v-SVZ with the highest transduction efficiency possible, we performed an NGS-based high-throughput screening of 177 different barcoded AAV capsid variants. These AAV variants comprise 12 AAV WTs, 94 newly generated peptide display mutants based on these WTs, and 71 chimeric capsids generated through DNA family shuffling. Among the synthetic capsids are 24 previously published benchmarks, with the remaining ones being generated as described in Materials and methods, in Table S4, and in greater detail in Weinmann et al.[53] To assess the performance of individual AAVs, the capsid variants were uniquely barcoded with a 15-nucleotide (nt)-long random DNA sequence and packaged into an AAV vector expressing a cytomegalovirus (CMV) promoter-controlled eYFP (enhanced yellow fluorescent protein) that harbors the barcode in its 3′ untranslated region (UTR). A library comprising either 91 (library #1 from Weinmann et al.[53]) or 157 (library #3 from Weinmann et al.[53]) capsid variants was directly injected into the lateral ventricles of the adult mouse brain (10^{10} viral genomes [vgs] in 2 μL per mouse) (Figures 1A and S2A).

7 days post-injection (dpi), qNSCs and active NSCs (aNSCs), as well as other cell populations of the v-SVZ, including transient amplifying progenitors (TAPs), neuroblasts, astrocytes, oligodendrocytes, and ependymal cells, were fluorescence-activated cell sorting (FACS) analyzed as previously described[6,12,54] (Figures S1A and S1B; Tables S2 and S3). Finally, RNA libraries from the different cell populations were generated for NGS analysis (Figure 1A). In parallel, additional mice were sacrificed at 7 dpi for detection of the eYFP reporter in the v-SVZ. Efficient transduction of cells in the v-SVZ by both AAV libraries was confirmed by detecting the expression of the eYFP reporter along the ventricular walls (Figure 1B). Already after 7 dpi, few eYFP-positive ($eYFP^+$) cells migrated to the olfactory bulb (OB) and were detected in the core and granular cell layer (GCL; Figure 1C), indicating that the AAV vector was retained along the lineage and did not prevent migration.

For AAV mRNA analysis, capsids were ranked within each sorted cell population by the relative expression of their cognate barcodes, normalized by their frequency within library #1 and library #3. Overall capsid rankings of the 71 capsids shared by both libraries revealed the same top candidates and correlated strongly (Spearman's rank correlation $\rho = 0.84$, $p < 0.01$) (Figure 1D). Furthermore, we did not find a significant association between barcode guanine-cytosine (GC) content and frequency in either library (Figures S2L and S2M and Materials and methods), indicating that the

Figure 1. *In vivo* screening to identify AAV capsids that specifically target the v-SVZ

(A) Schematic illustration of the experimental outline to perform the *in vivo* screening, including markers used to sort cells of the NSC lineage (see Figure S1 for sorting strategy). IHC of (B) the v-SVZ (scale bar, 50 μm) or (C) the olfactory bulb (OB; scale bars, 200 μm and 30 μm) after injection of library #1 into the lateral ventricle. (D) Mean barcode proportion over all FACS cell types for libraries #1 and #3. Only the 71 capsids shared between the two libraries are shown. (E) Barcode proportion in sample, adjusted for abundance in library (normalized barcode proportion) over all FACS cell types 7 days after library #1 transduction; n = 3 sets per cell type. (F and G) Normalized barcode read count 7 days after library #1 transduction of (F) quiescent NSCs (qNSCs) or of (G) aNSCs; n = 3 sets. (H) Normalized barcode read count over all FACS cell types 7 days after library #3 transduction; n = 2 sets for TAPs and neuroblasts; for all other cell types, n = 3 sets per cell type. (I and J) Normalized barcode read count 7 days after library #3 transduction of (I) qNSCs or of (J) aNSCs; n = 3 sets. (K and L) Normalized barcode read count of AAV2_WT, AAV9_WT, AAV9_A2, and AAV1_P5 after library #1 (K) and #3 (L) transduction of qNSCs, aNSCs, TAPs, neuroblasts, ependymal (Ep) cells, astrocytes, and oligodendrocytes. All mice were 8 weeks old at the time of AAV injection, and all values are given as mean ± SEM. ITR, inverted terminal repeat; BGH, bovine growth hormone poly(A) signal; eYFP, enhanced yellow fluorescent protein; ICV, intracerebroventricular. A set always consists of 6 mice. Three independent experiments were performed resulting in n = 3 sets (3 × 6 mice = 18 mice in total).

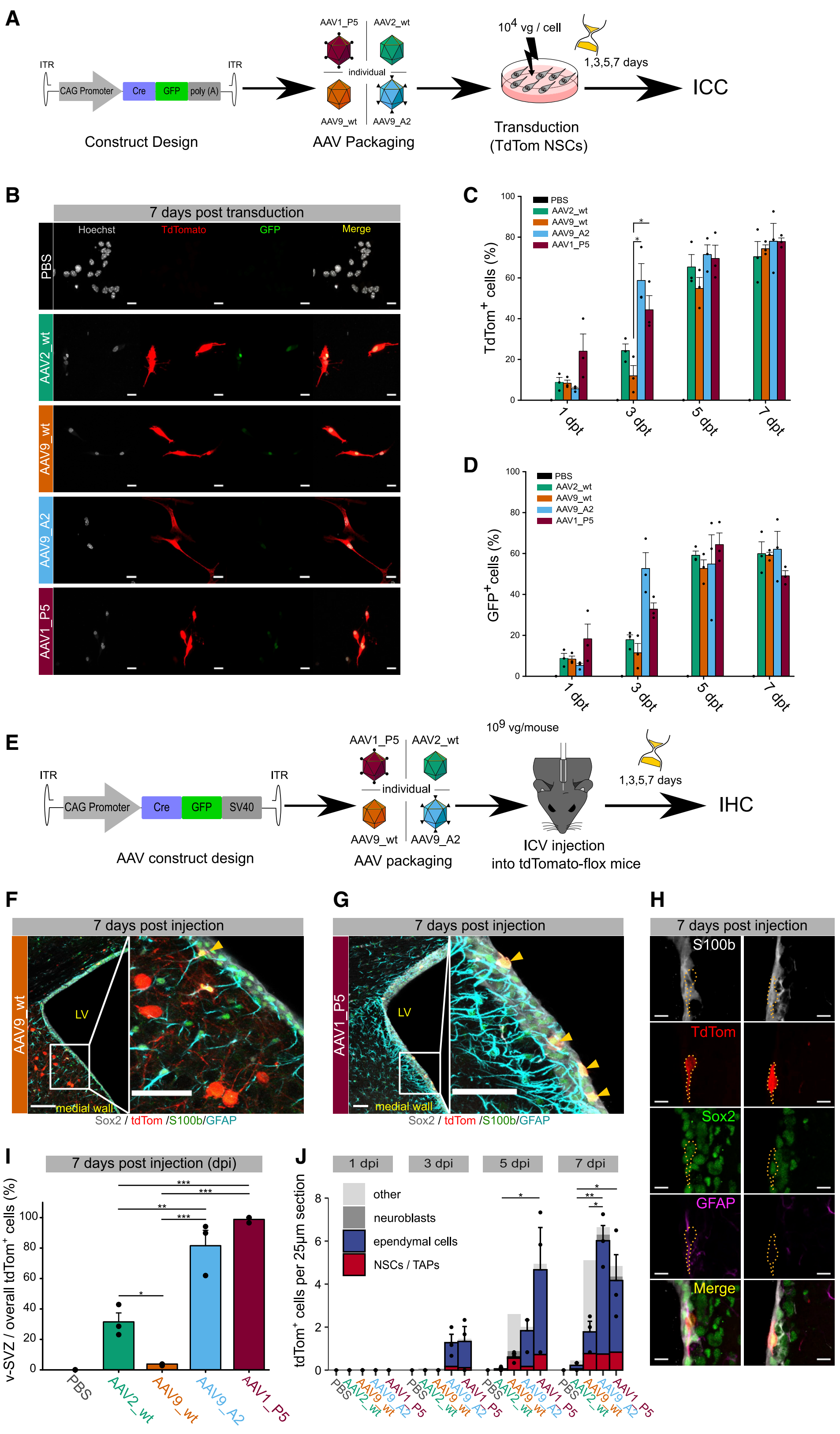

(legend on next page)

results are not strongly influenced by GC bias. Further analysis revealed that two synthetic capsids, AAV1_P5 and AAV9_A2 (peptide-modified derivative of WT AAV9), stood out as the most efficient AAV capsid variants based on the ranking of their barcode enrichment (Figures 1D–1J and S2B–S2K). Notably, both aNSC and qNSC were robustly transduced by these two AAV capsids (Figures 1F, 1G, 1I, and 1J). Besides, AAV1_P5 and AAV9_A2 transduced other v-SVZ cell types, such as TAPs (Figures S2B and S2G), neuroblasts (Figures S2C and S2H), astrocytes (Figures S2D and S2I), oligodendrocytes (Figures S2E and S2J), and ependymal cells (Figures S2F and S2K). These two lead candidates clearly outperformed the well-established AAV2 and AAV9 WT capsids across all v-SVZ-cell populations (Figures 1K and 1L), as well as the parent WT AAV1. Taken together, our study has successfully identified AAV capsids that were highly region specific for the v-SVZ, probably due to their inability to migrate out of this region as reported for the SCH9 variant. These candidates exhibited a higher efficiency in targeting both aNSC and qNSC than established WT AAV variants in the v-SVZ *in vivo*.

One potential application of gene therapy is to genetically modify freshly isolated cells and transplant them back to the donor. Hence, to identify the capsid with the fastest transduction rate of isolated NSCs, we assessed the expression dynamics of WT serotypes AAV2 and AAV9, respectively (AAV2_WT and AAV9_WT), AAV9_A2, and AAV1_P5 in NSCs *in vitro*. To detect viral transduction of targeted cells and their progeny, we took advantage of the recombination of pairs of loxP sites by the Cre recombinase (Cre/loxP) system and engineered the AAVs to express a CMV immediate enhancer/βactin (CAG) promoter-controlled Cre recombinase fused to GFP (CAG_-Cre::GFP). We decided to use the CAG promoter to assess performance of these capsids, since this promoter proved to outperform other promoters for *in utero* electroporation of embryonic neural progenitors.[55] Subsequently, we transduced primary-cultured NSCs from B6-Gt(ROSA)26Sortm14(CAG-tdTomato)Hze (tdTomato-flox [TdTom-flox]) mice with these 4 candidates (Figure 2A). Cre-fused GFP and cytoplasmic tdTomato were detected via immunocytochemistry at days 1, 3, 5, and 7 post-transduction (dpt) (Figures 2A and 2B). Interestingly, whereas all capsids showed a similar number of transduced cells at 7 dpt (Figures 2C and 2D), AAV1_P5 exhibited the fastest transduction kinetics (Figure 2C), already showing labeling at day 1 (Figures S3A and S3B).

Next, we investigated whether the newly identified AAV capsids AAV1_P5 and AAV9_A2 also target v-SVZ cells *in vivo*. To this end, we individually injected 10^9 vgs of AAV9_A2, AAV1_P5, or the well-established AAV9_WT and AAV2_WT, all containing the CAG_Cre::GFP construct, into tdTomato-flox mice (Figure 2E). Notably, at 7 dpi, the tropism toward the v-SVZ highly differed between the tested capsids (Figures 2F and 2G). AAV2_WT and in particular AAV9_WT targeted many cells outside of the v-SVZ, especially in the medial and dorsal wall of the lateral ventricles, whereas the striatum was not targeted (Figures 2F and 2G and data not shown). In contrast to the WT capsids, AAV1_P5 and AAV9_A2 demonstrated a significantly higher tropism toward the v-SVZ (Figure 2I). AAV1_P5 showed the most unique tropism, with 98% of all tdTomato-labeled cells lying along the v-SVZ. In addition, transduction rates of overall cells also differed among the four capsids. AAV1_P5 and AAV9_A2 exhibited the fastest kinetics and most robust rate of transduction, with AAV1_P5 transducing the largest number of cells at 5 dpi as compared to the other capsids (Figure 2J). The overall number of transduced NSCs became similar at 7 dpi for all capsids except AAV2_WT (Figure 2J). Nevertheless, AAV9_WT mostly targeted cells lying outside of the ventricular wall that we clearly identified as neurons based on their morphology. This is in line with previous reports showing a high transduction efficiency of AAV9 for neuronal cells.[22,56] By contrast, AAV1_P5 and AAV9_A2 exhibited a selective tropism for the v-SVZ, mainly targeting NSCs/TAPs ($SOX2^+/GFAP^{+/-}/S100B^-$) as well as ependymal cells ($SOX2^+/S100B^+$; Figures 2H and 2J).

Along the wall of the v-SVZ, ependymal cells are organized in a so-called pinwheel architecture with NSCs in the center.[57] Within these structures, ependymal cells outnumber NSCs, explaining why AAV1_P5 and AAV9_A2 transduce more ependymal cells overall. A recent report using single-cell transcriptomics and fate mapping of ependymal cells demonstrates their inability to generate progeny even after growth factor administration or brain injury.[58] This ensures that progeny labeled with AAV1_P5 or AAV9_A2 stems from NSCs. However, manipulated ependymal cells communicate with neighboring NSCs and might indirectly change the progeny of these NSCs. To address this, strategies to de-target ependymal cells, by using a NSC-specific promoter or a microRNA (miRNA)-regulated viral vector,[59,60] might be of use. The latter would require a screening for ependymal cell-specific miRNAs. Taken together, our data

Figure 2. Assessment of expression dynamics and v-SVZ targeting of the lead candidate AAV capsids

(A) Experimental outline to assess expression dynamics of AAV1_P5, AAV9_A2, and two WT capsids *in vitro*. (B) Representative images of NSCs *in vitro* transduced with different AAV capsids 7 days after injection (days post-injection [dpi]); scale bars, 20 μm. (C) Dynamics of tdTomato expression at different time points in primary-cultured NSCs. AAV9_WT_3 dpt (11.9% ± 5.04%) versus AAV9_A2_3 dpt (58.8% ± 8.24%) versus AAV1_P5_3 dpt (44.4% ± 6.94%) (Kruskal-Wallis test followed by Dunn's post hoc test). (D) Dynamics of GFP expression at different time points in primary-cultured NSCs. (C and D) Cultured NSCs were used up to passage 7; n = 3 cell cultures from 3 different mice. (E) Schematic illustration of the experimental outline to *in vivo* validate different AAV capsids. (F and G) IHC of the v-SVZ with markers to discriminate the different cell types after (F) AAV9_WT and (G) AAV1_P5 transduction (scale bars, 100 μm and 50 μm, respectively). (H) Markers for IHC used to discriminate the different cell types (NSCs left; Ep cells right; scale bars, 30 μm). (I) Proportion of tdTomato-labeled cells located in the v-SVZ among all tdTomato-positive cells in a 25-μm-thick coronal brain section. A high proportion indicates regional specificity for the v-SVZ. AAV2_WT (31.5% ± 5.9%) versus AAV9_WT (3.84% ± 0.33%) versus AAV9_A2 (81.6% ± 10.1%) versus AAV1_P5 (98.9% ± 1.13%). (J) Dynamics of tdTomato expression at different time points in the full v-SVZ. Bars are partitioned by the mean proportion of cell types across mice. AAV2_WT_5 dpi (0.06 ± 0.06) versus AAV1_P5_5 dpi (4.67 ± 1.96) and AAV2_WT_7 dpi (0.22 ± 0.11) versus AAV9_A2_7 dpi (6.02 ± 0.71) versus AAV1_P5_7 dpi (4.17 ± 1.20).

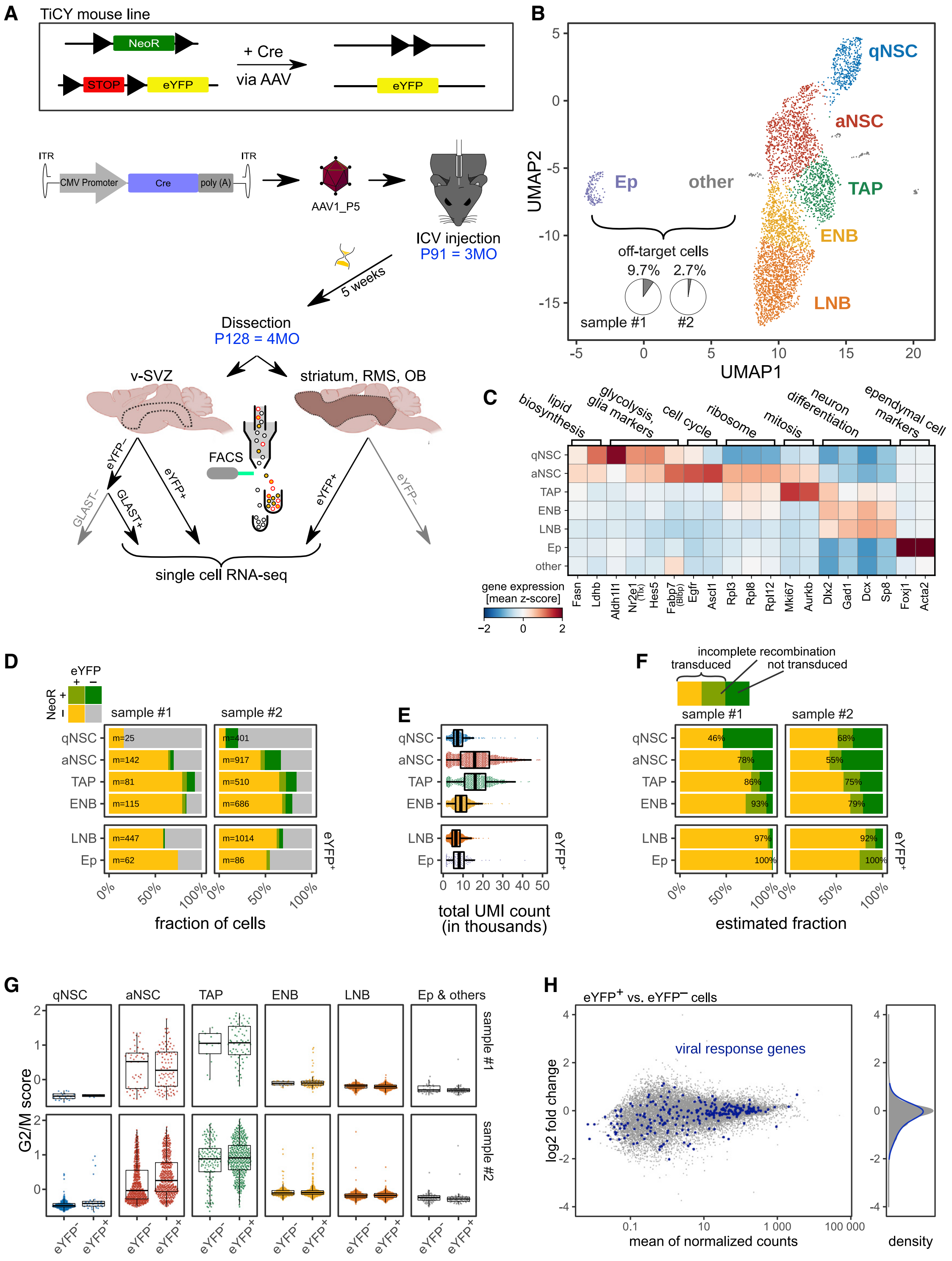

Figure 3. Single-cell RNA sequencing (scRNA-seq) reveals transduction of cells of the adult NSC lineage by AAV1_P5

(A) Experimental outline of labeling, isolation, and scRNA-seq of the adult NSC lineage using the AAV1_P5 capsid. (Top panel) Untransduced cells from the TiCY mouse line express neomycin resistance (NeoR). Cre recombinase (Cre)-mediated recombination induces the expression of eYFP and the loss of NeoR expression. AAV1_P5 loaded with Cre was delivered to the lateral ventricle of P91 TiCY mice. After 5 weeks, all labeled (eYFP$^+$) cells from the v-SVZ and the rest of the brain (striatum, rostral migratory stream [RMS], and OB), as well as further unlabeled NSC lineage cells (GLAST$^+$ from v-SVZ) were sorted and used for scRNA-seq. (B) 2D representation of the resulting 4,572

(legend continued on next page)

demonstrate a unique tropism and fast targeting of NSCs/TAPs and ependymal cells within the v-SVZ by AAV1_P5 and AAV9_A2.

To select the best candidate between AAV1_P5 and AAV9_A2 regarding NSC transduction efficiency, we performed FACS analysis of the v-SVZ and OB of injected mice. 2-month-old C57BL/6N mice were injected with 10^{10} vgs in 10 μL of either AAV1_P5 or AAV9_A2 capsids containing the eYFP reporter under the CMV promoter, as these were the capsids used for the barcoded libraries. 6 dpi, mice were sacrificed, and NSCs with their progeny from the v-SVZ and the OB neuroblasts were analyzed by FACS quantification (Figures S3C, S6A, and S6B). By determining the fraction of YFP$^+$ cells among these cell types, we calculated the labeling efficiency of the different viruses. Our results show that AAV1_P5 has a higher labeling efficiency for NSC (11.19%) than the AAV9_A2 capsid (2.95%) (Figure S3D). This higher transduction efficiency could also be seen for qNSC, aNSC, TAPs, and negative binomials (NBs) from the SVZ (Figure S3D). This prompted us to proceed with the AAV1_P5 capsid for further experiments. Of note, the overall low number of detected YFP$^+$ cells is due to the lower sensitivity of FACS analysis for YFP-expressing cells as compared to mCherry or tdTomato, as previously shown (Tlx -YFP (YFP expression under the *Tlx* [*Nr2e1* nuclear receptor gene] promoter) versus Tlx-tdTomato in Baser et al.[61]). In addition, here, we directly measure the viral YFP as opposed to the measurement of tdTomato expression induced by AAV-Cre in Figure 1.

In order to test the ability of direct AAV1_P5-transduced NSCs to generate progeny, freshly isolated NSCs from tdTomato-flox mice were transduced with AAV1_P5 expressing Cre recombinase under the control of a CMV promoter (CMV_Cre). Thereafter, transduced cells were transplanted into the v-SVZ of C57BL/6N WT mice (Figure S4A). After 35 days, tdTomato-positive neurons were present in the GCL of the OB (Figures S4B–S4D). In summary, transduction of NSCs by AAV1_P5 *ex vivo* does not interfere with their capability to self-renew and differentiate into OB interneurons.

To fully characterize the identity of AAV1_P5-transduced cells in the v-SVZ and the OB, as well as to address potential changes arising from AAV transduction itself, we profiled transduced and untransduced cells from the same mouse by single-cell RNA sequencing (scRNA-seq). To this end, 3-month-old eYFP-reporter mice (B6-Tg [Nr2e1-Cre/ERT2]1Gsc Gt[ROSA]26Sortm1[EYFP]CosFastm1Cgn/Amv [TiCY] and Tlx-CreERT2-YFP mice[62]) were injected with 10^9 vgs/mouse AAV1_P5 harboring the CMV_Cre construct. Upon transduction, Cre recombinase causes the excision of a transcription terminator upstream of eYFP, which leads to eYFP expression. Transduction also causes excision of the neomycin resistance (NeoR) gene (Figure 3A, top). 37 dpi, we isolated cells from the v-SVZ and other brain regions as schematically depicted in Figure 3A. More precisely, we isolated labeled cells of the v-SVZ and the striatum, rostral migratory stream (RMS), and OB, here referred to as rest of the brain (RoB). To capture the remaining unlabeled cells of the NSC lineage in the v-SVZ, we also isolated GLAST$^+$ v-SVZ cells (see Figures 3A and S4E–S4G for the proportion of cell populations). Two samples of two pooled mice each were subjected to scRNA-seq. Initial inspection of the resulting 4,572 single-cell transcriptomes revealed a segregation of proliferating cells as indicated by the expression of the proliferation marker protein KI67 (MKI67) and canonical markers of G2/M and S phase (Figures S4H and S4I). After mitigating the effects of phase heterogeneity by regression, we obtained a continuous trajectory ranging from NSCs to late NBs (LNBs)/immature neurons (Figure 3B). This lineage progression is characterized by downregulation of glia markers, followed by increased expression of ribosomal genes and cell-cycle genes, and finally upregulation of neuron differentiation genes.[6] Visualizing the expression of representative genes from Llorens-Bobadilla et al.[6] recapitulated the same transcriptional progression in our dataset (Figure 3C). Only few eYFP$^+$ off-target cells (sample #1: 9.7%; sample #2: 2.7%) were captured, consisting of mostly ependymal cells (Figure 3B). We found that cells isolated from RoB are located at the very end of this trajectory, as expected (Figure S4J).

Next, we sought to distinguish labeled (eYFP$^+$ NeoR-negative [NeoR$^-$]) cells from unlabeled (eYFP$^-$ NeoR-positive [NeoR$^+$]) cells in our single-cell transcriptomes (Figures 3D and S4K). As expected (Figure 3A, top), eYFP-expressing cells mostly do not express NeoR, and vice versa, cells expressing NeoR mostly do not express *eyfp*. Only very few cells express both *eyfp* and NeoR (samples #1 and #2: 1.4% and 3.7%), possibly due to incomplete Cre-mediated excision. Transcripts of the viral Cre-recombinase, however, were rarely detected and mostly in early stages of the lineage but notably, also in very few cells at the end of the lineage, indicating an overall very low expression that prevents estimation of the dilution of viral transcripts along the lineage (Figure S4L). The floxed genes, *eyfp* and NeoR, exhibited higher expression than the Cre transcript. *eyfp* was more readily detected than NeoR, but ultimately, both genes suffered from the usual "dropout" in scRNA-seq, i.e., the failure to capture and/or detect transcripts.[63] For a substantial fraction of cells, neither NeoR nor *eyfp* was detected. The fraction of such undistinguishable cells was larger in cells with fewer total detected transcripts such as qNSCs and LNBs (Figures 3D and 3E). To overcome this issue and estimate AAV1_P5 transduction efficiency while accounting for

single-cell transcriptomes. Most cells form one continuous trajectory from qNSCs to early NBs (ENBs; mostly from v-SVZ) and late NBs (LNBs)/immature neurons (mostly from rest of brain). Few off-target cells including Ep cells and others (gray) were captured. (C) Mean relative gene expression of NSC lineage markers from Llorens-Bobadilla et al.[6] and Ep cell markers from Shah et al.[58] in each cluster of single cells. (D) Fraction of eYFP$^+$ and NeoR$^+$ single-cell transcriptomes by cell type (m, cells per group). (E) Total number of uniquely identified mRNA molecules (UMI count) per cell, separated by cell type. (F) Maximum likelihood estimate of the fraction of transduced cells, based on values in (C and D). LNB and Ep were sorted by eYFP$^+$ only and act as a control with an expected transduction rate of 100%. (G) Expression of G2/M-phase marker genes across samples and cell types (clusters from B), distinguishing between eYFP$^+$ and eYFP$^-$ cells. (H, left) MA plot of gene-expression differences between eYFP$^+$ and eYFP$^-$ cells. (Right) $\log_2$ fold-change distribution for all genes (gray) and viral response genes (blue).

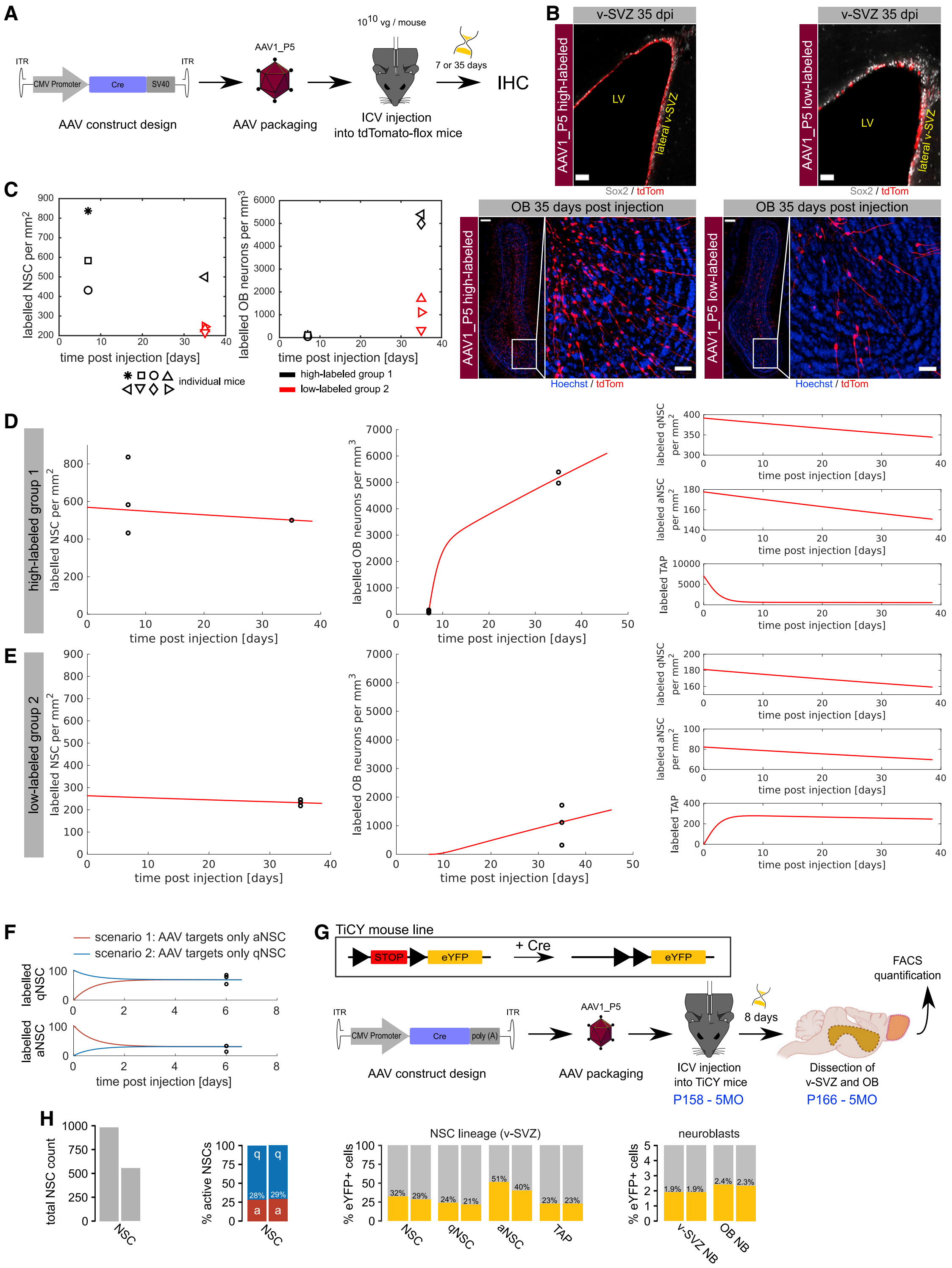

Figure 4. AAV1_P5 targets qNSCs, and the choice of promoter and viral load determines the number of generated OB neurons

(A) Schematic illustration of the experimental outline to test v-SVZ labeling at different time points. (B) IHC of the v-SVZ (scale bars, 50 μm) and OB (scale bars, 200 μm and 50 μm) in the high-labeled and low-labeled group 35 dpi of AAV. (C) Time dynamics of labeled cells. Each mouse is identified by one symbol. Due to the heterogeneity among individual mice, each mouse was assigned to one of two groups. The color of the symbols indicates to which group the respective mouse belongs. (D and E) Comparison of

(legend continued on next page)

total transcript count per cell and the likely different expression strengths of *eyfp* and NeoR, we employed maximum likelihood estimation (Figure 3F and Materials and methods). LNBs (mostly from $eYFP^+$-sorted RoB) and ependymal cells ($GLAST^-$) were used as controls since we know that almost all of these cells are transduced. Overall, we estimated a high transduction efficiency ranging from 46% to 93% for the cell types of the v-SVZ lineage and estimated 92% to 100% transduction in cells used as controls.

Lastly, we assessed whether the transduced cells show transcriptomic differences arising from the viral transduction itself. Both $eYFP^-$ and $eYFP^+$ aNSCs and TAPs showed high expression of commonly used G2/M-phase marker genes (Figure 3G), which suggests that transduction with AAV1_P5 does not affect proliferation. Differential gene-expression analysis between $eYFP^+$ cells and $eYFP^-$ cells (Figure 3H) identified only 18 differentially expressed genes (Table S5), indicating that AAV1_P5 transduction affects their transcriptome only mildly. Furthermore, we did not find any concerted upregulation of viral response genes in this comparison or when comparing $eYFP^+$ cells to $eYFP^-$ $NeoR^+$ cells (Figure S4M) or naive v-SVZ lineage cells from Kalamakis et al.[12] (Figure S4N). In conclusion, we have combined scRNA-seq with lineage tracing using AAV1_P5 and found that transduction does not affect the expression of proliferation markers and overall only minimally affects the transcriptomic readout.

We next tested whether the transduction efficiency could be further optimized by the selection of promoter and number of injected vgs per mouse. To this end, we now packaged the CMV_Cre construct into the AAV1_P5 capsid and injected either 10^9 vgs per mouse as in Figures 2E–2J or an increased concentration of 10^{10} vgs per mouse into tdTomato-flox mouse brains (Figure S5A). In all conditions, tdTomato-labeled cells were detected at high numbers in the v-SVZ, confirming specific v-SVZ targeting by the AAV1_P5 capsid (Figures S5B–S5D). Transduction of cells was over 60 times higher with the CMV_Cre construct (319.9 cells per section) (Figure S5D) than with CAG_Cre (4.8 cells per section) (Figure 2J) when injecting 10^9 vgs per mouse. By increasing the number of injected vgs from 10^9 to 10^{10}, we were able to further increase the number of labeled cells (Figure S5D) including NSCs/TAPs and ependymal cells (Figures S5F and S5G). However, the increased viral load also moderately increased the proportion of labeled cells located outside of the v-SVZ (Figure S5E).

We finally assessed the neurogenic function of transduced NSCs *in vivo*. To this end, we assessed the number of transduced NSCs in the v-SVZ and their neuronal progeny in the OB. 10^{10} vgs/mouse of AAV1_P5 harboring the CMV_Cre construct were injected into the lateral ventricles of tdTomato-flox mice, and at 35 dpi, the number of labeled NSCs in the v-SVZ and OB interneurons was assessed (Figure 4A). We observed a high heterogeneity in the number of labeled cells probably due to differences in the injection site. It should be noted that the given coordinates are always relative to the average brain of a WT mouse. Therefore, smallest differences in the volume or orientation of the ventricle by slight inclination of the head within the stereotactic frame are potential sources of variability of the injection site. One set of animals exhibited a lower number of labeled cells in the SVZ and OB than the other (Figure 4B). Although a trend toward a reduced number of NSCs/TAPs at 35 dpi was detectable, NSCs still remained in the v-SVZ at this late time point (Figure 4C), suggesting that AAV1_P5 also targeted qNSCs.

To estimate the extent of targeting of the NSC compartment, we took advantage of our previously developed mathematical modeling framework for stem cell dynamics of v-SVZ.[12] First, we extended our previously established model and calibrated it to the experimentally observed dynamics of TAPs and OB neurons (see Supplemental material [Mathematical modeling]). Instead of fitting the model to average cell counts across mice, we subdivided the data into two groups, with higher and lower labeling, as animals with high labeling in the v-SVZ exhibited a much higher number of labeled cells in the OB than animals with lower labeling (Figures 4D and 4E). Fitting of the model to the data, assuming that viral transduction does not affect cell kinetics and that the observed heterogeneity comes from different numbers of initially labeled NSCs and TAPs, the model indicates that approximately 57% of NSCs are labeled in the high-label group and 26% of NSCs in the other group (see Supplemental material). Moreover, the model indicates that in the low-labeled group, barely any TAP would be labeled at the initial time, whereas in the other group, a higher number of TAPs are initially labeled.

Finally, we employed our model to address whether the observed labeling would arise from direct targeting of qNSCs, aNSCs, or both. To this end, we simulated two scenarios where either only qNSCs or only aNSCs are targeted (Figure 4F). Our simulation indicates that the ratio of labeled qNSCs to aNSCs reaches the same value in both scenarios after approximately 4 days, due to transitions between the quiescent and active state. Altogether, comparison of a model fit to data is in line with the hypothesis that the number of initially transduced NSCs and TAPs differs between the two groups, that the cell dynamics exhibited by transduced cells are comparable to non-

model fit and data. (D) Comparison of the fit to data from the high-labeled group 1. (E) Comparison to data from low-labeled group 2. The model was fit to both groups simultaneously. Only the number of initially labeled NSCs and TAPs differs between the two groups. (F) Redistribution of labeled NSC between the active and the quiescent state. We compare two scenarios. In the first scenario (red lines) the virus targets only aNSC. In the second scenario (blue lines), the virus targets only qNSCs. After 4 days, the number of labeled aNSC is identical for both scenarios (lower panel). The same applies to the number of labeled qNSCs (upper panel), since aNSC can become quiescent after division, and qNSCs can become activated. Black dots indicate FACS quantifications of NSCs labeled by the AAV1_P5_YFP adenovirus (as shown in Figures S3C and S3D). Virus injection took place at time 0. (G) Experimental layout of FACS quantification of TiCY mice to analyze labeling efficiency of the v-SVZ and OB using AAV1_P5_Cre. (H) Quantification of FACS events: total NSC count in the v-SVZ; proportion of aNSC to qNSC; proportion of $eYFP^+$ NSCs and TAPs; and proportion of $eYFP^+$ neuroblasts in the v-SVZ and OB. SV40, simian virus 40 poly(A) signal.

transduced cells, and that AAV5_P5 can target up to 57% of the NSC pool.

To validate the model prediction of the label efficiency of the AAV1_P5 vector, we performed a FACS quantification experiment to directly assess the percentage of NSC and progeny that is labeled by the virus 8 dpi (Figure 4G). 5-month-old TiCY mice were injected with 10^9 vgs/mouse of AAV1_P5 harboring the CMV_Cre construct. FACS quantification analysis was performed as described previously (Figures S3C, S3D, S6A, and S6B), and the results showed 30.46% labeling efficiency for NSCs (Figure 4H; mean eYFP$^+$-percentage of both samples), which is close to the 26% labeling efficiency predicted by the mathematical model (Supplemental material). The model also showed a good fit when applied to the FACS quantification experiment performed to choose the best candidate between AAV1_P5 and AAV9_A2. Moreover, the prediction of a high labeling group was validated by the observed labeling rate in the single-cell transcriptomics analysis (see Supplemental material).

DISCUSSION

Altogether, in this study, we have performed barcode-based *in vitro* and *in vivo* high-throughput screenings of two libraries of WT and engineered AAV capsids.[53] Targeting of NSCs and especially qNSCs has only been demonstrated in the hippocampal dentate gyrus with the capsid AAV r3.45[64] and the African green monkey isolate AAV4,[65] as well as recently in the v-SVZ using the newly engineered AAV variant SCH9.[42]

Here, we have identified two lead candidates for efficient targeting of NSCs *ex vivo* and *in vivo*. We particularly characterized the novel capsid AAV1_P5 as highly region specific at targeting cells of the v-SVZ layer, including ependymal cells and NSCs, by IHC, FACS quantification, and scRNA-seq. We moreover show by IHC and scRNA-seq that NSCs targeted with AAV1_P5 were not noticeably affected in their migration and transcriptome and readily generated OB neurons. Furthermore, we demonstrate that the engineered capsid AAV1_P5 also labels qNSCs. We propose that qNSC labeling cannot only be achieved by direct targeting of qNSCs but also indirectly through transduction of aNSCs that would later give rise to qNSCs. Indeed, based on mathematical modeling of FACS counts, we predict that labeled cells redistribute between those states within less than 1 week. Therefore, the initial labeling proportion of qNSCs to aNSC is not crucial when stem cell dynamics are observed on a longer timescale.

AAV1_P5 clearly targets cells in the v-SVZ. Which molecular mechanism leads to efficient targeting of v-SVZ cells by AAV1_P5 is unknown. It was previously shown that the SCH9 variant binds heparan sulfate proteoglycans and galactose, both of which are present on NSCs in the v-SVZ.[42] AAV1_P5 may act via a similar mechanism that would lead to a specific tropism for v-SVZ cells, but other molecular mechanisms are also possible. For instance, AAV1_P5 may be unable to migrate deeply into the ventricular wall, which would favor transduction of NSCs, or it may be that AAV1_P5 has properties that favor its survival or activity in the cerebrospinal fluid. To date, there are only a few cases where such mechanisms underlying altered viral properties of synthetic AAV capsids have been successfully elucidated.[66–69] One example is the use of the $\alpha v\beta 8$ integrin as a receptor for a keratinocyte-specific AAV2.[66] Another example was reported by several labs that have recently identified an interaction of AAV-PHP.B (a peptide-modified AAV9) with the glycosylphosphatidylinositol (GPI)-linked protein LY6A.[67–69] Other than these, however, the receptors or interactions that are targeted by peptide-engineered or shuffled AAV variants typically remain enigmatic, as do the intracellular mechanisms underlying their novel features. Hence, the identification of the receptor for AAV1_P5 will be the subject of future studies. In this looming work, it will then also be interesting to study whether AAV1_P5 interacts with other host cell factors that have been identified over the years as critical for transduction with WT capsids, such as the widely used AAV receptor AAVR[70] or intracellular elements such as the proteasome.[71]

As a proof of concept, we show that AAV1_P5 labeling can be combined with scRNA-seq to characterize the transcriptomes of NSCs and their progeny from different brain regions. Surprisingly, the number of transduced off-target cells in this experiment (Figure 3B) was much lower than in our previous FACS-based experiments (Figure 2J). A possible explanation is that the main source of off targets, ependymal cells, are hard to detect in scRNA-seq experiments: a previous study[72] isolated 9,804 cells from the v-SVZ without marker preselection, and only 46 of them were ependymal cells. As a result, the low off-target percentages reported in Figure 3B should only be expected in scRNA-seq experiments. Our method of AAV1_P5 labeling, followed by scRNA-seq, paves the way for more complex lineage tracing experiments *in vivo*. Recent studies have used CRISPR-Cas9-induced genomic scars combined with scRNA-seq to enable clonal lineage tracing in embryonic development.[73,74] AAVs could be used to induce genomic scars in specific cells at specific time points to enable clonal lineage tracing in adult tissues. We use our scRNA-seq data to further corroborate our assessment that NSCs are efficiently targeted and remain functional after transduction. Future studies using electrophysiology are required to assess whether the progeny generated by transduced NSCs is fully functional and able to integrate into the neuronal circuits of the OB.

Finally, we identified the combination of the CMV promoter and AAV1_P5 capsid as ideally suited to efficiently transduce NSCs in the v-SVZ. Our finding that the CMV outperforms the CAG promoter differs from previous studies overexpressing plasmids via *in utero* electroporation in the mouse brain.[75,76] We also found that increased viral load resulted in higher labeling efficiency as expected but at the cost of some regional specificity. This trade-off must be considered when designing future experiments; e.g., when targeting cells outside of the v-SVZ must be absolutely avoided, it is advisable to inject a lower amount of vg. We conclude that the CMV promoter should be preferred over CAG when using AAV1_P5, injecting 10^{10} vgs per mouse or alternatively 10^9 when regional specificity is crucial.

Future experiments will be needed to unravel and understand the mechanisms governing the properties of our candidates. Altogether, we believe that our study opens tantalizing avenues to genetically modify NSCs in their *in vivo* environment for the treatment of CNS disorders or brain tumors.

MATERIALS AND METHODS

Animals

In this work, the mouse lines C57BL/6N, TdTomato-flox, and TiCY were used. All mice were male and were age matched to 8 weeks, except for TiCY mice, which were 5 months old (for FACS quantification) and 3 months old (for scRNA-seq). Animals were housed in the animal facilities of the German Cancer Research Center (DKFZ) at a 12-h dark/light cycle with free access to food and water. All animal experiments were performed in accordance with the institutional guidelines of the DKFZ and were approved by the "Regierungspräsidium Karlsruhe" (Germany).

AAV vector production

The production of the AAV-barcoded library was done as previously published[77,78] with some modifications: 159 distinct barcodes were inserted into the 3′ UTR of a YFP reporter under the control of a CMV promoter and encoded in a self-complementary AAV genome. Each of the barcodes was assigned to one AAV capsid from a total of 183 variants, which are described in more detail in the accompanying manuscript by Weinmann et al.[53] Altogether, this library production included 12 AAV-WTs (AAV1 to AAV9, AVVrh.10, AAVpo.1, and AAV12), 94 peptide display mutants, and 71 capsid chimeras, which were created by DNA family shuffling. Isolation of synthetic capsids was performed in specific tissues or in our recent screens of AAV libraries in cultured cells, mouse liver tissue, or muscle.[79]. These synthetic capsids include a set of 12 AAV serotypes that were previously modified by insertion of over 20 different peptides in exposed capsid loops and that were recently characterized in established or primary cells.[79] In the work of Weinmann et al.,[53] all barcoded capsids were pooled in different combinations to finally obtain three distinct libraries (#1, #2 [not used in the present work], and #3), with 91, 82, and 157 variants. Further details on library composition are found in the supplemental information of Weinmann et al.[53] All capsid variants are detailed in Table S4. HEK293T cells were cultured in DMEM (Gibco) supplemented with 10% fetal bovine serum (Merck), 1% penicillin (pen)/streptomycin (strep) (Gibco; 10,000 U/mL pen and 10,000 μg/mL strep) and 1% L-glutamine (Gibco; 200 mM) at 37°C and 5% CO_2. AAV vectors were produced by seeding HEK293T cells (4.5×10^6 cells per dish) on 90−150, 15 cm tissue-culture dishes (Sigma). 2 days later, we performed a polyethylenimine (PEI; Polysciences) triple transfection by mixing 44.1 μg (3 × 14.7 μg) DNA of (1) a plasmid containing the recombinant AAV genome of interest, (2) an AAV helper plasmid carrying AAV *rep* and *cap* genes, and (3) a plasmid providing adenoviral helper functions for AAV production in a total volume of 790 μL H_2O per culture dish. Separately, PEI (113.7 μg) and H_2O were mixed in a total volume of 790 μL per dish, and NaCl (300 nM) was added 1:1 to both, PEI, or DNA solution. PEI was added dropwise to DNA and incubated for 10 min at room temperature, before finally adding the DNA/PEI mixture to the culture dish. 3 days later, cells were scraped off in the media and collected by centrifugation (400 g, 15 min). The pellet was dissolved in 0.5 mL virus lysis solution (50 mM Tris HCl; Sigma), 2 mM $MgCl_2$ (Sigma), and 150 mM NaCl (Thermo Fisher Scientific; pH 8.5) and was immediately frozen at −80°C. In total, 5× freeze-thaw cycles were performed with the cell pellet prior to sonication for 1 min, 20 s. The cell lysate was treated with Benzonase (75 U/μL; Merck) for 1 h at 37°C, followed by a centrifugation step at 4,000 × *g* for 15 min. $CaCl_2$ was added to a final concentration of 25 mM, and the solution was incubated for 1 h on ice, followed by centrifugation at 10,000 g for 15 min at 4°C. The supernatant was harvested, and a $^1/_4$ vol of a 40% polyethylene glycol (PEG 8000; BioChemica) and 1.915 M NaCl (Thermo Fisher Scientific) solution was added prior to incubation for 3 h on ice. After centrifugation for 30 min at 2,500 × *g* and 4°C, the pellet was dissolved in resuspension buffer (50 mM HEPES; Gibco), 0.15 M NaCl (Thermo Fisher Scientific), and 25 mM EDTA (Sigma) and was dissolved overnight. The solution was then centrifuged for 30 min at 2,500 × *g* and 4°C, and the supernatant was mixed with cesium chloride (CsCl; Sigma) to a final concentration of 0.55 g/mL. The refractive index was adjusted to 1.3710 using additional CsCl or buffer, as needed. Next, the vector particles were purified using CsCl gradient density centrifugation. Fractions with a refractive index of 1.3711 to 1.3766 comprising DNA-containing AAV particles were pooled and dialyzed against 1× PBS with a Slide-A-Lyzer dialysis cassette according to the manufacturer's instructions (Thermo Fisher Scientific). Subsequently, the samples were concentrated by using an Amicon Ultra Centrifugal Filter (Millipore; 100,000 nominal molecular weight limit [NMWL], used to retain the viral particles) following the manufacturer's instructions. The volume of the samples was reduced to 250−300 μL. AAV vectors were finally aliquoted and stored at −80°C.

The production of the AAV1_P5_YFP and AAV9_A2_YFP viruses for the FACS analysis experiment was done as described above, with the only modification that the vectors were purified using two iodixanol gradients. Of note, the barcoded AAV library construct as well as the YFP construct were engineered as double-stranded AAV vectors. The constructs for CAG_Cre::GFP and CMV_Cre were engineered as a single-stranded AAV vector.

AAV vector titration

AAV vectors were titrated using quantitative real-time PCR as described in Senís et al.[80] For the CAG_Cre::GFP construct, the primers and probe GFP_forward (fwd), GFP_reverse (rev), and GFP_probe were used, whereas Cre_fwd, Cre_rev, and Cre_probe were used for the CMV_Cre construct (Table S1). The qPCR was performed on a C1000 Touch Thermal Cycler equipped with a CFX384 Real-Time System (Bio-Rad) with the following conditions: initial melting for 10 min at 95°C, followed by 40 cycles of denaturation for 10 s at 95°C and annealing/extension for 30 s at 55°C. A standard curve was considered as reliable when the coefficient of determination (R^2) was greater than 0.985.

Stereotactic injection

AAV vectors were stereotactically injected into the lateral ventricle by using the following coordinates calculated to bregma: anterior-posterior (AP) −0.5 mm, medio-lateral (ML) −1.1 mm, dorso-ventral (DV) 2.4 mm. Mice received either 10^9 or 10^{10} vgs/mouse in a total volume of 10 μL. The AAV libraries were stereotactically injected into the lateral ventricle by using the following coordinates calculated to bregma: AP −0.5 mm, ML −1.1 mm, DV 2.4 mm. Mice received 4 × 10^{10} vgs/mouse in a total volume of 2 μL. *Ex vivo*-manipulated cells (7,000 FACS events) were injected into two areas of the v-SVZ using the following coordinates calculated to bregma: AP 0.7 mm, ML 1.6 mm, DV 2 mm and AP 0 mm, ML 1.7 mm, DV 2 mm.

Cell isolation and *in vitro* cultivation

The lateral v-SVZ was micro-dissected as whole mount as previously described.[81] Tissue of single mice was digested with trypsin and DNase according to the guidelines of the Neural Tissue Dissociation Kit (trypsin; Miltenyi Biotec) using a Gentle MACS Dissociator (Miltenyi Biotec). Cells were cultured and expanded for 8−12 days in neurobasal medium (Gibco) supplemented with B27 (Gibco), heparin (Sigma), glutamine (Gibco), pen/strep (Gibco), epidermal growth factor (EGF; PromoKine), and fibroblast growth factor (FGF; PeloBiotech), as reported in Walker and Kempermann.[82]

In vitro transduction of cultured NSCs

For RNA-seq, NSCs were seeded in 48-well plates (Greiner Bio-One) and incubated overnight. AAV library #1 or library #3 (same libraries as in Weinmann et al.;[53] multiplicity of infection [MOI]: 10,000) was added to the media and remained for the duration of 7 days. For IHC, Labtek chambers (Thermo Fisher Scientific) were coated with Poly D-Lysine (PDL; Sigma)/laminin (Sigma), and NSCs were seeded at a density of 2 × 10^4 cells per square centimeter overnight. AAVs were added (MOI: 10,000) and remained in the media for 1, 3, 5, or 7 days.

Single-cell transcriptomic profiling by 10× chromium 3′ sequencing

Stereotactic injection, single-cell suspension preparation, and sorting

3-month-old TiCY mice were stereotactically injected into the lateral ventricle with 10^9 vgs of the AAV1_P5_Cre capsid. After 5 weeks of chase time, the mice were sacrificed, and the SVZ, striatum, RMS, and OB were isolated. The latter three tissues were pooled as a single tube and were named RoB. From these tissues, a single-cell suspension was prepared as described before (Cell isolation and in vitro cultivation). From the SVZ, the cells sorted were eYFP$^+$ (O4/CD45/Ter119 negative, eYFP$^+$) and from the eYFP-negative (eYFP$^-$) cells, only GLAST$^+$ cells. From the RoB, only eYFP$^+$ cells were sorted. The total number of sorted events for the 2 days of the experiment was 12,000 for SVZ cells and 5,800 for cells of the RoB. 2 TiCY mice were pooled for each sorting day. All of the cells were sorted in a volume of 50 μL of fetal calf serum (FCS) 10% in PBS, from which 45 μL was used for loading the Chromium Next GEM Chip G.

Library preparation, sequencing, and mapping

One library per each sorting day was prepared by following the manufacturer's protocol (Chromium Next GEM Single Cell 3′ version [v.] 3.1) and sequenced on a NovaSeq 6K PE 100 S1.

In order to quantify eYFP and NeoR (NeoR/kanamycin resistance gene) expression, entries for these transgenes were manually added to the FASTA and Gene Transfer Format (GTF) files of the mouse reference genome mm10-3.0.0 provided by 10X Genomics. scRNA-seq reads were pseudoaligned and further processed with kallisto|bustools[83,84] to generate a gene × barcode count matrix.

Computational analysis of scRNA-seq data

Cell barcodes with less than 1,500 unique molecular identifiers (UMIs) or more than 15% mitochondrial reads were filtered, and the remaining cells were further analyzed in Scanpy v.1.5.1.[85] We used Scanpy to calculate G2/M- and S-phase scores for all cells, based on their expression of G2/M- and S-phase marker genes from Tirosh et al.[86] These scores were then regressed out of the count data to reduce the influence of the cell cycle on clustering. The first 50 principal components of 3,324 highly variable genes were used for 2D visualization with Uniform Manifold Approximation and Projection (UMAP; n_neighbors = 35) and cell clustering with the Leiden algorithm (resolution = 0.5). Cell clusters were assigned to cell types based on the expression of NSC lineage marker genes previously described in Kalamakis et al.[12] and Llorens-Bobadilla et al.[6] and ependymal cell markers from Shah et al.[58] (Figure 3C). To identify the location of cells from RoB, kernel density estimates of cell density in the 2D UMAP space were calculated for both samples. Since sample #1 contains more RoB cells, and sample #2 contains more v-SVZ cells, we subtracted both densities to highlight cells that most likely stem from RoB (orange cells in Figure S4H).

In order to estimate transduction efficiency from scRNA-seq data, we used the following model, based on the usual approach of modeling RNA-seq counts by the NB distribution:

For non-transduced cells, we assume that they express NeoR such that an expected fraction μ_R of all of their mRNA transcripts originates from this gene. For each individual cell j, the actual expression strength q_j^R of the gene varies around this expectation according to a gamma distribution with mean μ_R and variance $\alpha_R \mu_R$. The observed number of UMIs is then modeled as a Poisson variable: $k_j^R \big| q_j^R \sim Pois(s_j q_j^R)$, where s_j is the total UMI count for cell j, summed over all genes. Marginalizing out q_j^Rhttps://www.codecogs.com/eqnedit.php?latex=q_j%5E%5Ctext%7BR%7D - 0, we find k_j^R https://www.codecogs.com/eqnedit.php?latex=k_j%5E%5Ctext%7BR%7D - 0 to follow a NB distribution with mean $s_j \mu_R$https://www.codecogs.com/eqnedit.php?latex=s_j%20%5Cmu_%5Ctext%7BR%7D - 0 and dispersionα_R. As we are looking at a non-transduced cell, the UMI count k_j^Y https://www.codecogs.com/eqnedit.php?latex=k%5E%5Ctext%7BY%7D_j - 0 for eYFP is, of course, zero.

Similarly, we write k_j^Yhttps://www.codecogs.com/eqnedit.php?latex=k_j%5E%5Ctext%7BY%7D - 0, $\mu_Y$https://www.codecogs.com/eqnedit.php?latex=%5Cmu_%5Ctext%7BY%7D - 0, and $\alpha_Y$https://www.codecogs.com/eqnedit.php?latex=%5Calpha_%5Ctext%7BY%7D - 0 for the corresponding quantities of eYFP, expressed by transduced cells. For a fully transduced cell j, we therefore have https://www.codecogs.com/eqnedit.php?latex=k_j%5E%5Ctext%7BY%7D%20%5Csim%20%5Ctext%7BNB%7D(%20s_j%5Cmu_%5Ctext%7BY%7D%2C%20%5Calpha_%5Ctext%7BY%7D%20) - 0 $k_j^Y \sim NB(s_j\mu_Y, \alpha_Y)$ but https://www.codecogs.com/eqnedit.php?latex=k_j%5E%5Ctext%7BR%7D%3D0 - 0 $k_j^R = 0$. For transduced cells with incomplete or heterozygous Cre-mediated excision, we should see both genes expressed but will model the expression strength to be only one-half as strong.

The likelihood of observing UMI counts https://www.codecogs.com/eqnedit.php?latex=k_j%5E%5Ctext%7BR%7D - 0 k_j^R and https://www.codecogs.com/eqnedit.php?latex=k_j%5E%5Ctext%7BY%7D - 0 k_j^Y for a given cell j therefore depends on the parameters just mentioned as well as on the probabilities $p_U$https://www.codecogs.com/eqnedit.php?latex=p_%5Ctext%7BU%7D - 0 that the cell is not transduced, $p_T$https://www.codecogs.com/eqnedit.php?latex=p_%5Ctext%7BT%7D - 0 that it is fully transduced, and https://www.codecogs.com/eqnedit.php?latex=p_%5Ctext%7BP%7D%20%3D%201-p_%5Ctext%7BU%7D-p_%5Ctext%7BT%7D - 0$p_P = 1 - p_U - p_T$ that it is partially transduced. We write the likelihood as

$$L_j = p_U f_{NB}\left(k_j^R; \mu_R, \alpha_R\right)\delta\left(k_j^Y\right) +$$

$$+ p_T\delta\left(k_j^R\right) f_{NB}\left(k_j^Y; \mu_Y, \alpha_Y\right) +$$

$$+ p_P f_{NB}\left(k_j^R; \mu_R \Big/ 2, \alpha_R\right) f_{NB}\left(k_j^Y; \mu_Y \Big/ 2, \alpha_Y\right),$$

where https://www.codecogs.com/eqnedit.php?latex=f_%5Ctext%7BNB%7D(k%3B%5Cmu%2C%5Calpha) - 0$f_{NB}(k; \mu, \alpha)$ is the probability to observe k counts under a NB distribution with mean μ and dispersion α, and δ is the zero indicator function; i.e., https://www.codecogs.com/eqnedit.php?latex=%5Cdelta(k)%3D0 - 0 $\delta(k) = 0$ for $k \neq 0$https://www.codecogs.com/eqnedit.php?latex=k%5Cneq%200 - 0 but https://www.codecogs.com/eqnedit.php?latex=%5Cdelta(0)%3D1 - 0 $\delta(0) = 1$.

Given all the k_j https://www.codecogs.com/eqnedit.php?latex=k_j - 0 and s_j, we obtain estimates for the transduction efficiency $p_T$https://www.codecogs.com/eqnedit.php?latex=p_T - 0 and for p_U and p_P as well as for the nuisance parameters μ_R https://www.codecogs.com/eqnedit.php?latex=%5Cmu_R - 0, α_R, $\mu_Y$https://www.codecogs.com/eqnedit.php?latex=%5Cmu_Y - 0, and $\alpha_Y$https://www.codecogs.com/eqnedit.php?latex=%5Calpha_Y - 0 by numerically maximizing the log likelihood https://www.codecogs.com/eqnedit.php?latex=l%3D%5Csum_j%5Clog%20L_j - 0 $l = \sum_j logL_j$ using the R function optim.

We mention two technical details: first, in order to give all optimization parameters full domain over all of $\mathbb{R}$, we used parameter transformations in the optimization, namely exponentiating the μs and αs, and logit-transforming the probabilities p https://www.codecogs.com/eqnedit.php?latex=p - 0 and q obtained from reparametrizing $p_T = p(1-q)$, $p_U = 1 - p$, $p_P = p_q$https://www.codecogs.com/eqnedit.php?latex=p_P%20%3D%20pq - 0. Second, in order to improve identifiability in case of low values for $p_U$https://www.codecogs.com/eqnedit.php?latex=p_U - 0, we enforced a minimum value for $\mu_R$https://www.codecogs.com/eqnedit.php?latex=%5Cmu_R - 0 by adding to the likelihood a penalty term $f_{pty}(\mu_R)$, where $f_{pty} = 1/(1 + e^{9\times10^5 x - 9})$ https://www.codecogs.com/eqnedit.php?latex=f_%5Ctext%7Bpty%7D%3D1%2F(1%2Be%5E%7B9%5Ctimes10%5E%7B5%7Dx-9%7D) - 0 is a sigmoid that vanishes for https://www.codecogs.com/eqnedit.php?latex=%5Cmu_R%20%5Cgtrsim%20%5Cmu_%7BR_%7B%5Ctext%7Bmin%7D%7D%7D%3D2%5Ctimes10%5E%7B-5%7D - 0 $\mu_R \gtrsim \mu_{R_{min}} = 2 \times 10^{-5}$.

Differential gene expression was assessed by summing UMI counts of cells within a group to yield pseudobulk samples for testing in DESeq2 v.1.29.7.[87] eYFP$^+$ cells were tested against both eYFP$^-$ cells and eYFP$^-$ NeoR$^+$ cells. Testing eYFP$^+$ versus eYFP$^-$ has the advantage of greater statistical power due to higher cell numbers, but some eYFP$^-$ cells may be transduced cells with eYFP dropout. Thus, we performed both comparisons, yielding similar results. To account for the unequal distribution of eYFP$^+$ and eYFP$^-$ cells along the lineage (Figure S4H), pseudobulk groups were formed per cluster and sample, and the cluster identity was added as a covariate in DESeq2. To enable comparison of v-SVZ cells from [12] with our eYFP$^+$ cells, both datasets were integrated with Seurat's SCTransform integration workflow[88] using our cells as reference. The integrated dataset was clustered, and differential expression was assessed as above, using the shared clusters as covariate. Genes with the Gene Ontology (GO) term "GO: 0009615—response to virus" were highlighted.

FACS

Generation of single-cell suspension was performed as described in Llorens-Bobadilla et al.[6] Cells were stained with the following antibodies: O4-allophycocyanin (APC) and O4-APC-Vio770 (Miltenyi; diluted 1:50), Ter119-APC-Cy7 (BioLegend; 1:100), CD45-APC-Cy7 (Becton Dickinson [BD]; 1:200), GLAST (ACSA-1)-phycoerythrin (PE; Miltenyi: 1:20), CD9-eFluor450 (eBioscience; 1:300), Alexa647::EGF (Life Technologies; 1:100), polysialylated neuronal cell adhesion molecule (PSA-NCAM)-PE-Vio770 (Miltenyi; 1:75), Prominin1- peridinin-chlorophyll-protein PerCP-eFluor 710 (eBioscience; 1:75), CD24-PE-Cy7 (eBioscience; 1:75), and Sytox Blue (Life Technologies; 1:1,000). For RNA-seq, cells were directly sorted into 100 μL of the PicoPure RNA Isolation Kit (Thermo Fisher Scientific) extraction buffer. For *ex vivo* transduction, NSCs were sorted into growth factor-free Neurobasal medium (NBM).

FACS analysis of AAV-injected mice

FACS analysis for testing the transduction efficiency of the candidate viruses was performed by two methods. The first method consisted of

injecting 5-month-old TiCY mice with the AAV1_P5_Cre virus, and after 8 days, SVZ and OB cells were FACS analyzed (Figures 4G and 4H). In the second method, we injected 2-month-old C57BL/6N mice with AAV1_P5_YFP and AAV9_A2_YFP viruses and analyzed them after 6 days (Figures S3C and S3D).

For FACS quantification of AAV-injected NSC/progeny, cells were sorted with the following antibodies: O4-APC-Vio770 (Miltenyi; diluted 1:100), CD45-APC-Cy7 (BD; 1:200), Ter119-APC-Cy7 (BioLegend; 1:100), GLAST (ACSA-1)-PE (Miltenyi; 1:50), Prominin1-APC (eBioscience; 1:75), PSA-NCAM-PE-Vio770 (Miltenyi; 1:50), Texas-Red::EGF (Life Technologies; 1:75).

Ex vivo treatment of NSCs

FACS NSCs were transduced with AAV (MOI: 10,000) and incubated on ice for 2–3 h. Cells were centrifuged for 15 min at 300 × *g*, 4°C, and were washed twice with PBS. The pellet was dissolved in 4 μL PBS.

RNA isolation and cDNA synthesis

RNA was isolated by using the PicoPure RNA Isolation Kit (Thermo Fisher Scientific). For RNA isolation of *in vitro*-transduced cells, 1,500 cultured NSCs per set were lysed in 100 μL extraction buffer. For isolation of FACS *in vivo*-transduced cells, batches of 500 cells or less were generated and were lysed in 100 μL extraction buffer. Up to 6 batches (2,500 cells) were obtained per set, depending on the cell type (Tables S2 and S3). The cell-containing extraction buffer was incubated for 30 min at 42°C, and the lysate was frozen at −80°C to increase the amount of isolated RNA. The cell lysate was mixed 1:1 with 70% ethanol, and RNA was extracted according to the guidelines of the PicoPure RNA Isolation Kit (Thermo Fisher Scientific). RNA was dissolved in 11 μL nuclease-free H_2O. The cDNA synthesis was performed as described in Picelli et al.[89] by using locked nucleic acid-template switch oligo (TSO) (Table S1) and by using either 14 cycles for *in vitro*-cultured NSCs or 15 cycles (>300 cells per batch) or 16 cycles (<300 cells per batch) for FACS *in vivo*-transduced cells for the cDNA enrichment step. After purification[89] using AMPure XP beads (Beckman Coulter), cDNA was dissolved in 10 μL H_2O.

Barcode amplification PCR and NGS library preparation

Barcodes were PCR amplified by using 10 ng cDNA as input material. Therefore, the PCR primers barcode_forward (Bar_fwd) and barcode reverse (Bar_rev) that bind up and downstream of the 15-bp-long barcodes within the according cDNA were engineered, and the Phusion High-Fidelity DNA Polymerase (Thermo Fisher Scientific) was used according to its manual in combination with 10 mM dNTPs (Thermo Fisher Scientific) (Table S1). The PCR was performed on a T100 Thermal Cycler (Bio-Rad) with the following conditions: initiation for 30 s at 98°C, followed by 35 cycles of denaturation for 10 s at 98°C, annealing/extension for 20 s at 72°C, and a final step for 5 min at 72°C. The result was a 113-bp-long PCR amplicon that includes the barcode with its 15-bp-long random DNA sequence. The PCR amplicon was AMPure XP Bead purified (Beckman Coulter)[89] with a bead:sample ratio of 0.8:1 in the first round and 1:1 in the second round. After this step, the samples were enriched for the barcode containing amplicon, and of course, the samples potentially contained the range of up to 157 different AAV barcodes, which were initially used. Next, 10 ng or 15 ng (library #1 or #3, respectively) of PCR amplicon was used for NGS library preparation with the NEBNext Chromatin Immunoprecipitation (ChIP)-Seq Library Prep Reagent Set for Illumina (NEB) for samples from library #1 and the NEBNext Ultra II DNA Library Prep Kit for Illumina (NEB) for samples from library #3. Multiplexed libraries were generated by following the manual and by using the NEBNext Multiplex Oligos for Illumina (NEB). All multiplexed samples for library #1 and library #3 are listed in Tables S2 and S3. For sequencing, up to 50% of PhiX were spiked in to increase the complexity of the library.

Immunocytochemistry

Cells were washed 3 × 5 min in PBS at room temperature, followed by a 30 min blocking step in PBS^{2+} (PBS with 0.3% horse serum [Millipore] and 0.3% Triton X-100 [Sigma]) at room temperature. Subsequently, the cells were incubated overnight in PBS^{2+} containing primary antibodies at 4°C. Cells were washed in PBS for 3× 5 min at room temperature and were incubated with secondary antibodies in PBS^{2+} for 1 h in the dark at room temperature. Afterward, cells were washed 3× 5 min in PBS and were mounted with Fluoromount G (eBioscience). The following antibodies were used: chicken anti-GFP (Aves; 1:1,000) and goat anti-mCherry (SICGEN; 1:1,000). Nuclei were counterstained with Hoechst 33342 (BioTrend; 1:3,000).

Tissue preparation

Animals were sacrificed by using an overdose of ketamine (120 mg/kg)/xylazine (20 mg/kg) and were subsequently transcardially perfused with ice-cold 20 mL 1× Hank's balanced salt solution (HBSS; Gibco) and 10 mL of 4% paraformaldehyde (Carl Roth). The brains were dissected and postfixed in 4% paraformaldehyde overnight at 4°C. A Leica VT1200 Vibratome was used to cut the tissue in 50 μm (v-SVZ)- or 70 μm (OB)-thick coronal sections. From each mouse, three to six identical brain sections every 100 μm (v-SVZ) or 140 μm (OB) along the coronal axis were used for staining. Brain sections for staining the v-SVZ were harvested from 0.5 to 1.1 mm anterior to the bregma.

IHC

Brain sections were washed 4× 10 min in Tris-buffered saline (TBS) at room temperature, followed by a 1-h blocking step in TBS^{2+} (TBS with 0.3% horse serum [Millipore] and 0.3% Triton X-100 [Sigma]) at room temperature. The tissue was transferred to 0.5 mL Safe Lock Reaction Tubes containing 200 μL TBS^{2+} including primary antibodies. Samples were incubated for 24–48 h at 4°C. Tissue samples were washed 4× 10 min in TBS at room temperature, followed by a 30-min blocking step in TBS^{2+} at room temperature. Brain sections were transferred to 0.5 mL Safe Lock Reaction Tubes containing 200 μl TBS^{2+} including secondary antibodies. Samples were incubated in the dark for 2 h at room temperature. Subsequently, brain slices were washed 4× 10 min in TBS at room temperature and were mounted on glass slides with Fluoromount G (eBioscience). The following antibodies were used: mouse anti-Sox2 (Abcam; 1:100),

guinea pig anti-DCX (Merck; 1:400), rabbit anti-S100B (Abcam; 1:100), goat anti-mCherry (SICGEN; 1:1,000), and chicken anti-GFAP (GeneTex; 1:500). Nuclei were counterstained with Hoechst 33342 (BioTrend; 1:3,000).

Microscopy and cell quantification

All images were acquired with a Leica TCS SP5 Acousto-Optical Beam Splitter(AOBS) confocal microscope equipped with a UV diode 405 nm laser, an argon multiline (458–514 nm) laser, a helium-neon 561 nm laser, and a helium-neon 633 nm laser. Images were acquired as multichannel confocal stacks (z plane distance 3 μm) in 8-bit format by using a 20× or 40× oil-immersion objective at a resolution of 1,024 × 1,024 and 200 Hz. For quantification of the v-SVZ and total brain sections, tile scans of the whole ventricle or the whole coronal brain section were acquired with a total z stack size of 25 μm. To quantify the OB, tile scans of the whole OB covering the tissue thickness were acquired. For stained cells from *in vitro* culture, 4–9 fields of view were imaged. For representative images (2,048 × 2,048 resolution, 100 Hz), the maximum intensity of a variable number of z planes was stacked to generate the final z projections. Representative images were cropped, transformed to RGB color format, and assembled into figures with Inkscape (inkscape.org). For cell quantification, ImageJ (NIH) was used including the plug-in cell counter to navigate through the z stacks. To quantify cells in the OB, the volume of the OB was calculated by multiplying the entire area of every OB section (including the glomerular layer [GLL]) with the entire z stack size. Then we converted cubed micrometers to cubed millimeters. Finally, cell counts were given as cells/cubed millimeters OB. To elucidate the labeling efficiency of the different AAV variants in the total v-SVZ (medial, dorsal, and lateral wall of the lateral ventricle), the cells were counted on 25 μm-thick coronal sections and are given as cells per 25 μm section. Mainly NSCs located in the lateral wall of the ventricle generate OB neurons during homeostasis. Since a particular area of the lateral v-SVZ serves cells to a particular volume of the OB, cell numbers were counted for the mathematical modeling of the lateral v-SVZ only. The length of the lateral ventricular wall was measured in a coronal section and multiplied with the z stack size (25 μm) to estimate the area of the lateral v-SVZ. Afterward, cells in the lateral v-SVZ were counted and normalized to the lateral v-SVZ area. Data are given as cells per cubed millimeters.

NGS screening of barcoded AAV capsid variants—computational analysis

NGS samples were sequenced and demultiplexed by the DKFZ Genomics and Proteomics Core Facility using bcl2fastq 2.19.0.316. This resulted in two (paired-end) FASTQ files per sample. Each FASTQ consists of reads resulting from the targeted barcode amplification and up to 50% PhiX DNA that was spiked in to increase library complexity.

Each AAV variant is associated with a unique 15-mer barcode sequence. To quantify the most successful AAV, we simply counted how often each barcode occurred in each FASTQ file, bearing in mind the following pitfalls:

(1) Barcode sequences might occur outside of the amplicon by chance, e.g., in the PhiX genome.
(2) Barcodes might have sequencing errors.
(3) Barcodes occur on the forward and reverse strand.

To circumvent issues (1) and (2), we opted for a strategy where we only count barcodes matching the expected amplicon structure. This was achieved with the following regex (regular expression; defines a text search pattern): (? ≤ [NGCAT]{33}TGCTC)[NGCAT]{15}(? = CAGGG[NGCAT]{45}). Variable 15-mers [NGCAT]{15} are only counted if they are flanked by the expected regions TGCTC and CAGGG. Furthermore, we enforce a minimum of 33 upstream nt and 15 downstream nt, in addition to the flanking regions, to only count 15-mers at the expected position. 15-mers matching this regex were extracted and counted with the standard GNU command-line tools grep, sort, and uniq. 15-mers sequenced from the reverse strand were counted with an equivalent reverse complement regex and added to the forward counts.

Assigning barcodes to AAV capsids

Raw 15-mer counts were further processed in R. Most observed 15-mers matched a known barcode exactly (library #1: 74%; library #3: 87%), which allowed us to assign them to a unique AAV variant. The remaining 15-mer counts were added to the counts of the closest known barcode, allowing for a maximum of two mismatches.

Normalization

Each sequenced sample corresponds to one tube with up to 500 FACS cells. To downweigh samples with lower cell numbers, barcode counts were scaled by the respective number of FACS events (usually 500; Table S2). Barcode counts of the same cell type and biological replicate (termed “sets”) were then summed. The AAV libraries used for transduction contain slightly unequal proportions of AAV variants, which means that some AAV variants may have an advantage due to increased starting concentration. To remedy this problem, barcode counts were further scaled by their abundance in the transduction library (as determined by Weinmann et al.[53]) (Table S6), so that barcode counts corresponding to more frequent AAV capsids were decreased and vice versa.

To account for sequencing depth of the individual samples, normalized barcode counts were divided by the total number of valid barcodes in that sample, yielding normalized barcode proportions. A potential source of bias is that amplicons with different barcodes may have different RT-PCR efficiencies. A previous study[49] on ten barcoded AAV variants found no such bias, but nonetheless, we evaluated one possible source of bias, barcode GC-content, in our own data. We found no significant association between barcode GC-

content and mean barcode proportion across all samples in either library (Figures S2L and S2M).

Identification of candidate AAVs with high transduction efficiency

To identify the most promising AAV variants, AAVs were ranked by the mean normalized barcode proportion within and across cell types (Figures 1D–1J). AAV1_P5 and AAV9_A2 performed consistently well across replicates of both experiments and were selected for further validation.

Mathematical modeling

A detailed description on how the mathematical modeling was developed is given in Supplemental material.

Statistics

Statistical analyses were performed with R v.4.0.2 using one-way ANOVA followed by Tukey's honest significant difference (HSD) post hoc test unless otherwise noted. Tukey's HSD p values were corrected for multiple testing with the Benjamini-Hochberg procedure. The homogeneity of variance assumption of ANOVA was assessed with Levene's test, and the normality assumption was assessed with the Shapiro-Wilk normality test. The respective p values are indicated in the figure legends. Figures were plotted with the R package ggplot2 and SigmaPlot 12.5.

Data and code availability

All sequencing data are available at the NCBI Gene Expression Omnibus (GEO) under GEO: GSE145172.

All scripts used in the analysis are available at https://github.com/LKremer/AAV-screening.

SUPPLEMENTAL INFORMATION

Supplemental information can be found online at https://doi.org/10.1016/j.omtm.2021.07.001.

ACKNOWLEDGMENTS

We thank Monika Langlotz and the ZMBH FACS Core Facility, DKFZ High Throughput Sequencing Unit, DKFZ Microscopy Core Facility, Ellen Wiedtke and the members of the Dirk Grimm laboratory for technical assistance, and Stefanie Limpert for technical assistance and the members of the Martin-Villalba laboratory for critically reading the manuscript. This work was supported by the German Research Foundation (DFG; SFB873), European Research Council (ERC; REBUILD_CNS), and DKFZ. D.G. kindly acknowledges funding by the DFG: EXC81 (Cluster of Excellence CellNetworks), SFB1129 (Collaborative Research Center 1129, TP2/16, Projektnummer 240245660), and TRR179 (Transregional Collaborative Research Center 179, TP18, Projektnummer 272983813).

AUTHOR CONTRIBUTIONS

S.D. was involved in project and experimental design and performed experiments including *in vitro* and *in vivo* screens, *ex vivo* NSC transplantation, and *in vitro* and *in vivo* validations. L.P.M.K. was responsible for the bioinformatics analysis of all *in vitro* and *in vivo* screens and sequencing experiments. S.K. and S.C. conducted the single-cell RNA sequencing experiment. S.C. conducted the FACS quantification of cells transduced with lead candidates. T.S. was responsible for the mathematical modeling of the *in vivo* data. J.W. provided the two AAV capsid libraries and contributed to experimental design. H.A. and A.L. helped in producing AAV vectors. A.M.-C. contributed to the development of the mathematical model, interpretation of data, and revision of the manuscript. D.G., S.A., A.M.-C., and A.M.-V. supervised the project and wrote the manuscript. A.M.-V. designed and coordinated the study. All authors have read and approved the final version of the manuscript.

DECLARATION OF INTERESTS

D.G. is a co-founder and shareholder of AaviGen GmbH. All other authors declare no competing interests.

REFERENCES

1. Lim, D.A., and Alvarez-Buylla, A. (2016). The Adult Ventricular-Subventricular Zone (V-SVZ) and Olfactory Bulb (OB) Neurogenesis. Cold Spring Harb. Perspect. Biol. *8*, a018820.
2. Ming, G.L., and Song, H. (2011). Adult neurogenesis in the mammalian brain: significant answers and significant questions. Neuron *70*, 687–702.
3. Merkle, F.T., Mirzadeh, Z., and Alvarez-Buylla, A. (2007). Mosaic organization of neural stem cells in the adult brain. Science *317*, 381–384.
4. Fuentealba, L.C., Rompani, S.B., Parraguez, J.I., Obernier, K., Romero, R., Cepko, C.L., and Alvarez-Buylla, A. (2015). Embryonic Origin of Postnatal Neural Stem Cells. Cell *161*, 1644–1655.
5. Merkle, F.T., Fuentealba, L.C., Sanders, T.A., Magno, L., Kessaris, N., and Alvarez-Buylla, A. (2014). Adult neural stem cells in distinct microdomains generate previously unknown interneuron types. Nat. Neurosci. *17*, 207–214.
6. Llorens-Bobadilla, E., Zhao, S., Baser, A., Saiz-Castro, G., Zwadlo, K., and Martin-Villalba, A. (2015). Single-Cell Transcriptomics Reveals a Population of Dormant Neural Stem Cells that Become Activated upon Brain Injury. Cell Stem Cell *17*, 329–340.
7. Arvidsson, A., Collin, T., Kirik, D., Kokaia, Z., and Lindvall, O. (2002). Neuronal replacement from endogenous precursors in the adult brain after stroke. Nat. Med. *8*, 963–970.
8. Parent, J.M., Vexler, Z.S., Gong, C., Derugin, N., and Ferriero, D.M. (2002). Rat forebrain neurogenesis and striatal neuron replacement after focal stroke. Ann. Neurol. *52*, 802–813.
9. Thored, P., Arvidsson, A., Cacci, E., Ahlenius, H., Kallur, T., Darsalia, V., Ekdahl, C.T., Kokaia, Z., and Lindvall, O. (2006). Persistent production of neurons from adult brain stem cells during recovery after stroke. Stem Cells *24*, 739–747.
10. Hou, S.W., Wang, Y.Q., Xu, M., Shen, D.-H., Wang, J.-J., Huang, F., Yu, Z., and Sun, F.-Y. (2008). Functional integration of newly generated neurons into striatum after cerebral ischemia in the adult rat brain. Stroke *39*, 2837–2844.
11. Liu, F., You, Y., Li, X., Ma, T., Nie, Y., Wei, B., Li, T., Lin, H., and Yang, Z. (2009). Brain injury does not alter the intrinsic differentiation potential of adult neuroblasts. J. Neurosci. *29*, 5075–5087.
12. Kalamakis, G., Brüne, D., Ravichandran, S., Bolz, J., Fan, W., Ziebell, F., Stiehl, T., Catalá-Martinez, F., Kupke, J., Zhao, S., et al. (2019). Quiescence Modulates Stem Cell Maintenance and Regenerative Capacity in the Aging Brain. Cell *176*, 1407–1419.e14.
13. Enikolopov, G., Overstreet-Wadiche, L., and Ge, S. (2015). Viral and transgenic reporters and genetic analysis of adult neurogenesis. Cold Spring Harb. Perspect. Biol. *7*, a018804.

14. Thomas, C.E., Ehrhardt, A., and Kay, M.A. (2003). Progress and problems with the use of viral vectors for gene therapy. Nat. Rev. Genet. *4*, 346–358.
15. Li, Z., Düllmann, J., Schiedlmeier, B., Schmidt, M., von Kalle, C., Meyer, J., Forster, M., Stocking, C., Wahlers, A., Frank, O., et al. (2002). Murine leukemia induced by retroviral gene marking. Science *296*, 497.
16. Hacein-Bey-Abina, S., von Kalle, C., Schmidt, M., Le Deist, F., Wulffraat, N., McIntyre, E., Radford, I., Villeval, J.-L., Fraser, C.C., Cavazzana-Calvo, M., and Fischer, A. (2003). A serious adverse event after successful gene therapy for X-linked severe combined immunodeficiency. N. Engl. J. Med. *348*, 255–256.
17. Xia, X., Zhang, Y., Zieth, C.R., and Zhang, S.C. (2007). Transgenes delivered by lentiviral vector are suppressed in human embryonic stem cells in a promoter-dependent manner. Stem Cells Dev. *16*, 167–176.
18. He, J., Yang, Q., and Chang, L.-J. (2005). Dynamic DNA methylation and histone modifications contribute to lentiviral transgene silencing in murine embryonic carcinoma cells. J. Virol. *79*, 13497–13508.
19. Park, F., Ohashi, K., Chiu, W., Naldini, L., and Kay, M.A. (2000). Efficient lentiviral transduction of liver requires cell cycling in vivo. Nat. Genet. *24*, 49–52.
20. Hocquemiller, M., Giersch, L., Audrain, M., Parker, S., and Cartier, N. (2016). Adeno-Associated Virus-Based Gene Therapy for CNS Diseases. Hum. Gene Ther. *27*, 478–496.
21. Deverman, B.E., Ravina, B.M., Bankiewicz, K.S., Paul, S.M., and Sah, D.W.Y. (2018). Gene therapy for neurological disorders: progress and prospects. Nat. Rev. Drug Discov. *17*, 641–659.
22. Foust, K.D., Nurre, E., Montgomery, C.L., Hernandez, A., Chan, C.M., and Kaspar, B.K. (2009). Intravascular AAV9 preferentially targets neonatal neurons and adult astrocytes. Nat. Biotechnol. *27*, 59–65.
23. Wang, D., Tai, P.W.L., and Gao, G. (2019). Adeno-associated virus vector as a platform for gene therapy delivery. Nat. Rev. Drug Discov. *18*, 358–378.
24. Rose, J.A., Berns, K.I., Hoggan, M.D., and Koczot, F.J. (1969). Evidence for a single-stranded adenovirus-associated virus genome: formation of a DNA density hybrid on release of viral DNA. Proc. Natl. Acad. Sci. USA *64*, 863–869.
25. Samulski, R.J., and Muzyczka, N. (2014). AAV-mediated gene therapy for research and therapeutic purposes. Annu. Rev. Virol. *1*, 427–451.
26. Janik, J.E., Huston, M.M., and Rose, J.A. (1984). Adeno-associated virus proteins: origin of the capsid components. J. Virol. *52*, 591–597.
27. Mendelson, E., Trempe, J.P., and Carter, B.J. (1986). Identification of the trans-acting Rep proteins of adeno-associated virus by antibodies to a synthetic oligopeptide. J. Virol. *60*, 823–832.
28. Becerra, S.P., Koczot, F., Fabisch, P., and Rose, J.A. (1988). Synthesis of adeno-associated virus structural proteins requires both alternative mRNA splicing and alternative initiations from a single transcript. J. Virol. *62*, 2745–2754.
29. Trempe, J.P., and Carter, B.J. (1988). Alternate mRNA splicing is required for synthesis of adeno-associated virus VP1 capsid protein. J. Virol. *62*, 3356–3363.
30. Sonntag, F., Schmidt, K., and Kleinschmidt, J.A. (2010). A viral assembly factor promotes AAV2 capsid formation in the nucleolus. Proc. Natl. Acad. Sci. USA *107*, 10220–10225.
31. Johnson, F.B., Ozer, H.L., and Hoggan, M.D. (1971). Structural proteins of adenovirus-associated virus type 3. J. Virol. *8*, 860–863.
32. Van Vliet, K.M., Blouin, V., Brument, N., Agbandje-McKenna, M., and Snyder, R.O. (2008). The role of the adeno-associated virus capsid in gene transfer. Methods Mol. Biol. *437*, 51–91.
33. Li, C., and Samulski, R.J. (2020). Engineering adeno-associated virus vectors for gene therapy. Nat. Rev. Genet. *21*, 255–272.
34. Daya, S., and Berns, K.I. (2008). Gene therapy using adeno-associated virus vectors. Clin. Microbiol. Rev. *21*, 583–593.
35. Xiao, X., Li, J., and Samulski, R.J. (1996). Efficient long-term gene transfer into muscle tissue of immunocompetent mice by adeno-associated virus vector. J. Virol. *70*, 8098–8108.
36. Naso, M.F., Tomkowicz, B., Perry, W.L., 3rd, and Strohl, W.R. (2017). Adeno-Associated Virus (AAV) as a Vector for Gene Therapy. BioDrugs *31*, 317–334.
37. Lykken, E.A., Shyng, C., Edwards, R.J., Rozenberg, A., and Gray, S.J. (2018). Recent progress and considerations for AAV gene therapies targeting the central nervous system. J. Neurodev. Disord. *10*, 16.
38. Colella, P., Ronzitti, G., and Mingozzi, F. (2017). Emerging Issues in AAV-Mediated *In Vivo* Gene Therapy. Mol. Ther. Methods Clin. Dev. *8*, 87–104.
39. Cearley, C.N., and Wolfe, J.H. (2006). Transduction characteristics of adeno-associated virus vectors expressing cap serotypes 7, 8, 9, and Rh10 in the mouse brain. Mol. Ther. *13*, 528–537.
40. Gray, S.J., Nagabhushan Kalburgi, S., McCown, T.J., and Jude Samulski, R. (2013). Global CNS gene delivery and evasion of anti-AAV-neutralizing antibodies by intrathecal AAV administration in non-human primates. Gene Ther. *20*, 450–459.
41. Gray, S.J., Matagne, V., Bachaboina, L., Yadav, S., Ojeda, S.R., and Samulski, R.J. (2011). Preclinical differences of intravascular AAV9 delivery to neurons and glia: a comparative study of adult mice and nonhuman primates. Mol. Ther. *19*, 1058–1069.
42. Ojala, D.S., Sun, S., Santiago-Ortiz, J.L., Shapiro, M.G., Romero, P.A., and Schaffer, D.V. (2018). In Vivo Selection of a Computationally Designed SCHEMA AAV Library Yields a Novel Variant for Infection of Adult Neural Stem Cells in the SVZ. Mol. Ther. *26*, 304–319.
43. Brown, N., Song, L., Kollu, N.R., and Hirsch, M.L. (2017). Adeno-Associated Virus Vectors and Stem Cells: Friends or Foes? Hum. Gene Ther. *28*, 450–463.
44. Kienle, E., Senís, E., Börner, K., Niopek, D., Wiedtke, E., Grosse, S., and Grimm, D. (2012). Engineering and evolution of synthetic adeno-associated virus (AAV) gene therapy vectors via DNA family shuffling. J. Vis. Exp. 3819.
45. Srivastava, A. (2016). Adeno-Associated Virus: The Naturally Occurring Virus Versus the Recombinant Vector. Hum. Gene Ther. *27*, 1–6.
46. Körbelin, J., and Trepel, M. (2017). How to Successfully Screen Random Adeno-Associated Virus Display Peptide Libraries In Vivo. Hum. Gene Ther. Methods *28*, 109–123.
47. Büning, H., and Srivastava, A. (2019). Capsid Modifications for Targeting and Improving the Efficacy of AAV Vectors. Mol. Ther. Methods Clin. Dev. *12*, 248–265.
48. Deverman, B.E., Pravdo, P.L., Simpson, B.P., Kumar, S.R., Chan, K.Y., Banerjee, A., Wu, W.-L., Yang, B., Huber, N., Pasca, S.P., and Gradinaru, V. (2016). Cre-dependent selection yields AAV variants for widespread gene transfer to the adult brain. Nat. Biotechnol. *34*, 204–209.
49. Herrmann, A.K., Bender, C., Kienle, E., Grosse, S., El Andari, J., Botta, J., Schürmann, N., Wiedtke, E., Niopek, D., and Grimm, D. (2019). A Robust and All-Inclusive Pipeline for Shuffling of Adeno-Associated Viruses. ACS Synth. Biol. *8*, 194–206.
50. Grimm, D., and Zolotukhin, S. (2015). E Pluribus Unum: 50 Years of Research, Millions of Viruses, and One Goal–Tailored Acceleration of AAV Evolution. Mol. Ther. *23*, 1819–1831.
51. Adachi, K., Enoki, T., Kawano, Y., Veraz, M., and Nakai, H. (2014). Drawing a high-resolution functional map of adeno-associated virus capsid by massively parallel sequencing. Nat. Commun. *5*, 3075.
52. Marsic, D., Méndez-Gómez, H.R., and Zolotukhin, S. (2015). High-accuracy biodistribution analysis of adeno-associated virus variants by double barcode sequencing. Mol. Ther. Methods Clin. Dev. *2*, 15041.
53. Weinmann, J., Weis, S., Sippel, J., Tulalamba, W., Remes, A., El Andari, J., Herrmann, A.-K., Pham, Q.H., Borowski, C., Hille, S., et al. (2020). Identification of a myotropic AAV by massively parallel in vivo evaluation of barcoded capsid variants. Nat. Commun. *11*, 5432.
54. Codega, P., Silva-Vargas, V., Paul, A., Maldonado-Soto, A.R., Deleo, A.M., Pastrana, E., and Doetsch, F. (2014). Prospective identification and purification of quiescent adult neural stem cells from their in vivo niche. Neuron *82*, 545–559.
55. Szczurkowska, J., Cwetsch, A.W., dal Maschio, M., Ghezzi, D., Ratto, G.M., and Cancedda, L. (2016). Targeted in vivo genetic manipulation of the mouse or rat brain by in utero electroporation with a triple-electrode probe. Nat. Protoc. *11*, 399–412.
56. Federici, T., Taub, J.S., Baum, G.R., Gray, S.J., Grieger, J.C., Matthews, K.A., Handy, C.R., Passini, M.A., Samulski, R.J., and Boulis, N.M. (2012). Robust spinal motor neuron transduction following intrathecal delivery of AAV9 in pigs. Gene Ther. *19*, 852–859.

57. Mirzadeh, Z., Merkle, F.T., Soriano-Navarro, M., Garcia-Verdugo, J.M., and Alvarez-Buylla, A. (2008). Neural stem cells confer unique pinwheel architecture to the ventricular surface in neurogenic regions of the adult brain. Cell Stem Cell *3*, 265–278.

58. Shah, P.T., Stratton, J.A., Stykel, M.G., Abbasi, S., Sharma, S., Mayr, K.A., Koblinger, K., Whelan, P.J., and Biernaskie, J. (2018). Single-Cell Transcriptomics and Fate Mapping of Ependymal Cells Reveals an Absence of Neural Stem Cell Function. Cell *173*, 1045–1057.e9.

59. Qiao, C., Yuan, Z., Li, J., He, B., Zheng, H., Mayer, C., Li, J., and Xiao, X. (2011). Liver-specific microRNA-122 target sequences incorporated in AAV vectors efficiently inhibits transgene expression in the liver. Gene Ther. *18*, 403–410.

60. Geisler, A., and Fechner, H. (2016). MicroRNA-regulated viral vectors for gene therapy. World J. Exp. Med. *6*, 37–54.

61. Baser, A., Skabkin, M., Kleber, S., Dang, Y., Gülcüler Balta, G.S., Kalamakis, G., Göpferich, M., Ibañez, D.C., Schefzik, R., Lopez, A.S., et al. (2019). Onset of differentiation is post-transcriptionally controlled in adult neural stem cells. Nature *566*, 100–104.

62. Niu, W., Zou, Y., Shen, C., and Zhang, C.L. (2011). Activation of postnatal neural stem cells requires nuclear receptor TLX. J. Neurosci. *31*, 13816–13828.

63. Svensson, V. (2020). Droplet scRNA-seq is not zero-inflated. Nat. Biotechnol. *38*, 147–150.

64. Kotterman, M.A., Vazin, T., and Schaffer, D.V. (2015). Enhanced selective gene delivery to neural stem cells in vivo by an adeno-associated viral variant. Development *142*, 1885–1892.

65. Crowther, A.J., Lim, S.-A., Asrican, B., Albright, B.H., Wooten, J., Yeh, C.-Y., Bao, H., Cerri, D.H., Hu, J., Ian Shih, Y.-Y., et al. (2018). An Adeno-Associated Virus-Based Toolkit for Preferential Targeting and Manipulating Quiescent Neural Stem Cells in the Adult Hippocampus. Stem Cell Reports *10*, 1146–1159.

66. Sallach, J., Di Pasquale, G., Larcher, F., Niehoff, N., Rübsam, M., Huber, A., Chiorini, J., Almarza, D., Eming, S.A., Ulus, H., et al. (2014). Tropism-modified AAV vectors overcome barriers to successful cutaneous therapy. Mol. Ther. *22*, 929–939.

67. Hordeaux, J., Yuan, Y., Clark, P.M., Wang, Q., Martino, R.A., Sims, J.J., Bell, P., Raymond, A., Stanford, W.L., and Wilson, J.M. (2019). The GPI-Linked Protein LY6A Drives AAV-PHP.B Transport across the Blood-Brain Barrier. Mol. Ther. *27*, 912–921.

68. Matsuzaki, Y., Tanaka, M., Hakoda, S., Masuda, T., Miyata, R., Konno, A., and Hirai, H. (2019). Neurotropic Properties of AAV-PHP.B Are Shared among Diverse Inbred Strains of Mice. Mol. Ther. *27*, 700–704.

69. Vandenberghe, L.H. (2019). AAV Engineering Identifies a Species Barrier That Highlights a Portal to the Brain. Mol. Ther. *27*, 901–903.

70. Pillay, S., Meyer, N.L., Puschnik, A.S., Davulcu, O., Diep, J., Ishikawa, Y., Jae, L.T., Wosen, J.E., Nagamine, C.M., Chapman, M.S., and Carette, J.E. (2016). An essential receptor for adeno-associated virus infection. Nature *530*, 108–112.

71. Zhong, L., Li, B., Mah, C.S., Govindasamy, L., Agbandje-McKenna, M., Cooper, M., Herzog, R.W., Zolotukhin, I., Warrington, K.H., Jr., Weigel-Van Aken, K.A., et al. (2008). Next generation of adeno-associated virus 2 vectors: point mutations in tyrosines lead to high-efficiency transduction at lower doses. Proc. Natl. Acad. Sci. USA *105*, 7827–7832.

72. Zywitza, V., Misios, A., Bunatyan, L., Willnow, T.E., and Rajewsky, N. (2018). Single-cell transcriptomics characterizes cell types in the subventricular zone and uncovers molecular defects impairing adult neurogenesis. Cell Rep. *25*, 2457–2469.e8.

73. Alemany, A., Florescu, M., Baron, C.S., Peterson-Maduro, J., and van Oudenaarden, A. (2018). Whole-organism clone tracing using single-cell sequencing. Nature *556*, 108–112.

74. Raj, B., Wagner, D.E., McKenna, A., Pandey, S., Klein, A.M., Shendure, J., Gagnon, J.A., and Schier, A.F. (2018). Simultaneous single-cell profiling of lineages and cell types in the vertebrate brain. Nat. Biotechnol. *36*, 442–450.

75. Liu, Y., Okada, T., Nomoto, T., Ke, X., Kume, A., Ozawa, K., and Xiao, S. (2007). Promoter effects of adeno-associated viral vector for transgene expression in the cochlea in vivo. Exp. Mol. Med. *39*, 170–175.

76. Damdindorj, L., Karnan, S., Ota, A., Hossain, E., Konishi, Y., Hosokawa, Y., and Konishi, H. (2014). A comparative analysis of constitutive promoters located in adeno-associated viral vectors. PLoS ONE *9*, e106472.

77. Börner, K., Niopek, D., Cotugno, G., Kaldenbach, M., Pankert, T., Willemsen, J., Zhang, X., Schürmann, N., Mockenhaupt, S., Serva, A., et al. (2013). Robust RNAi enhancement via human Argonaute-2 overexpression from plasmids, viral vectors and cell lines. Nucleic Acids Res. *41*, e199.

78. Sarcar, S., Tulalamba, W., Rincon, M.Y., Tipanee, J., Pham, H.Q., Evens, H., Boon, D., Samara-Kuko, E., Keyaerts, M., Loperfido, M., et al. (2019). Next-generation muscle-directed gene therapy by in silico vector design. Nat. Commun. *10*, 492.

79. Börner, K., Kienle, E., Huang, L.-Y., Weinmann, J., Sacher, A., Bayer, P., Stüllein, C., Fakhiri, J., Zimmermann, L., Westhaus, A., et al. (2020). Pre-arrayed Pan-AAV Peptide Display Libraries for Rapid Single-Round Screening. Mol. Ther. *28*, 1016–1032.

80. Senís, E., Mosteiro, L., Wilkening, S., Wiedtke, E., Nowrouzi, A., Afzal, S., Fronza, R., Landerer, H., Abad, M., Niopek, D., et al. (2018). AAVvector-mediated in vivo reprogramming into pluripotency. Nat. Commun. *9*, 2651.

81. Mirzadeh, Z., Doetsch, F., Sawamoto, K., Wichterle, H., and Alvarez-Buylla, A. (2010). The subventricular zone en-face: wholemount staining and ependymal flow. J. Vis. Exp. 1938.

82. Walker, T.L., and Kempermann, G. (2014). One mouse, two cultures: isolation and culture of adult neural stem cells from the two neurogenic zones of individual mice. J. Vis. Exp. e51225.

83. Bray, N.L., Pimentel, H., Melsted, P., and Pachter, L. (2016). Near-optimal probabilistic RNA-seq quantification. Nat. Biotechnol. *34*, 525–527.

84. Melsted, P., Ntranos, V., and Pachter, L. (2019). The barcode, UMI, set format and BUStools. Bioinformatics *35*, 4472–4473.

85. Wolf, F.A., Angerer, P., and Theis, F.J. (2018). SCANPY: large-scale single-cell gene expression data analysis. Genome Biol. *19*, 15.

86. Tirosh, I., Izar, B., Prakadan, S.M., Wadsworth, M.H., 2nd, Treacy, D., Trombetta, J.J., Rotem, A., Rodman, C., Lian, C., Murphy, G., et al. (2016). Dissecting the multicellular ecosystem of metastatic melanoma by single-cell RNA-seq. Science *352*, 189–196.

87. Love, M.I., Huber, W., and Anders, S. (2014). Moderated estimation of fold change and dispersion for RNA-seq data with DESeq2. Genome Biol. *15*, 550.

88. Stuart, T., Butler, A., Hoffman, P., Hafemeister, C., Papalexi, E., Mauck, W.M., 3rd, Hao, Y., Stoeckius, M., Smibert, P., and Satija, R. (2019). Comprehensive Integration of Single-Cell Data. Cell *177*, 1888–1902.e21.

89. Picelli, S., Faridani, O.R., Björklund, A.K., Winberg, G., Sagasser, S., and Sandberg, R. (2014). Full-length RNA-seq from single cells using Smart-seq2. Nat. Protoc. *9*, 171–181.

OMTM, Volume *23*

Supplemental information

High throughput screening of novel AAV capsids identifies variants for transduction of adult NSCs within the subventricular zone

Lukas P.M. Kremer, Santiago Cerrizuela, Sascha Dehler, Thomas Stiehl, Jonas Weinmann, Heike Abendroth, Susanne Kleber, Alexander Laure, Jihad El Andari, Simon Anders, Anna Marciniak-Czochra, Dirk Grimm, and Ana Martin-Villalba

Data S1. Mathematical Modeling of Labeling Dynamics with AAV1 P5 transduced v-SVZ cells

Mathematical Modeling of Labeling Dynamics with AAV1_P5 transduced v-SVZ cells

1 Mathematical Model of Neurogenesis

We extend our previously established mathematical model from [1–3]. The model describes time evolution of active NSC, quiescent NSC and TAPs. The model considers the following processes:

- Quiescent stem cells are activated at the rate r. As demonstrated in [1] the activation rate depends on the age of the organism.
- Division of active stem cells occurs at the rate p_{stem}. Upon division a stem cell gives rise to two progeny.
- The probability that a progeny is a stem cell is b. It is referred to as self-renewal probability. With probability $(1 - b)$ the progeny differentiates into a TAP.
- TAPs divide a finite number of times before they further differentiate.

The part of the model describing stem cell dynamics has been parameterized in [1]. The TAP dynamics have not been calibrated so far. We assume a TAP doubling time of 20.15 hours taken from [4]. Assuming 4 TAP divisions we obtain the best agreement of model simulations with the TAP data from [1], see Figure 1. This number of TAP divisions is in agreement with the measurements from [4]. We then obtain the following model.

$$
\begin{aligned}
\frac{d}{dt}qNSC =& - r(t) \cdot qNSC + 2 \cdot b \cdot p_{stem} aNSC \\
\frac{d}{dt}aNSC =& r(t) \cdot qNSC - p_{stem} \cdot aNSC \\
\frac{d}{dt}TAP_0 =& - p_{prog} \cdot TAP_0 + 2 \cdot (1-b) \cdot p_{stem} \cdot aNSC \\
\frac{d}{dt}TAP_1 =& - p_{TAP} \cdot TAP_1 + 2 \cdot p_{TAP} \cdot TAP_0 \\
\frac{d}{dt}TAP_2 =& - p_{TAP} \cdot TAP_2 + 2 \cdot p_{TAP} \cdot TAP_1 \\
\frac{d}{dt}TAP_3 =& - p_{TAP} \cdot TAP_3 + 2 \cdot p_{TAP} \cdot TAP_2 \\
r(t) =& r_{max} \exp(-\beta_r t)
\end{aligned}
\tag{1}
$$

As $qNSC(t)$ and $aNSC(t)$ we denote the amount of quiescent and active neural stem cells at time t. As $TAP_i(t)$, $i \in \{0,\ 1,\ 2,\ 3\}$ we denote the amount of TAPs that have performed i divisions at time t. Namely, $aNSC$ give rise to TAP_0. If TAP_0 divide, the progeny belong to TAP_1, i.e., TAP that have performed one division. Analogously TAP_1 give rise to TAP_2, TAP_2 give rise to TAP_3 and progeny of TAP_3 are neuroblasts. For notational convenience we omit the argument t and identify $qNSC(t) \equiv qNSC$, $aNSC(t) \equiv aNSC$, $TAP_i(t) \equiv TAP_i$. Proliferation rates of stem cells and TAP are denoted as p_{stem} and p_{TAP} respectively. By b we denote the probability of stem cell self-renewal. It is the probability with which a progeny of a stem cell is again a stem cell [5–7]. We note that the initial condition for TAPs has practically no impact on the cell counts at ages larger than 1 month. All model parameters are summarized in Table 1.

2 Modeling of Labeling Dynamics

The serotype is injected in mice of age $\tau = 56$ days. We denote the time of serotype injection as $t = 0$.

We make the following assumptions

- The transduced cell expresses a fluorescent label that is transmitted over the cell division. We assume that proliferation rates and self-renewal probability are not affected by the virus.

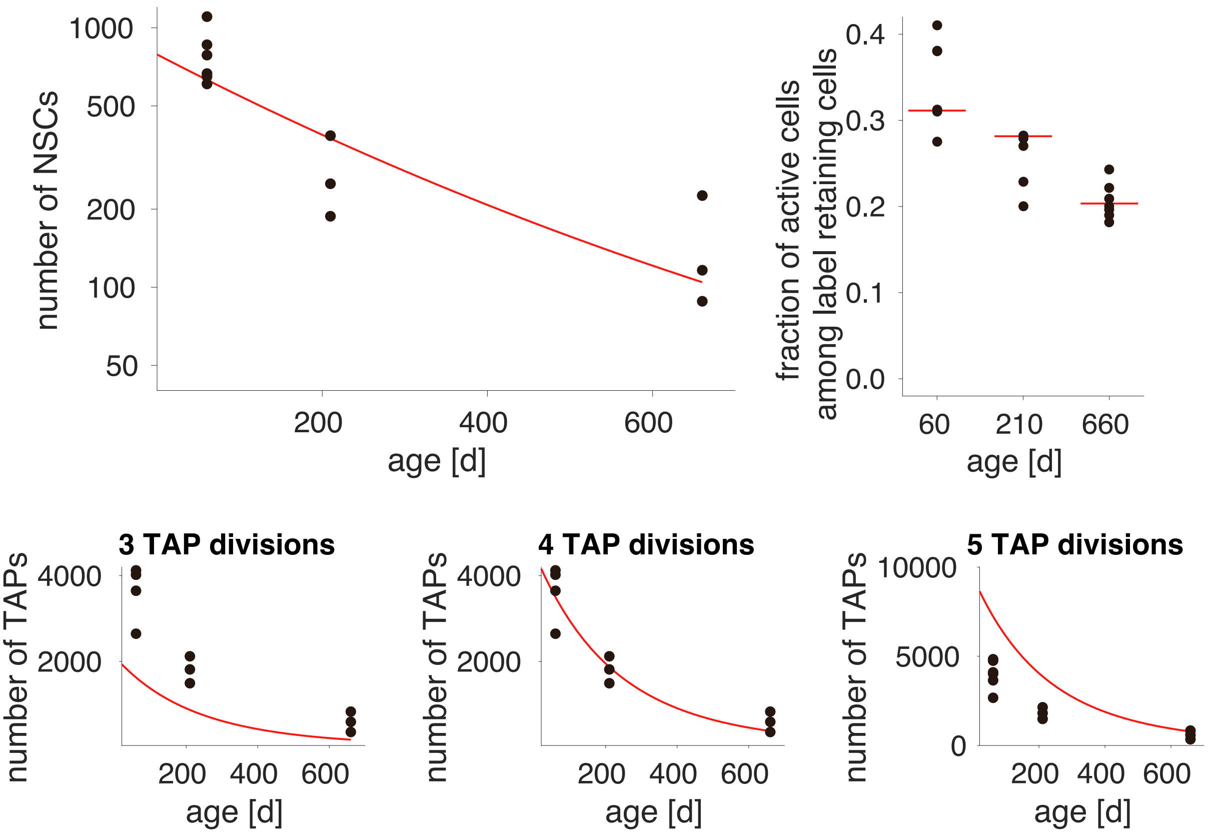

Figure 1: Simulation of NSC and TAP dynamics during aging. Upper: NSC dynamics. Lower: TAP dynamics assuming 3, 4 or 5 TAP divisions before further differentiation. We obtain the best agreement of model and data for 4 TAP divisions. Red curve: Model simulations, black dots: data from [1]. The figures are obtained from simulation of model (1) with the parameters specified in Table 1.

parameter	value
r_{max}	$0.453\ d^{-1}$
β_r	$9.5 \cdot 10^{-4}\ d^{-1}$
b	0.494
p_{stem}	$0.951\ d^{-1}$
p_{TAP}	$0.826\ d^{-1}$
$qNSC(0)$	535
aNSC(0)	255

Table 1: Parameters of the neurogenesis model. Parameters are taken from [1].

- The probability for a single TAP to be labeled is the same for TAP_0, TAP_1, TAP_2, and TAP_3.
- After the fourth division a TAP gives rise to two neuroblasts.
- Neuroblasts arrive after a delay θ in the olfactory bulb. During migration neuroblasts can divide and die. For this reason we assume that the amount of neuroblasts arriving in the olfactory bulb at time t is $\mu \cdot NB(t-\theta)$. If proliferation outweights death $\mu > 1$, otherwise $\mu < 1$. The factor μ also takes into account the different volumes of SVZ and OB.
- We neglect the death of labeled OB cells during the duration of the experiment.

Dynamics of labeled cells is given by the system of equations (1) supplemented by the following equation for olfactory bulb neurons

$$\frac{d}{dt}OB = \mu \cdot p_{TAP} \cdot TAP_3(t-\theta) \tag{2}$$

and the initial condition

$$\begin{aligned} qNSC(0) &= qNSC_0 \\ aNSC(0) &= aNSC_0 \\ TAP_i(0) &= TAP_{i,0},\ i \in \{0,\ 1,\ 2,\ 3\} \\ OB(0) &= 0. \end{aligned} \tag{3}$$

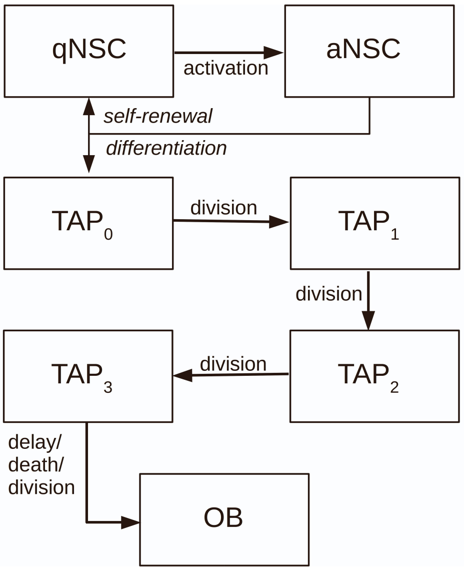

Figure 2: Model of serotype labeling. The scheme depicts the processes described by system (1)-(4). $qNSC$: labeled quiescent NSC, $aNSC$: labeled active NSC, TAP_i: labeled TAPs that have performed i divisions, OB: labeled cells in the olfactory bulb.

The age of the mice at the beginning of the experiment is $\tau = 56d$. Since we define the time when the experiment starts as $t = 0$, the equation for r is given by

$$r(t) = r_{max} \exp(-\beta_r(t + \tau)). \tag{4}$$

The model is visualized in Figure 2. In agreement with the quasi-steady state cell counts we set $TAP_{i,0} = 2 \cdot TAP_{i-1,0}$ for $i \in \{1, 2, 3\}$. For $t < 0$ all populations equal 0.

3 Data

We consider densities of labeled NSC (given per mm^2 of SVZ) and labeled olfactory bulb neurons (given per mm^3 of olfactory bulb). The data shows high heterogeneity among individual mice, with more than an order of magnitude between individual measurements.

Instead of fitting the model to average cell counts we subdivide the data into two groups. The data and the subgroups are shown in Figure 3. We ask whether we can fit the data of

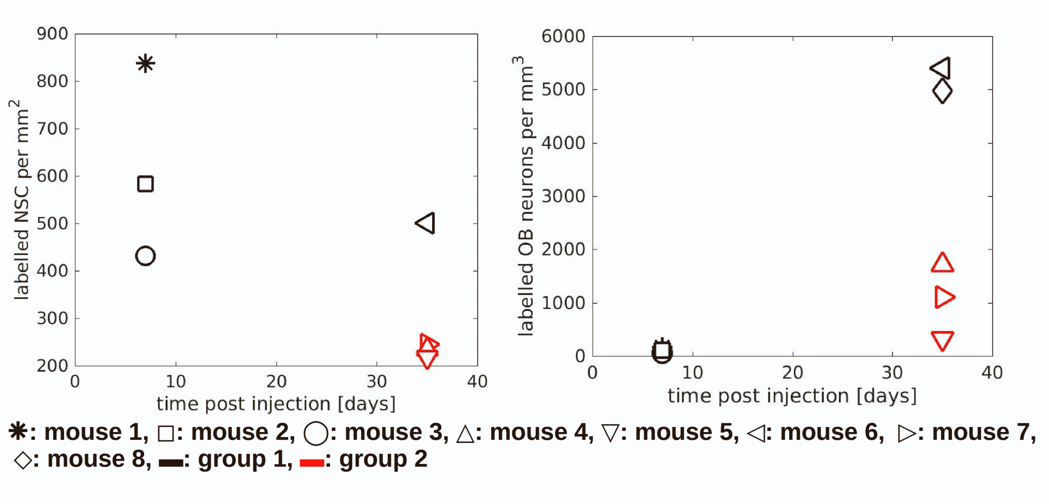

Figure 3: Time dynamics of labeled cells. Each mouse is identified by one symbol. Due to the heterogeneity among individual mice, each mouse was assigned to one of two groups. The color of the symbols indicates to which group the respective mouse belongs.

both groups assuming that they differ only with respect to the number of initially labeled NSC and TAP.

Taking into account the heterogeneity among mice the data was assigned to two different groups as follows:

- The data at day 35 was subdivided into one group of mice showing high numbers of labeled cells (group 1) and one group of mice showing low numbers of labeled cells (group 2).
- The mice studied at day 7 show less heterogeneity, therefore they are not subdivided.
- Taking into account that stem cell numbers practically do not vary in a time interval of 28 days [1], we assign the data points acquired at day 7 to group 1. Assignment of this data to group 2 leads to a worse fit.

4 Fitting

We use densities of labeled primitive cells and labeled of OB cells.

We assume:

- Counts of labeled primitive cells in the SVZ correspond to the sum of labeled aNSC and labeled qNSC.
- Labelled TAPs exist in the model but they do not contribute to the experimentally counted labeled primitive cells in the SVZ.

We assume that r_{max}, β_r, b, p_{stem} and p_{TAP} are not affected by the labeling and assume the values given in Table 1. These values are taken from [1]. We assume that μ and θ do not vary between group one and group two. The number of initially labeled cells, $qNSC_0$, $aNSC_0$, $\sum_{i=0}^{3} TAP_{i,0}$ may be different for both groups. We estimate the unknown parameters using fmincon from MATLAB (The MatWorks, Natic, USA). The cost functional passed to fmincon is a weighted least square functional with the inverse of the standard deviations as weights. We assume that the standard deviation of the labeled NSC count in group 1 at 35 days is equal to the standard deviation of the labeled NSC count in group 2 at 35 days. We choose a multi-start approach with Latin hypercube sampling.

Our stem cell data is given per mm^2 of SVZ. Since there exist around 1000 NSC per mm^2 [8], we set an upper bound of 1000 for the initial number of labeled NSC.

The data from [1] imply that there exist approximately 5000 TAPs per 1000 NSC (standard deviation 1000) at the age where the experiments start. For the fitting we set an upper bound of 7000 TAPs per 1000 NSC.

According to the model in [1] at an age of 56 days approx. 69% of the NSC are quiescent and 31% are active. We assume that active and quiescent NSC are labeled with the same probability. Assuming different labeling probabilities for active and quiescent cells increases the number of free parameters and leads to a worse AIC_c value ($\Delta AIC_c > 10$).

The fitted parameters are provided in Table 2. The fit is depicted in Figure 4. Assuming that the labeling does not affect cell kinetics and that the observed heterogeneity comes from different numbers of initially labeled NSC and TAP, we obtain that in group 1 approx. 57% of the NSC are labeled. This corresponds to approximately 393 qNSC per mm^2 and 177

parameter	value
$aNSC_0 + qNSC_0$	group 1: 570.6, group 2: 261.8
$\sum_{i=0}^{3} TAP_{i,0}$	group 1: 7000, group 2: 0.4
θ	6.7d
μ	0.1m

Table 2: Parameters obtained from the fit of the model to the experimental data. The model was fit to data from both groups simultaneously Only the number of initially labeled cells was allowed to be different for both groups. Other parameters are taken from [1].

aNSC per mm^2. In group 2 approximately 26% of NSC are labeled. This corresponds to approximately 179 qNSC per mm^2 and 81 aNSC per mm^2.

In the model we neglect the time between transduction and label expression. Shortly after the beginning of the experiment the number of experimentally detected labeled cells may differ from the labeled cell counts predicted by the model, since the cells transduced at day zero and counted as labelled cells in the model may not yet express sufficient label concentrations to be detected.

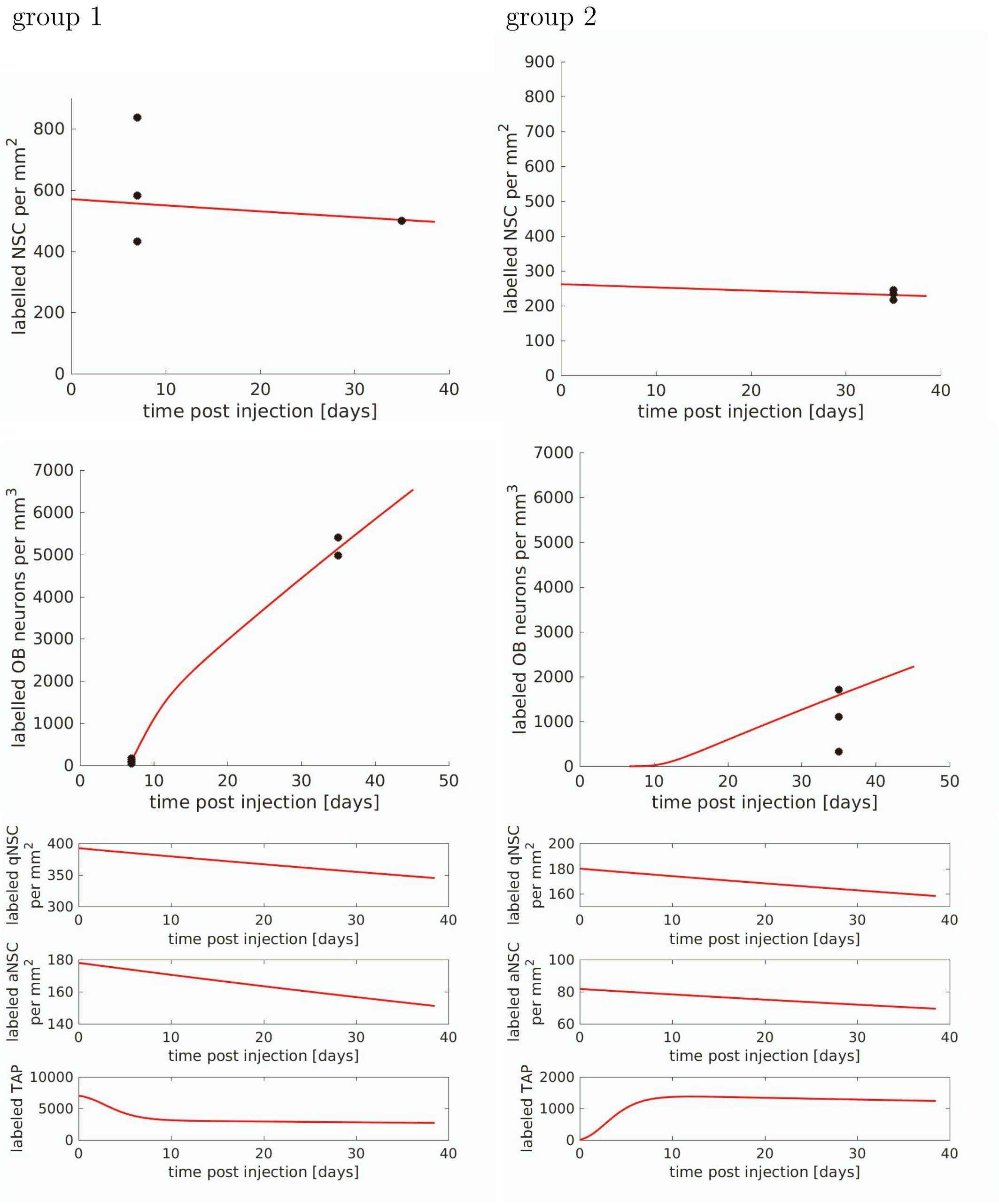

Figure 4: Comparison of model fit and data. The left column compares the fit to data from group 1, the right column to data from group 2. The model was fit to both groups simultaneously. Only the number of initially labeled NSC and TAP differs between the groups.

References

[1] Kalamakis G, Brüne D, Ravichandran S, Bolz J, Fan W, Ziebell F, Stiehl T, Catala-Martinez F, Kupke J, Zhao S, Llorens-Bobadilla E, Bauer K, Limpert S, Berger B, Christen U, Schmezer P, Mallm JP, Berninger B, Anders S, Del Sol A, Marciniak-Czochra A, Martin-Villalba A. Quiescence Modulates Stem Cell Maintenance and Regenerative Capacity in the Aging Brain. *Cell.* 2019, 176(6):1407-1419.

[2] Ziebell F, Martin-Villalba A, Marciniak-Czochra A. Mathematical modelling of adult hippocampal neurogenesis: effects of altered stem cell dynamics on cell counts and bromodeoxyuridine-labelled cells. *J R Soc Interface.* 2014, 11(94):20140144.

[3] Ziebell F, Dehler S, Martin-Villalba A, Marciniak-Czochra A. Revealing age-related changes of adult hippocampal neurogenesis using mathematical models. Development. 2018, 145(1). pii: dev153544.

[4] Ponti G, Obernier K, Guinto C, Jose L, Bonfanti L, Alvarez-Buylla A. Cell cycle and lineage progression of neural progenitors in the ventricular-subventricular zones of adult mice. *Proc Natl Acad Sci U S A.* 2013, 110(11):E1045-54.

[5] Marciniak-Czochra A, Stiehl T, Ho AD, Jäger W, Wagner W. Modeling of asymmetric cell division in hematopoietic stem cells–regulation of self-renewal is essential for efficient repopulation.*Stem Cells Dev.* 2009, 18(3):377-85.

[6] Stiehl T, Marciniak-Czochra A. Characterization of stem cells using mathematical models of multistage cell lineages. *Math Comput Modeling.* 2011, 53(7-8): 1505-1517.

[7] Stiehl T, Marciniak-Czochra A. Stem cell self-renewal in regeneration and cancer: Insights from mathematical modeling. *Curr Opinion Systems Biology.* 2018, 5: 112-120.

[8] Shook BA, Manz DH, Peters JJ, Kang S, Conover JC. Spatiotemporal changes to the subventricular zone stem cell pool through aging. *J Neurosci.* 2012, 32(20):6947-56.

Supplemental Material

Supplemental tables

Supplementary Table 1: Primers and probes used for PCR/qPCR

**Name Primer/Probe**	**Sequence**
Bar_fwd	5'-ATCACTCTCGGCATGGACGAGC-3'
Bar_rev	5'-GGCTGGCAACTAGAAGGCACA-3'
Cre_fwd	5'-ACTGACGGTGGGAGAATGTT-3'
Cre_probe	5' FAM-ACCTGCGGTGCTAACCAGCGT-BHQ1 3'
Cre_rev	5'-CCAGGCTAAGTGCCTTCTCT-3'
GFP_fwd	5'-GAGCGCACCATCTTCTTCAAG-3'
GFP_probe	5' FAM-ACGACGGCAACTACA-BHQ1 3'
GFP_rev	5'-TGTCGCCCTCGAACTTCAC-3'
ISPCR oligo	5'-AAGCAGTGGTATCAACGCAGAGT-3'
Locked Nucleic Acid-TSO	5'-iCiGiCAAGCAGTGGTATCAACGCAGAGTACATrGrG{G}-3'
Oligo-dT	5'-AAGCAGTGGTATCAACGCAGAGTACT$_{30}$VN-3'

Supplementary Table 2: Sequenced multiplex after Library1 transduction

	Celltype	Set	Sample Number	Total Cell Number	Cycle number
Library 1	qNSCs	Set1	1	443	15
Library 1	aNSCs	Set1	1	500	15
			2	500	15
Library 1	TAPs	Set1	1	500	15
			2	500	15
			3	400	15
Library 1	Neuroblasts	Set1	1	500	15
			2	500	15
			3	500	15
Library 1	qNSCs	Set 2	1	500	15
			2	500	15
			3	500	15
			4	500	15
			5	500	15
Library 1	aNSCs	Set 2	1	500	15
			2	500	15
			3	500	15
			4	500	15
			5	500	15
			6	500	15
Library 1	TAPs	Set 2	1	500	15
			2	500	15
			3	500	15
			4	500	15
Library 1	Neuroblasts	Set 2	1	500	15
			2	500	15
			3	500	15
			4	500	15
			5	500	15
Library 1	qNSCs	Set 3	1	500	15
			2	500	15
			3	500	15
Library 1	aNSCs	Set 3	1	500	15
			2	500	15
			3	500	15
			4	500	15
			5	500	15
Library 1	TAPs	Set 3	1	500	15
			2	500	15
			3	500	15
Library 1	Neuroblasts	Set 3	1	500	15
			2	500	15
			3	500	15
			4	500	15
			5	500	15
Library 1	Astrocytes	Set 4	1	500	15
			2	500	15
			3	500	15
			4	500	15
			5	500	15
Library 1	Oligodendrocytes	Set 4	1	500	15
			2	500	15
			3	500	15
			4	500	15
			5	500	15
Library 1	Ependymal cells	Set 4	1	500	15
Library 1	Astrocytes	Set 5	1	500	15
			2	500	15
			3	500	15
			4	500	15
			5	500	15
			6	500	15
Library 1	Oligodendrocytes	Set 5	1	500	15
			2	500	15
			3	500	15
			4	500	15
			5	500	15
			6	500	15
Library 1	Ependymal cells	Set 5	1	125	16
Library 1	Astrocytes	Set 6	1	500	15
			2	500	15
			3	500	15
			4	500	15
Library 1	Oligodendrocytes	Set 6	1	500	15
			2	500	15
			3	500	15
			4	268	16
Library 1	Ependymalcells	Set 6	1	199	16
Library 1	In vitro cultured NSCs	Set1	1	1500	14
Library 1	In vitro cultured NSCs	Set2	1	1500	14

Supplementary Table 3: Sequenced multiplex after Library3 transduction

	Celltype	Set	Batch Number	Cell Numbers	Cycle number
Library 3	qNSCs	Set1	1	500	15
			2	474	15
Library 3	aNSCs	Set1	1	500	15
			2	500	15
			3	380	15
Library 3	TAPs	Set1	1	500	15
Library 3	Neuroblasts	Set1	1	500	15
			2	500	15
			3	500	15
Library 3	qNSCs	Set2	1	500	15
			2	369	15
Library 3	aNSC	Set2	1	500	15
			2	500	15
			3	500	15
			4	345	15
Library 3	qNSC	Set3	1	500	15
			2	500	15
			3	415	15
Library 3	aNSCs	Set3	1	500	15
			2	500	15
			3	500	15
			4	376	15
Library 3	TAPs	Set3	1	500	15
			2	500	15
			3	500	15
			4	500	15
			5	500	15
Library 3	Neuroblasts	Set3	1	500	15
			2	500	15
			3	500	15
			4	500	15
			5	500	15
Library 3	Astrocytes	Set4	1	500	15
			2	500	15
			3	500	15
			4	500	15
			5	500	15
Library 3	Oligodendrocytes	Set4	1	500	15
			2	500	15
			3	500	15
			4	500	15
			5	500	15
Library 3	Ependymal cells	Set4	1	500	15
			2	500	15
			3	339	15
Library 3	Astrocytes	Set5	1	500	15
			2	500	15
			3	500	15
			4	500	15
			5	500	15
Library 3	Oligodendrocytes	Set5	1	500	15
			2	500	15
			3	500	15
			4	500	15
			5	500	15
Library 3	Ependymal cells	Set5	1	450	15
Library 3	Astrocytes	Set6	1	500	15
			2	500	15
			3	500	15
			4	500	15
			5	500	15
Library 3	Oligodendrocytes	Set6	1	500	15
			2	500	15
			3	500	15
			4	500	15
			5	500	15
Library 3	Ependymal cells	Set6	1	210	16
Library 3	In vitro cultured NSCs	Set1	1	1500	14
Library 3	In vitro cultured NSCs	Set1	1	1500	14

Supplementary Table S4: AAV variants used in library #1 and library #3. Modified from the accompanying manuscript by Weinmann *et al.*

Variant	Source	Library1	Library3
AAV1_wt	(Hoggan, Blacklow, & Rowe, 1966a)	✔	✔
AAV1_A1	Dirk Grimm Laboratory	✔	✔
AAV1_A2	Dirk Grimm Laboratory	✔	✔
AAV1_A6	Dirk Grimm Laboratory	✔	✔
AAV1_P2	Dirk Grimm Laboratory	✔	✖
AAV1_P4	Dirk Grimm Laboratory	✔	✔
AAV1_P5	Dirk Grimm Laboratory	✔	✔
AAV2_wt	(Hoggan, Blacklow, & Rowe, 1966b)	✔	✔
AAV2_A1	Dirk Grimm Laboratory	✔	✔
AAV2_A2	Dirk Grimm Laboratory	✔	✔
AAV2_A6	Dirk Grimm Laboratory	✔	✔
AAV2_P2	Dirk Grimm Laboratory	✔	✔
AAV2_P4	Dirk Grimm Laboratory	✔	✔
AAV2_P5	Dirk Grimm Laboratory	✔	✔
AAV2_7m8	(Dalkara et al., 2013)	✖	✔
AAV2_BR1	(Körbelin, Dogbevia, et al., 2016)	✖	✔
AAV2_L1	(Körbelin, Sieber, et al., 2016)	✖	✔
AAV2_L1mut1	(Körbelin, Sieber, et al., 2016)	✖	✔
AAV2_L1mut2	(Körbelin, Sieber, et al., 2016)	✖	✔
AAV2_MTP	(Yu et al., 2009)	✖	✔
AAV2HBKO	(Opie, Warrington, Agbandje-McKenna, Zolotukhin, & Muzyczka, 2003)	✖	✔
AAV2YF	(Li et al., 2010)	✖	✔
AAV3b_wt	(Rutledge, Halbert, & Russell, 1998)	✔	✔
AAV3b_A1	Dirk Grimm Laboratory	✔	✔
AAV3b_A2	Dirk Grimm Laboratory	✔	✔
AAV3b_A6	Dirk Grimm Laboratory	✔	✔
AAV3b_P2	Dirk Grimm Laboratory	✔	✖
AAV3b_P4	Dirk Grimm Laboratory	✔	✔
AAV3b_P5	Dirk Grimm Laboratory	✔	✔

AAV4_wt	(Parks, Melnick, Rongey, & Mayor, 1967)	✓	✓
AAV4_A1	Dirk Grimm Laboratory	✓	✓
AAV4_A2	Dirk Grimm Laboratory	✓	✓
AAV4_A6	Dirk Grimm Laboratory	✓	✓
AAV4_P2	Dirk Grimm Laboratory	✓	✓
AAV4_P4	Dirk Grimm Laboratory	✓	✓
AAV4_P5	Dirk Grimm Laboratory	✓	✓
AAV4_L1	Dirk Grimm Laboratory	✗	✓
AAV4mut_wt	Dirk Grimm Laboratory	✓	✗
AAV4mut_A1	Dirk Grimm Laboratory	✓	✗
AAV4mut_A2	Dirk Grimm Laboratory	✓	✗
AAV4mut_A6	Dirk Grimm Laboratory	✓	✗
AAV4mut_P2	Dirk Grimm Laboratory	✓	✗
AAV4mut_P4	Dirk Grimm Laboratory	✓	✗
AAV4mut_P5	Dirk Grimm Laboratory	✓	✗
AAV5_wt	(Bantel-Schaal & Zur Hausen, 1984)	✓	✓
AAV5_A1	Dirk Grimm Laboratory	✓	✓
AAV5_A2	Dirk Grimm Laboratory	✓	✓
AAV5_A6	Dirk Grimm Laboratory	✓	✓
AAV5_P2	Dirk Grimm Laboratory	✓	✗
AAV5_P4	Dirk Grimm Laboratory	✓	✓
AAV5_P5	Dirk Grimm Laboratory	✓	✓
AAV6_wt	(Rutledge et al., 1998)	✓	✓
AAV6_A1	Dirk Grimm Laboratory	✓	✗
AAV6_A2	Dirk Grimm Laboratory	✓	✗
AAV6_A6	Dirk Grimm Laboratory	✓	✗
AAV6_P2	Dirk Grimm Laboratory	✓	✗
AAV6_P4	Dirk Grimm Laboratory	✓	✓
AAV6_P5	Dirk Grimm Laboratory	✓	✗
AAV6.2	(Limberis, Vandenberghe, Zhang, Pickles, & Wilson, 2009)	✗	✓

AAV7_wt	(G.-P. Gao et al., 2002)	✓	✓
AAV7_A1	Dirk Grimm Laboratory	✓	✓
AAV7_A2	Dirk Grimm Laboratory	✓	✓
AAV7_A6	Dirk Grimm Laboratory	✓	✓
AAV7_P2	Dirk Grimm Laboratory	✓	✓
AAV7_P4	Dirk Grimm Laboratory	✓	✓
AAV7_P5	Dirk Grimm Laboratory	✓	✓
AAV8_wt	(G.-P. Gao et al., 2002)	✓	✓
AAV8__A1	Dirk Grimm Laboratory	✓	✓
AAV8__A2	Dirk Grimm Laboratory	✓	✓
AAV8_A6	Dirk Grimm Laboratory	✓	✓
AAV8_P2	Dirk Grimm Laboratory	✓	✓
AAV8_P4	Dirk Grimm Laboratory	✓	✓
AAV8_P5	Dirk Grimm Laboratory	✓	✓
AAV9_wt	(G. Gao et al., 2004)	✓	✓
AAV9_A1	Dirk Grimm Laboratory	✓	✓
AAV9_A2	Dirk Grimm Laboratory	✓	✓
AAV9_A6	Dirk Grimm Laboratory	✓	✓
AAV9_P1	(Kunze et al., 2018)	✗	✓
AAV9_P2	Dirk Grimm Laboratory	✓	✗
AAV9_P3	Dirk Grimm Laboratory	✗	✓
AAV9_P4	Dirk Grimm Laboratory	✓	✓
AAV9_P5	Dirk Grimm Laboratory	✓	✓
AAV9_BR1	Dirk Grimm Laboratory	✗	✓
AAV9_K1	(Varadi et al., 2012)	✗	✓
AAV9_K3	(Varadi et al., 2012)	✗	✓
AAV9K449R_PHP.A	(Deverman et al., 2016)	✗	✓
AAV9K449R_PHP.B	(Deverman et al., 2016)	✗	✓
AAV9K449R_PHP.eB	(Chan et al., 2017)	✗	✓
AAV9K449R_PHP.S	(Chan et al., 2017)	✗	✓
AAV9BI	Boehringer Ingelheim	✗	✓

AAV9LD	(Adachi, Enoki, Kawano, Veraz, & Nakai, 2014)	✗	✓
AAV10_wt	(G. Gao et al., 2004)	✓	✓
AAV10_A1	Dirk Grimm Laboratory	✓	✓
AAV10_A2	Dirk Grimm Laboratory	✓	✓
AAV10_A6	Dirk Grimm Laboratory	✓	✓
AAV10_P2	Dirk Grimm Laboratory	✓	✓
AAV10_P4	Dirk Grimm Laboratory	✓	✓
AAV10_P5	Dirk Grimm Laboratory	✓	✓
AAVpo1_wt	(Bello et al., 2009)	✓	✓
AAVpo1_A1	Dirk Grimm Laboratory	✓	✓
AAVpo1_A2	Dirk Grimm Laboratory	✓	✓
AAVpo1_A6	Dirk Grimm Laboratory	✓	✓
AAVpo1_P2	Dirk Grimm Laboratory	✓	✗
AAVpo1_P4	Dirk Grimm Laboratory	✓	✓
AAVpo1_P5	Dirk Grimm Laboratory	✓	✓
AAV12_wt	(Schmidt et al., 2008)	✓	✓
AAV12_A1	Dirk Grimm Laboratory	✓	✗
AAV12_A2	Dirk Grimm Laboratory	✓	✗
AAV12_A6	Dirk Grimm Laboratory	✓	✗
AAV12_P2	Dirk Grimm Laboratory	✓	✗
AAV12_P4	Dirk Grimm Laboratory	✓	✗
AAV12_P5	Dirk Grimm Laboratory	✓	✗
AAVAnc80L65	(Zinn et al., 2015)	✗	✓
AAVB1	(Choudhury et al., 2016)	✗	✓
AAVDJ	(Grimm et al., 2008)	✗	✓
AAVDJYF	Dirk Grimm Laboratory	✗	✓
AAVLK03	(Lisowski et al., 2014)	✗	✓
AAVM41	(Yang et al., 2009)	✗	✓
AAVshH10	(Klimczak, Koerber, Dalkara, Flannery, & Schaffer, 2009)	✗	✓
AAVAH4-N9	Dirk Grimm Laboratory	✗	✓

AAVAH4-N7	Dirk Grimm Laboratory	✗	✓
AAVAH4-N6	Dirk Grimm Laboratory	✗	✓
AAVAH4-N5	Dirk Grimm Laboratory	✗	✓
AAVAH4-N4	Dirk Grimm Laboratory	✗	✓
AAVAH4-N3	Dirk Grimm Laboratory	✗	✓
AAVAH4-N12	Dirk Grimm Laboratory	✗	✓
AAVAH4-N11	Dirk Grimm Laboratory	✗	✓
AAVAH4-N1	Dirk Grimm Laboratory	✗	✓
AAVAH4b-10	Dirk Grimm Laboratory	✗	✓
AAVAH4-7	Dirk Grimm Laboratory	✗	✓
AAVAH4-6	Dirk Grimm Laboratory	✗	✓
AAVAH4-3	Dirk Grimm Laboratory	✗	✓
AAVAH4-1	Dirk Grimm Laboratory	✗	✓
AAVAH3-N9	Dirk Grimm Laboratory	✗	✓
AAVAH3-N8	Dirk Grimm Laboratory	✗	✓
AAVAH3-N6	Dirk Grimm Laboratory	✗	✓
AAVAH3-N5	Dirk Grimm Laboratory	✗	✓
AAVAH3-N4	Dirk Grimm Laboratory	✗	✓
AAVAH3-N2	Dirk Grimm Laboratory	✗	✓
AAVAH3-N12	Dirk Grimm Laboratory	✗	✓
AAVAH3-N10	Dirk Grimm Laboratory	✗	✓
AAVAH3-N1	Dirk Grimm Laboratory	✗	✓
AAVAH3-5	Dirk Grimm Laboratory	✗	✓
AAVAH3-3	Dirk Grimm Laboratory	✗	✓
AAVAH3-21	Dirk Grimm Laboratory	✗	✓
AAVAH3-18	Dirk Grimm Laboratory	✗	✓
AAVAH3-17	Dirk Grimm Laboratory	✗	✓
AAVAH3-10	Dirk Grimm Laboratory	✗	✓
AAVJEA3-S8	Dirk Grimm Laboratory	✗	✓
AAVJEA3-S7	Dirk Grimm Laboratory	✗	✓
AAVJEA3-S5	Dirk Grimm Laboratory	✗	✓
AAVJEA3-S4	Dirk Grimm Laboratory	✗	✓

AAVJEA3-S2	Dirk Grimm Laboratory	✕	✓
AAVJEA3-S10	Dirk Grimm Laboratory	✕	✓
AAVJEA3-S1	Dirk Grimm Laboratory	✕	✓
AAVJEA3-H5	Dirk Grimm Laboratory	✕	✓
AAVJEA3-H4	Dirk Grimm Laboratory	✕	✓
AAVJEA3-H3	Dirk Grimm Laboratory	✕	✓
AAVJEA3-H20	Dirk Grimm Laboratory	✕	✓
AAVJEA3-H19	Dirk Grimm Laboratory	✕	✓
AAVJEA3-H15	Dirk Grimm Laboratory	✕	✓
AAVJEA3-H13	Dirk Grimm Laboratory	✕	✓
AAVJEA3-D7	Dirk Grimm Laboratory	✕	✓
AAVJEA3-D5	Dirk Grimm Laboratory	✕	✓
AAVJEA3-D4	Dirk Grimm Laboratory	✕	✓
AAVJEA3-D20	Dirk Grimm Laboratory	✕	✓
AAVJEA3-D2	Dirk Grimm Laboratory	✕	✓
AAVJEA3-D16	Dirk Grimm Laboratory	✕	✓
AAVJEA3-D12	Dirk Grimm Laboratory	✕	✓
AAVJEA2-S11	Dirk Grimm Laboratory	✕	✓
AAVJEA2-H8	Dirk Grimm Laboratory	✕	✓
AAVJEA2-H2	Dirk Grimm Laboratory	✕	✓
AAVJEA2-H17	Dirk Grimm Laboratory	✕	✓
AAVJEA2-H11	Dirk Grimm Laboratory	✕	✓
AAVJEA2-H1	Dirk Grimm Laboratory	✕	✓
AAVJEA2-D7	Dirk Grimm Laboratory	✕	✓
AAVJEA2-D3	Dirk Grimm Laboratory	✕	✓
AAVJEA2-D16	Dirk Grimm Laboratory	✕	✓
AAVJEA2-D10	Dirk Grimm Laboratory	✕	✓
AAVJEA2-D1	Dirk Grimm Laboratory	✕	✓

Supplementary Table S5_differentially-expressed-genes

geneSymbol	geneID	foldChange	log2FoldChange	meanExpression	adjustedPvalue
**Sparcl1**	ENSMUSG00000029309.7	0.4586443309084443	-1.1245522875247096	569.6949982621883	5.481135346975202e-4
**Nr2f1**	ENSMUSG00000069171.14	0.563400502846002	-0.8277672435403163	81.34569380445812	0.002657252798171417
**A830082K12Rik**	ENSMUSG00000087143.9	0.6435593143024395	-0.6358549728494258	118.01421227043072	0.010666606992532435
**Ntsr2**	ENSMUSG00000020591.11	0.564793019355379	-0.8242058373753183	196.08528815354265	0.011409209023240174
**Fjx1**	ENSMUSG00000075012.4	0.6591223494942214	-0.6013818047299784	160.55578280494055	0.01369812888728773
**Cdh19**	ENSMUSG00000047216.8	0.5153151771265769	-0.9564730113372258	62.700375544987914	0.019105903951354777
**Trps1**	ENSMUSG00000038679.16	0.7828015113716924	-0.35328155347765244	480.71259614096226	0.026182598611121745
**Ncald**	ENSMUSG00000051359.15	0.5974398127275874	-0.7431347144351399	40.6614874099513	0.026182598611121745
**Adgrl3**	ENSMUSG00000037605.16	0.7417745392837614	-0.4309473453525118	453.20421824442883	0.027474332239434
**Pla2g7**	ENSMUSG00000023913.17	0.5039862617870257	-0.9885436871976164	208.61311157145997	0.032032920538555325
**Plpp3**	ENSMUSG00000028517.8	0.718722646538464	-0.4764929496184573	876.0664633949602	0.032032920538555325
**Rora**	ENSMUSG00000032238.17	0.7871722104845883	-0.34524880472817765	682.6794750293336	0.032032920538555325
**Nkain2**	ENSMUSG00000069670.8	0.6475559011785621	-0.6269233540233836	294.28899085669553	0.04750224896887462
**Rmst**	ENSMUSG00000112117.1	0.549166725361834	-0.8646838812919768	106.73728603443496	0.04750224896887462
**Abr**	ENSMUSG00000017631.18	0.7122887560091385	-0.4894658783999692	115.36379717416378	0.04854367969120873
**Zhx2**	ENSMUSG00000071757.10	0.6853963040813458	-0.5449896825719882	94.5402551032734	0.04854367969120873
**Shisa9**	ENSMUSG00000022494.15	0.3075413864236202	-1.7011475251820585	42.884210839461865	0.04854367969120873
**Ptprt**	ENSMUSG00000053141.16	0.5883901763676116	-0.7651549350112068	377.971608777912	0.04854367969120873

Supplementary Table S6_input-library-info

Variant	Barcode	PercentOfLibrary	Library
**AAV12_A1**	TGTTTAGGTGAGCCT	0.003466195	Library #1
**AAV12_A2**	TGTGGTGTGACTCAG	0.0011020722	Library #1
**AAV12_A6**	TCGGGTTGGTCTTTG	0.001116885	Library #1
**AAV12_P2**	AGCCTAATCTTTGAC	0.0144365539	Library #1
**AAV12_P4**	AAGCACTAAAGAACA	0.011716924	Library #1
**AAV12_P5**	GGTATGGCCTGCCGC	0.0035017457	Library #1
**AAV12_WT**	GTAGCTGAGGTTGGT	0.2081672263	Library #1
**AAV1_A1**	TTGCCGTCCTTCGAG	0.2329845897	Library #1
**AAV1_A2**	TTCAGCGGACGGGCC	0.0506064464	Library #1
**AAV1_A6**	GTCAGTCCGCTCTTT	0.0923489138	Library #1
**AAV1_P2**	TAGAGATTTAAACCG	0.008250729	Library #1
**AAV1_P4**	CGTGACAGCGGATGG	0.3035231383	Library #1
**AAV1_P5**	TGGGCGGTCAGGGTC	0.4734644556	Library #1
**AAV1_WT**	AGACTCGTTGTATAT	0.8238582524	Library #1
**AAV2_A1**	GTGCTTCTGGCGGAT	1.0157254748	Library #1
**AAV2_A2**	CGGCTGTCGGTCGCC	0.8408692707	Library #1
**AAV2_A6**	ATCGTACGTTACTGA	0.803570643	Library #1
**AAV2_P2**	TCAACATGGGCAACG	0.4276218054	Library #1
**AAV2_P4**	CTTGATCGACGCCCA	0.7037175652	Library #1
**AAV2_P5**	TACGCTATTCAATCT	0.6199926629	Library #1
**AAV2_WT**	TTAAGATCCTGGTCG	1.9217710609	Library #1
**AAV3_A1**	TAACGTTGGGTTGCC	1.1127611582	Library #1
**AAV3_A2**	GACCACTAGAAGGGC	0.8236893865	Library #1
**AAV3_A6**	CTGCATGGCGGAGTT	1.3856958666	Library #1
**AAV3_P2**	CGTATCGGGTCCGGA	0.1889728014	Library #1
**AAV3_P4**	TGGTTGGGTTTGTGG	1.6613146543	Library #1
**AAV3_P5**	TCGTTGTAACGGTAC	1.8278519904	Library #1
**AAV3_WT**	GATTCGAAAGCATAG	2.2215258232	Library #1
**AAV4_A1**	ACCATAGCGCCACGA	0.2361367533	Library #1
**AAV4_A2**	GTCCCGACTAGGACT	0.0979303765	Library #1
**AAV4_A6**	GTCTTGATTGCTTCG	0.2611377954	Library #1
**AAV4mut_A1**	TGAGAGTCATCCAAG	0.3431651509	Library #1

**AAV4mut_A2**	CCTAATCTCAGGCGG	3.110688e-4	Library #1
**AAV4mut_A6**	CGTGACCCAGGAAGT	0.4054055695	Library #1
**AAV4mut_P2**	AGACTTGGCGTTATG	0.1218767473	Library #1
**AAV4mut_P4**	ACGTGTCGTAGTAAG	0.4234949596	Library #1
**AAV4mut_P5**	TATATTGAGGCGTGT	0.4400645565	Library #1
**AAV4mut_WT**	ACATTGTGGTCATAG	0.4698797583	Library #1
**AAV4_P2**	TGGTAGGTTCGAAAT	0.1096443379	Library #1
**AAV4_P4**	ACGTCGCACCGTTTG	0.507003595	Library #1
**AAV4_P5**	CAGGCTTAACGCGGG	0.3978510421	Library #1
**AAV4_WT**	TCAACGATTGTCTGG	0.3478311826	Library #1
**AAV5_A1**	TTGGACTCACAGATG	4.1719685377	Library #1
**AAV5_A2**	AAGGTGACCTAGTGT	3.8578127723	Library #1
**AAV5_A6**	CCCTCATGAGGTCCG	4.2980550825	Library #1
**AAV5_P2**	GGCCACCGTGTGTGA	0.0616745697	Library #1
**AAV5_P4**	ATGAGCAGCGAATGA	4.6984124501	Library #1
**AAV5_P5**	ATGTTTAACGGCATA	5.3407398513	Library #1
**AAV5_WT**	ATTTGGCACAGGATG	3.9133785438	Library #1
**AAV6_A1**	GTTAACGCGGCCATT	0.0071634696	Library #1
**AAV6_A2**	AGCGGCGTTTATCGT	0.0059191945	Library #1
**AAV6_A6**	TTGGTATGTGTCAAT	0.0210252868	Library #1
**AAV6_P2**	GCGAGGTCGTTAGTT	0.0036913495	Library #1
**AAV6_P4**	TAAGACTGTTCCGGG	0.0389132229	Library #1
**AAV6_P5**	GTTTGTAATCTCTAC	0.0330740175	Library #1
**AAV6_WT**	ATGACAATGTGCAGG	0.1905785088	Library #1
**AAV7_A1**	ACGATCGTACGTCTT	1.3022968459	Library #1
**AAV7_A2**	GTTCAGGTCAGGTCT	0.9521726421	Library #1
**AAV7_A6**	TAAGGAGGGCTGTAG	1.0177489031	Library #1
**AAV7_P2**	GAGCGTAATTGTGAG	0.7489469655	Library #1
**AAV7_P4**	CGTTAACCCGAAAGC	1.2039842993	Library #1
**AAV7_P5**	GTGACATGCAGGTAG	1.2799295195	Library #1
**AAV7_WT**	GTCGACTTCATGGCA	2.2909711872	Library #1
**AAV8_A1**	GGGCCCTAGCGCGTG	0.5194019078	Library #1
**AAV8_A2**	GATAGGCTGGTCCAA	0.4331706799	Library #1
**AAV8_A6**	TATTTGTGTCGTTCC	0.4140769821	Library #1

**AAV8_P2**	GCTCTGGATGTAGTA	0.1294609003	Library #1
**AAV8_P4**	TAGATGTGGCGGACA	0.6946550948	Library #1
**AAV8_P5**	GTCAACATCGTTACA	0.6880722869	Library #1
**AAV8_WT**	TATCAAGCTAACGTT	0.8518870306	Library #1
**AAV9_A1**	GCCGGAGTCCCGGTA	3.1439484398	Library #1
**AAV9_A2**	CGAGTCGTATGTGGC	2.5158502133	Library #1
**AAV9_A6**	AGTAATTGGTCTTGG	3.5015234816	Library #1
**AAV9_P2**	GCGGAACATAGGCGG	0.218802816	Library #1
**AAV9_P4**	GCCCTTCAGTCAGCT	4.4970294857	Library #1
**AAV9_P5**	CGGTCGCGTGACGTG	3.8997418811	Library #1
**AAV9_WT**	AGTTAGGGCGCTGCG	5.411580581	Library #1
**AAVpo1_A1**	TTGGAACGTGGGCTT	1.7915784083	Library #1
**AAVpo1_A2**	AGATTCAAAGCTGCG	0.9802014203	Library #1
**AAVpo1_A6**	TGTTGGAAGGTATCA	1.728911381	Library #1
**AAVpo1_P2**	TGTCCGGAAAGGACA	0.0635291322	Library #1
**AAVpo1_P4**	GTTGTGCCCTGAGTG	2.8682881764	Library #1
**AAVpo1_P5**	ACCGTATCTCTCCGG	2.2795416316	Library #1
**AAVpo1_WT**	TGGTTTACAAATTAT	1.8485276952	Library #1
**AAVrh10_A1**	CTACCTATTTACTCT	0.505166808	Library #1
**AAVrh10_A2**	ACCGGGCGTTGAGGC	0.6713041985	Library #1
**AAVrh10_A6**	ACTGTGATGGGTTAG	0.612764017	Library #1
**AAVrh10_P2**	GACTTGGTTGTGACG	0.0944789943	Library #1
**AAVrh10_P4**	TTGTTGTATGAGCAG	0.6312622404	Library #1
**AAVrh10_P5**	TCCACGGAGGCTGCG	0.6370421945	Library #1
**AAVrh10_WT**	GGTCTTTGCTCGGTG	0.9843875173	Library #1
**AAV12_WT**	GTAGCTGAGGTTGGT	0.8797413038	Library #3
**AAV1_A1**	TTGCCGTCCTTCGAG	1.0446285517	Library #3
**AAV1_A2**	TTCAGCGGACGGGCC	0.3012612638	Library #3
**AAV1_A6**	GTCAGTCCGCTCTTT	0.9191866476	Library #3
**AAV1_P4**	CGTGACAGCGGATGG	1.1194400931	Library #3
**AAV1_P5**	TGGGCGGTCAGGGTC	0.9804635819	Library #3
**AAV1_WT**	AGACTCGTTGTATAT	1.7084198554	Library #3
**AAV2_7m8**	CGTGACCCAGGAAGT	0.7650928206	Library #3
**AAV2_A1**	GTGCTTCTGGCGGAT	0.255705359	Library #3

**AAV2_A2**	CGGCTGTCGGTCGCC	0.2813060806	Library #3
**AAV2_A6**	ATCGTACGTTACTGA	0.3667152439	Library #3
**AAV2_BR1**	ACGTGTCGTAGTAAG	1.0737699722	Library #3
**AAV2HBKO**	CCTAATCTCAGGCGG	1.1479533251	Library #3
**AAV2_L1**	TATATTGAGGCGTGT	0.5147066682	Library #3
**AAV2_L1mut1**	ATGATCAGCGATATC	0.1970553949	Library #3
**AAV2_L1mut2**	GGTGCCGGACAGCTC	0.2362233568	Library #3
**AAV2_MTP**	AGCGGCGTTTATCGT	0.5277517778	Library #3
**AAV2_P2**	TCAACATGGGCAACG	0.6886006709	Library #3
**AAV2_P4**	CTTGATCGACGCCCA	0.4527444373	Library #3
**AAV2_P5**	TACGCTATTCAATCT	0.6625186101	Library #3
**AAV2_WT**	TTAAGATCCTGGTCG	0.3617305272	Library #3
**AAV2YF**	CTTATGTGAAGAGAT	0.6318352723	Library #3
**AAV3_A1**	TAACGTTGGGTTGCC	0.5328343939	Library #3
**AAV3_A2**	GACCACTAGAAGGGC	0.1884483964	Library #3
**AAV3_A6**	CTGCATGGCGGAGTT	0.0984134813	Library #3
**AAV3_P4**	TGGTTGGGTTTGTGG	0.622591927	Library #3
**AAV3_P5**	TCGTTGTAACGGTAC	0.8738754916	Library #3
**AAV3_WT**	GATTCGAAAGCATAG	0.7399326468	Library #3
**AAV4_A1**	ACCATAGCGCCACGA	0.3741556065	Library #3
**AAV4_A2**	GTCCCGACTAGGACT	0.2107042181	Library #3
**AAV4_A6**	GTCTTGATTGCTTCG	0.4466501929	Library #3
**AAV4_L1**	GGTATGGCCTGCCGC	0.456668576	Library #3
**AAV4_P2**	TGGTAGGTTCGAAAT	3.4295503304	Library #3
**AAV4_P4**	ACGTCGCACCGTTTG	0.7385620536	Library #3
**AAV4_P5**	CAGGCTTAACGCGGG	0.2088359691	Library #3
**AAV4_WT**	TCAACGATTGTCTGG	0.269656039	Library #3
**AAV5_A1**	TTGGACTCACAGATG	0.2461519986	Library #3
**AAV5_A2**	AAGGTGACCTAGTGT	0.2486647527	Library #3
**AAV5_A6**	CCCTCATGAGGTCCG	0.2369576031	Library #3
**AAV5_P4**	ATGAGCAGCGAATGA	0.2925726824	Library #3
**AAV5_P5**	ATGTTTAACGGCATA	0.5152532738	Library #3
**AAV5_WT**	ATTTGGCACAGGATG	0.1734371384	Library #3
**AAV6.2**	TGTGGTGTGACTCAG	2.553529208	Library #3

**AAV6_P4**	TAAGACTGTTCCGGG	2.080568522	Library #3
**AAV6shH10**	AGCCTAATCTTTGAC	0.6027835931	Library #3
**AAV6_WT**	ATGACAATGTGCAGG	0.6671688368	Library #3
**AAV7_A1**	ACGATCGTACGTCTT	0.4132746186	Library #3
**AAV7_A2**	GTTCAGGTCAGGTCT	0.3045653723	Library #3
**AAV7_A6**	TAAGGAGGGCTGTAG	1.0100210753	Library #3
**AAV7_P2**	GAGCGTAATTGTGAG	0.9190724315	Library #3
**AAV7_P4**	CGTTAACCCGAAAGC	0.6356859863	Library #3
**AAV7_P5**	GTGACATGCAGGTAG	0.7268141126	Library #3
**AAV7_WT**	GTCGACTTCATGGCA	0.223145614	Library #3
**AAV8_A1**	GGGCCCTAGCGCGTG	0.4964239349	Library #3
**AAV8_A2**	GATAGGCTGGTCCAA	0.3521037422	Library #3
**AAV8_A6**	TATTTGTGTCGTTCC	0.5162812186	Library #3
**AAV8_P2**	GCTCTGGATGTAGTA	0.553678831	Library #3
**AAV8_P4**	TAGATGTGGCGGACA	0.3955303327	Library #3
**AAV8_P5**	GTCAACATCGTTACA	0.8540997908	Library #3
**AAV8_WT**	TATCAAGCTAACGTT	0.3275309654	Library #3
**AAV9_A1**	GCCGGAGTCCCGGTA	0.4303091332	Library #3
**AAV9_A2**	CGAGTCGTATGTGGC	0.3886447339	Library #3
**AAV9_A6**	AGTAATTGGTCTTGG	0.6598345319	Library #3
**AAV9BI**	ACATTGTGGTCATAG	1.4045071465	Library #3
**AAV9_BR1**	AAGCACTAAAGAACA	0.2648263299	Library #3
**AAV9_K1**	GTTTGTAATCTCTAC	0.3277675559	Library #3
**AAV9_K3**	TTGGTATGTGTCAAT	0.3422158917	Library #3
**AAV9K449R_P**	TCGTTAGTAGCGATC	0.1926417587	Library #3
**AAV9K449R_P**	TGAGAGTCATCCAAG	0.3460013394	Library #3
**AAV9K449R_P**	CGTATCGGGTCCGGA	0.5888900211	Library #3
**AAV9K449R_P**	TAGAGATTTAAACCG	0.4132664604	Library #3
**AAV9LD**	GGCCACCGTGTGTGA	0.5675397698	Library #3
**AAV9_P1**	GCGAGGTCGTTAGTT	0.1599433227	Library #3
**AAV9_P3**	GTTAACGCGGCCATT	0.2386137364	Library #3
**AAV9_P4**	GCCCTTCAGTCAGCT	0.6900365304	Library #3
**AAV9_P5**	CGGTCGCGTGACGTG	0.3978717626	Library #3
**AAV9_WT**	AGTTAGGGCGCTGCG	1.0773025128	Library #3

**AAVAH3-10**	TTCCGTGTGTTGTCT	0.3847450701	Library #3
**AAVAH3-17**	GGACTCAGGCCTGGT	0.3819957255	Library #3
**AAVAH3-18**	CAATCCGGCGCGGGT	0.1895171327	Library #3
**AAVAH3-21**	CCCGTATGTCGGGTA	0.4870174237	Library #3
**AAVAH3-3**	AGTTTCACATGACGG	1.3268646777	Library #3
**AAVAH3-5**	TCGGGTTGGTCTTTG	0.5787003139	Library #3
**AAVAH3-N1**	GGTCAGGACCATTGG	1.2869135197	Library #3
**AAVAH3-N10**	TGGGTTTCGGCATCA	0.8057619082	Library #3
**AAVAH3-N12**	TCGCACGCTGATGTG	0.2506064263	Library #3
**AAVAH3-N2**	TAGTTTATCGCAGGG	0.4156649983	Library #3
**AAVAH3-N4**	GTACCTATCCGTTGT	0.5063607351	Library #3
**AAVAH3-N5**	TGGTCGGCGAGTTTG	0.9596354614	Library #3
**AAVAH3-N6**	ATGTCGAACCCAATC	0.7456108183	Library #3
**AAVAH3-N8**	TTTGGTTGGAGTCTT	0.2409470081	Library #3
**AAVAH3-N9**	AGTTCACGACTGCGA	1.0275858789	Library #3
**AAVAH4-1**	GTTCGTTGCGGGATC	0.8892293979	Library #3
**AAVAH4-3**	GAATCCATGACTTTG	0.6495469252	Library #3
**AAVAH4-6**	GTGTAGGTTATCATC	0.3769131094	Library #3
**AAVAH4-7**	TTACGATTTATGCGC	0.9018258014	Library #3
**AAVAH4b-10**	GTTTACGGATCTCGG	0.8244036064	Library #3
**AAVAH4-N1**	TTACCTTCTAAGGGC	0.414539154	Library #3
**AAVAH4-N11**	TCTGTATGGGCCAGC	0.7840445338	Library #3
**AAVAH4-N12**	TGATCTGACCGTGTG	0.4651287252	Library #3
**AAVAH4-N3**	GGTTGGTTAGGCTGT	0.4590915888	Library #3
**AAVAH4-N4**	ACCGGCAATCCTAGC	0.4980311185	Library #3
**AAVAH4-N5**	GTGTGTTACCTAACA	0.6201689142	Library #3
**AAVAH4-N6**	TCATCTAGCATCGGG	0.4211228959	Library #3
**AAVAH4-N7**	GCCACAGGCATCGTG	0.3821996829	Library #3
**AAVAH4-N9**	TATATAGTCGGTTTG	0.7664797303	Library #3
**AAVAnc80L65**	GCGGAACATAGGCGG	0.5427304026	Library #3
**AAVB1**	TGTCCGGAAAGGACA	0.4654387403	Library #3
**AAVDJ**	AGACTTGGCGTTATG	0.6410296679	Library #3
**AAVDJYF**	GTGCTTGTCATGCCG	0.5324835874	Library #3
**AAVJEA2-D1**	GGTTGACAGTGGGCT	0.9752912245	Library #3

**AAVJEA2-D10**	GTGCGCAGGTTAGTG	0.3474290406	Library #3
**AAVJEA2-D16**	TATAACTTAGCTGAT	0.2695744561	Library #3
**AAVJEA2-D3**	CTTCTTCAGGCAACC	0.2289951097	Library #3
**AAVJEA2-D7**	ACCAACCGGTGTGGG	0.2463396393	Library #3
**AAVJEA2-H1**	GCAATTATCATAGTC	1.7229416159	Library #3
**AAVJEA2-H11**	CCACTAGGATCCGGA	0.186873846	Library #3
**AAVJEA2-H17**	ATCTCGAAGCGCGTA	0.1896395071	Library #3
**AAVJEA2-H2**	TTCATCGGCCGCTAA	0.411610327	Library #3
**AAVJEA2-H8**	CGTCCTGTAAGGAGT	1.2539458601	Library #3
**AAVJEA2-S11**	CAAGGCTTTCTGATC	0.2213099982	Library #3
**AAVJEA3-D12**	TTGGCAGAGGATCAC	0.4518062337	Library #3
**AAVJEA3-D16**	TCGGCTCTGTTCTAG	0.5226854782	Library #3
**AAVJEA3-D2**	TACGTATCGCGTGAT	0.3017181282	Library #3
**AAVJEA3-D20**	TTTAGGCGCGGCTTG	0.2806615755	Library #3
**AAVJEA3-D4**	TCGGCGTGGCGGTCG	0.1824438932	Library #3
**AAVJEA3-D5**	GGTACAGGACGCAGG	0.2046833983	Library #3
**AAVJEA3-D7**	CTAGGCAGGACACCG	0.3269435684	Library #3
**AAVJEA3-H13**	TACATTTAACTGAAG	0.5045577524	Library #3
**AAVJEA3-H15**	CTCGCGGCCTGAGGG	1.2482921635	Library #3
**AAVJEA3-H19**	CTAGATAAATGCGGT	0.4140822896	Library #3
**AAVJEA3-H20**	ACCTGAGTTTGGTGG	0.7553844526	Library #3
**AAVJEA3-H3**	TAGAGTATGAGTGGT	0.2878408728	Library #3
**AAVJEA3-H4**	GAGCGGGCAGACGAT	4.6929271766	Library #3
**AAVJEA3-H5**	GACTTTGACATGTCA	0.1530169325	Library #3
**AAVJEA3-S1**	CCGTCGAAGAAGGGA	0.5183860581	Library #3
**AAVJEA3-S10**	CATGCCATGTGTATC	2.3684665029	Library #3
**AAVJEA3-S2**	CGTCCGTCTAATGAA	0.274118625	Library #3
**AAVJEA3-S4**	GGCAGCGGACACGTG	0.6733365058	Library #3
**AAVJEA3-S5**	GACCACTTATCGCCA	0.1938573443	Library #3
**AAVJEA3-S7**	ATCCTCTCCGCTACC	1.5773813627	Library #3
**AAVJEA3-S8**	TAGCACCATTTACGG	0.2471554686	Library #3
**AAVLK03**	TGTTTAGGTGAGCCT	0.9184931928	Library #3
**AAVM41**	GAGGTCCAGAGGAAG	0.5447536591	Library #3
**AAVpo1_A1**	TTGGAACGTGGGCTT	0.5666749908	Library #3

**AAVpo1_A2**	AGATTCAAAGCTGCG	0.4246717531	Library #3
**AAVpo1_A6**	TGTTGGAAGGTATCA	0.3657851985	Library #3
**AAVpo1_P4**	GTTGTGCCCTGAGTG	0.4853286572	Library #3
**AAVpo1_P5**	ACCGTATCTCTCCGG	0.5556449795	Library #3
**AAVpo1_WT**	TGGTTTACAAATTAT	1.2975356165	Library #3
**AAVrh10_A1**	CTACCTATTTACTCT	0.8566614946	Library #3
**AAVrh10_A2**	ACCGGGCGTTGAGGC	0.17443245	Library #3
**AAVrh10_A6**	ACTGTGATGGGTTAG	0.8216624201	Library #3
**AAVrh10_P2**	GACTTGGTTGTGACG	0.4624283304	Library #3
**AAVrh10_P4**	TTGTTGTATGAGCAG	0.3186384267	Library #3
**AAVrh10_P5**	TCCACGGAGGCTGCG	1.1113307505	Library #3
**AAVrh10_WT**	GGTCTTTGCTCGGTG	0.4966686837	Library #3

Supplemental Figures

a

FACS-sorting strategy to isolate qNSCs; aNSCs; TAPs and neuroblasts

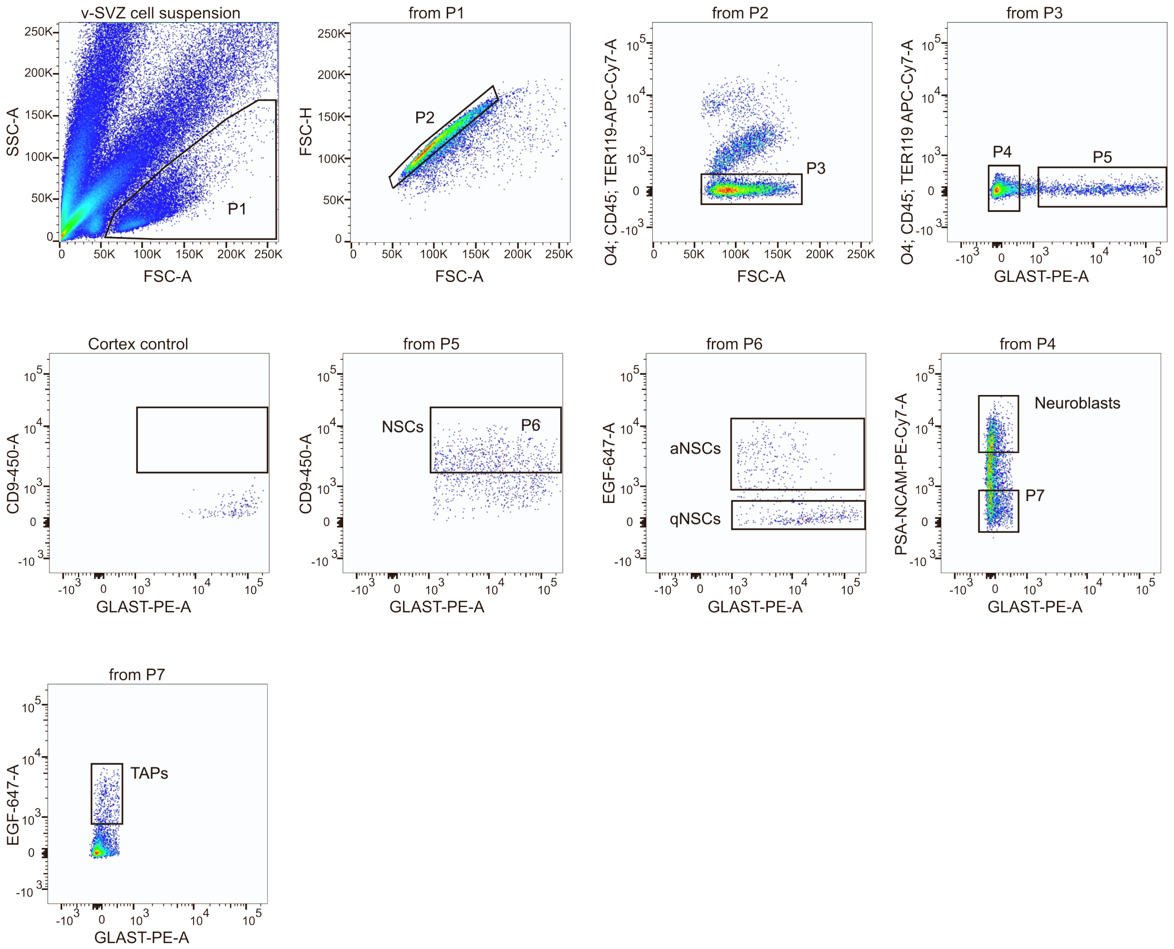

b

FACS-sorting strategy to isolate oligodendrocytes; astrocytes and ependymal cells

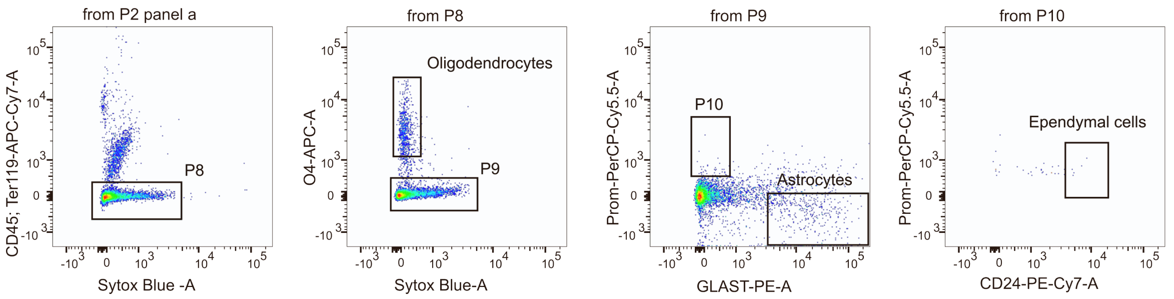

Figure S1, related to Figure 1:

a FACS sorting strategy to isolate total NSCs, qNSCs, aNSCs, TAPs and neuroblasts. **b** FACS sorting strategy to isolate oligodendrocytes, astrocytes and ependymal cells.

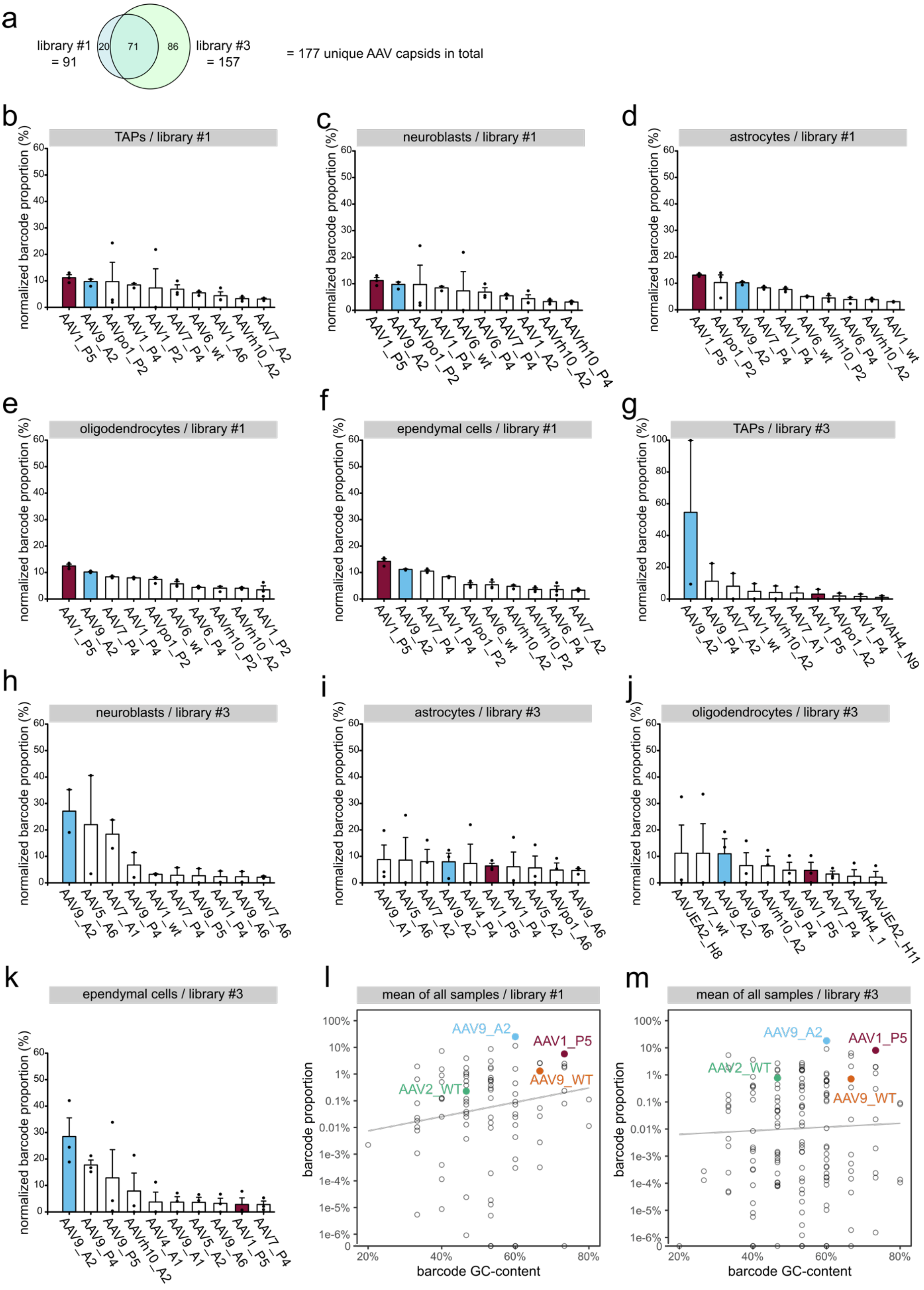

Figure S2, related to Figure 1:

a Number of barcoded AAV capsids in library #1 and library #3. 71 capsids are contained in both libraries. **b-f** Normalized barcode proportion over different FACS-sorted cell types seven days after library #1 transduction of **b** TAPs, **c** neuroblasts, **d** astrocytes, **e** oligodendrocytes and **f** ependymal cells. **g-k** Normalized barcode proportion over different FACS-sorted cell types seven days after library #3 transduction of **g** TAPs; n=2 sets, **h** neuroblasts; n=2 sets, **i** astrocytes, **j** oligodendrocytes and **k** ependymal cells. **l,m** Correlation of barcode GC-content and mean barcode proportion across all samples of library #1 (**l**; Spearman's ρ=0.10; p=0.33) and library #3 (**m**; Spearman's ρ=0.07; p=0.36). All mice were eight weeks old at the time of AAV injection, and all values are given as mean ± SEM; n=3 sets unless stated otherwise.

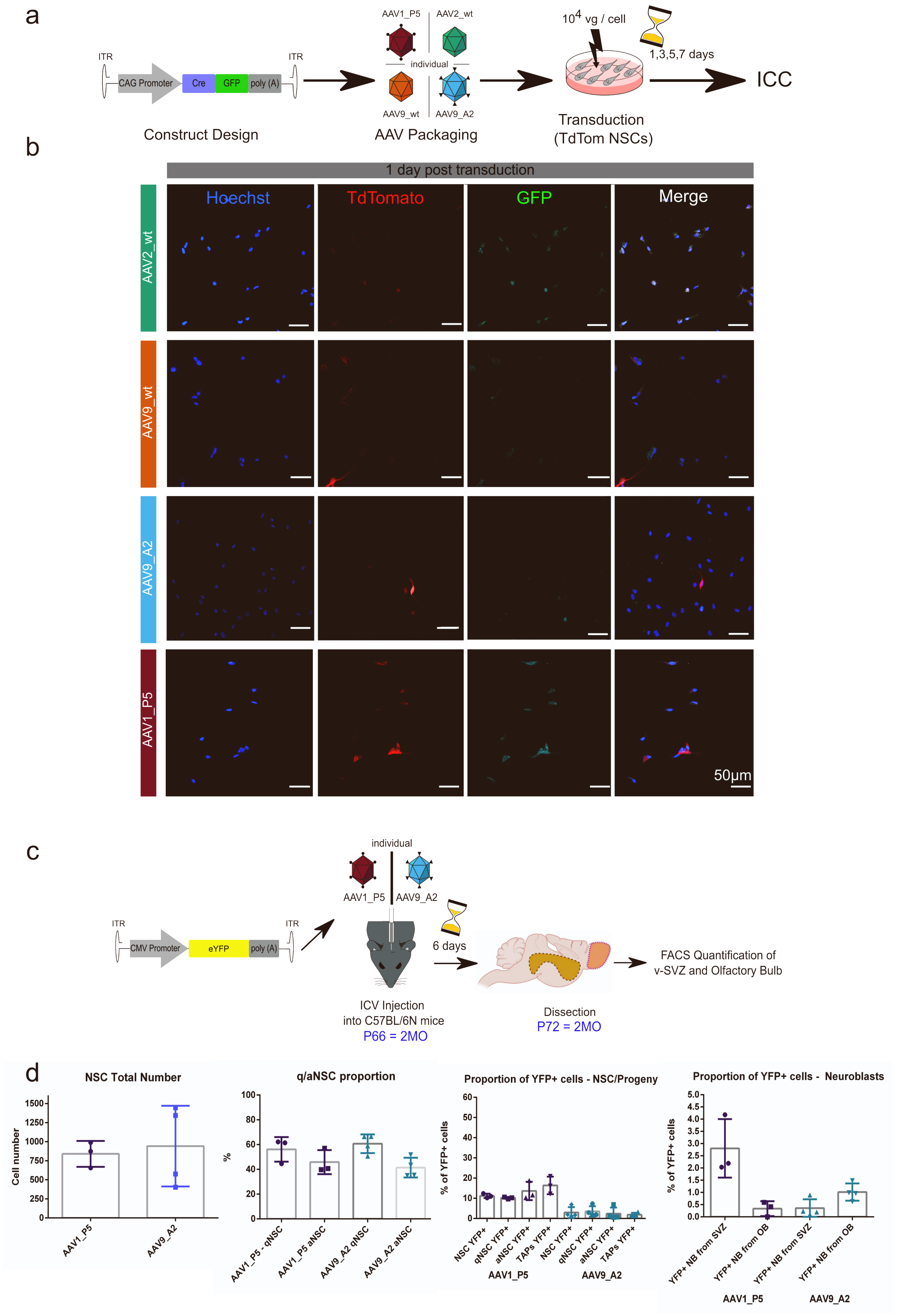

Figure S3, related to Figure 2

a Schematic illustration of the experimental outline to *in vitro* validate different AAV capsids. **b** Representative images of NSCs *in vitro* transduced with different AAV capsids at day 1 after transduction; scale bar 50 µm. **c** Schematic illustration of the experimental outline to perform labeling efficiency analysis of the SVZ and olfactory bulb by FACS Quantification using either AAV1_P5_eYFP or AAV9_A2_eYFP (10^{10}vg/mouse). **d** Quantification of total NSC number in the v-SVZ; proportion of quiescent to active NSCs; labeling efficiency of NSC and TAPs in the v-SVZ; and labeling efficiency of neuroblasts in the v-SVZ and olfactory bulb. The overall NSC labeling efficiency was higher with AAV1_P5 (11.19% ± 0.63, n=3) compared to AAV9_A2 (2.96 ± 1.34, n=4) ($p<0.01$, two-sided Student's t-test). All values are given as mean ± SEM.

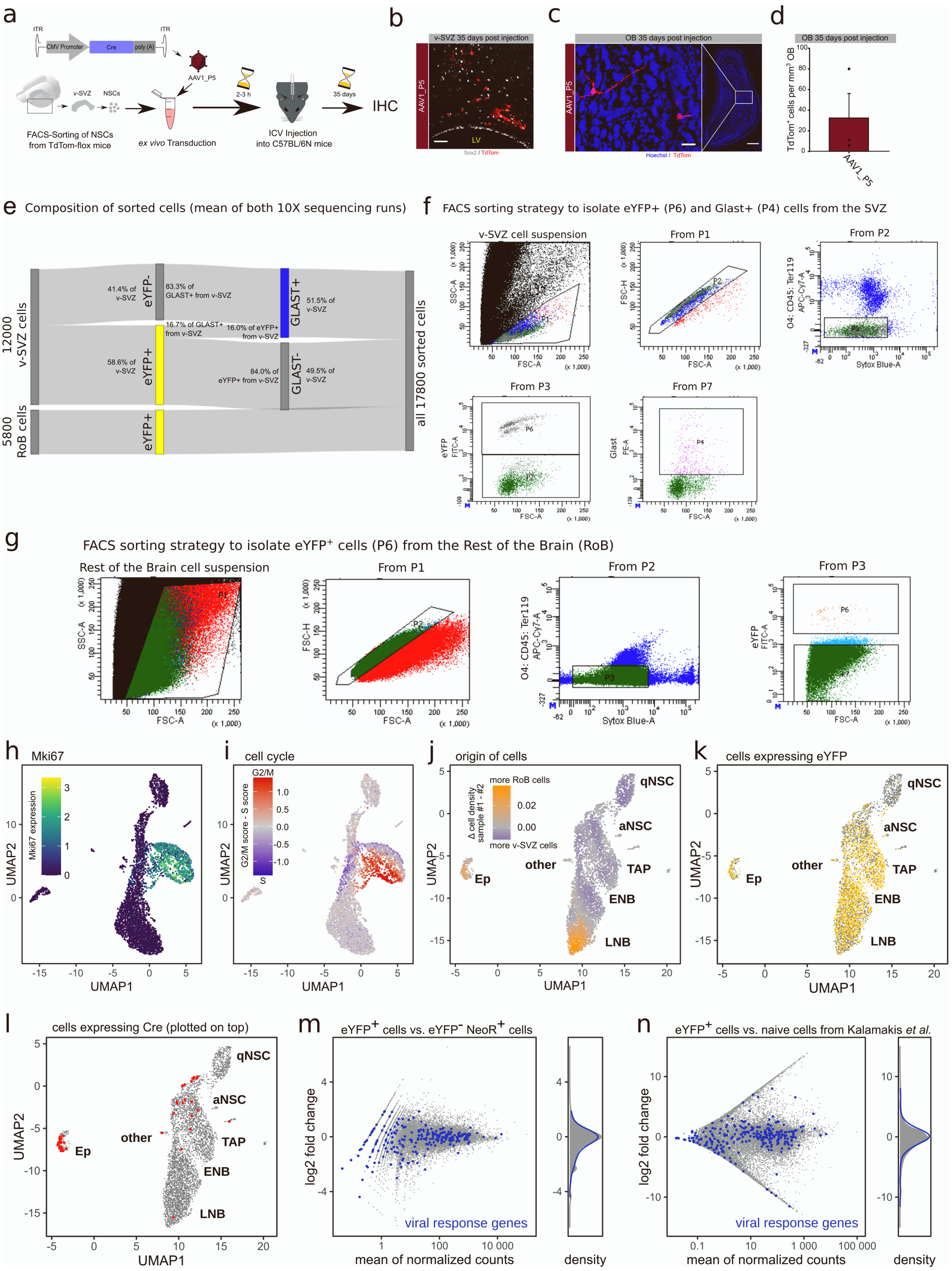

Figure S4, related to Figure 3

a Schematic illustration of the experimental outline to perform *ex vivo* manipulation and transplantation of NSCs. **b** IHC of the v-SVZ (scale bar 50 µm) and **c** Olfactory bulb (OB) neurons (scale bar 200 µm and 30 µm). **d** Quantification of tdTomato-positive cells in the OB; n=3. For this part, all mice were eight weeks old at the time of stereotactic injection of AAVs. **e** Composition of cells that were sorted for scRNA-seq. Cells are grouped according to their tissue of origin (v-SVZ or RoB) and their surface marker (eYFP and/or GLAST) expression. Shown is the average of both single-cell RNA sequencing runs. **f** FACS sorting strategy to isolate $eYFP^+$ and also $eYFP^-/GLAST^+$ cells from the v-SVZ. **g** FACS sorting strategy to isolate $eYFP^+$ cells from the RoB. **h-l** 2D representation of single-cell transcriptomes before regressing out the effects of cell cycle heterogeneity (h,i) and after (j-l). **h** Cells expressing Mki67 (proliferation marker protein Ki-67, log-normalized UMI counts) form a distinct group. **i** Cell cycle phase scores highlight cells expressing canonical markers of S phase (blue) and G2/M phase (red). **j** Putative RoB cells are located at the end of the NSC lineage. Cell color indicates whether nearby cells mostly stem from sample #1 or sample #2 (see Methods for details). Sample #1 contains more cells from RoB, hence orange cells in the main lineage are mostly from RoB. **k,l** Cells with at least one eYFP (**k**) or Cre (**l**) transcript are highlighted in yellow or red. **m,n** MA plots of gene expression differences between $eYFP^+$ cells and $eYFP^-$ $NeoR^+$ cells (**m**) or $eYFP^+$ cells and untransduced cells from[2] (**n**). Right: $\log_2$ fold change distribution for all genes (gray) and viral response genes (blue). RoB, rest of the brain (entails the striatum, rostral migratory stream and olfactory bulb).

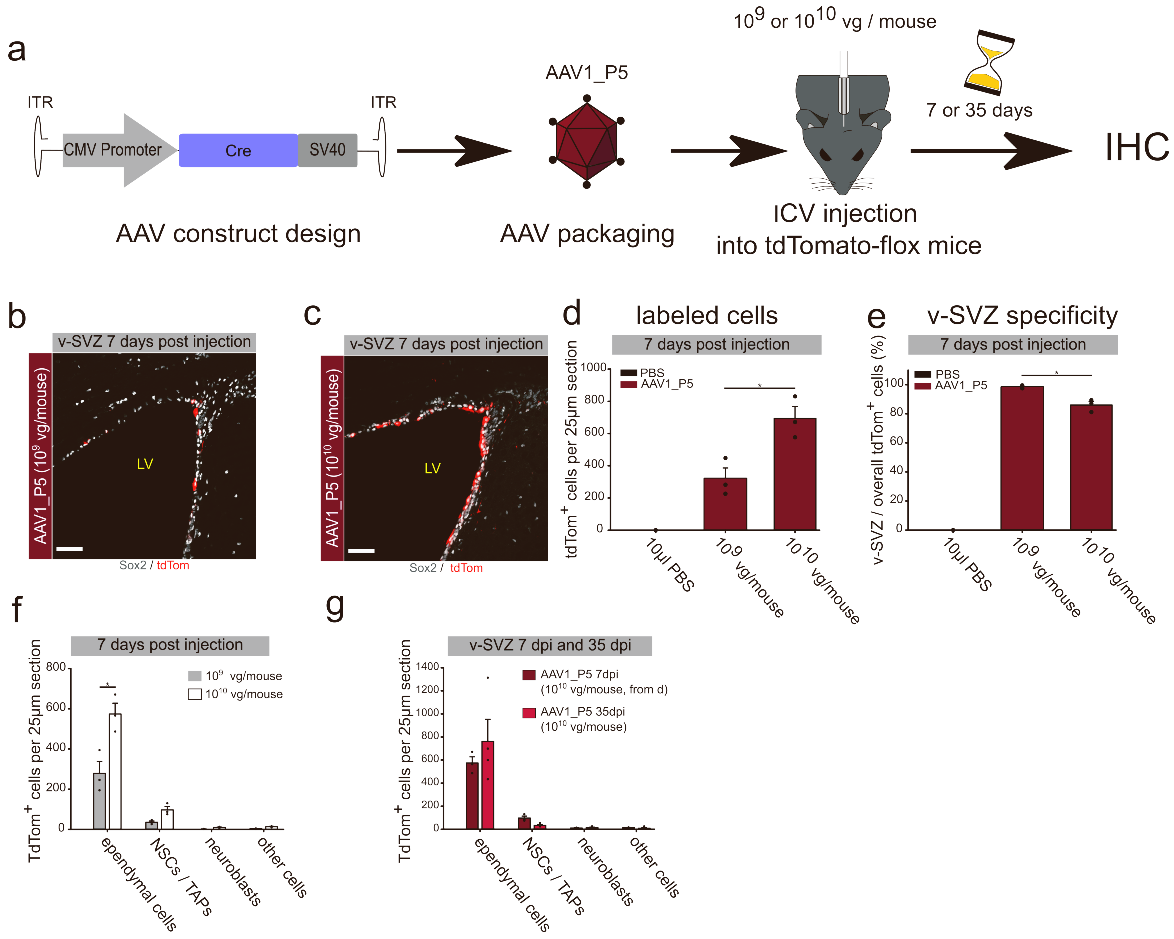

Figure S5 related to Figure 4

a Schematic illustration of the experimental outline to test v-SVZ labeling with different AAV concentrations. **b,c** IHC of the v-SVZ after injecting **b** 10^9 or **c** 10^{10} vg per mouse (scale bar 50 µm). **d** Quantification of the total number of tdTomato-labeled cells within the v-SVZ injected with 10^9 vg per mouse (319.89 ± 66.2) vs. 10^{10} vg per mouse (694 ± 73.92). **e** Quantification of tdTomato-labeled cells located in the v-SVZ among all tdTomato-positive cells in a 25µm thick coronal brain section (10^9 vg/mouse (98.4% ± 0.612) vs. 10^{10} vg/mouse (86.0 % ± 2.51)). **f** Quantification of ependymal cells (10^9 vg per mouse (278.67 ± 59.82) vs. 10^{10} vg per mouse (574.22 ± 53.74)), NSCs, neuroblasts and other cells in the v-SVZ. **g** Quantification of the total number of tdTomato-labeled cells within the v-SVZ of mice at 35dpi; n=4. All mice were eight weeks old at the time of AAV injection; n=3. All values are given as mean ± SEM; $^{**}p \leq 0.01$

and ***$p \leq 0.001$ (Student's t-test). Cre, Cre recombinase; SV40, Simian-Virus 40 polyA signal; ICV, Intracerebroventricular; IHC, immunohistochemistry.

Supplementary Figure S6 related to Figure 4

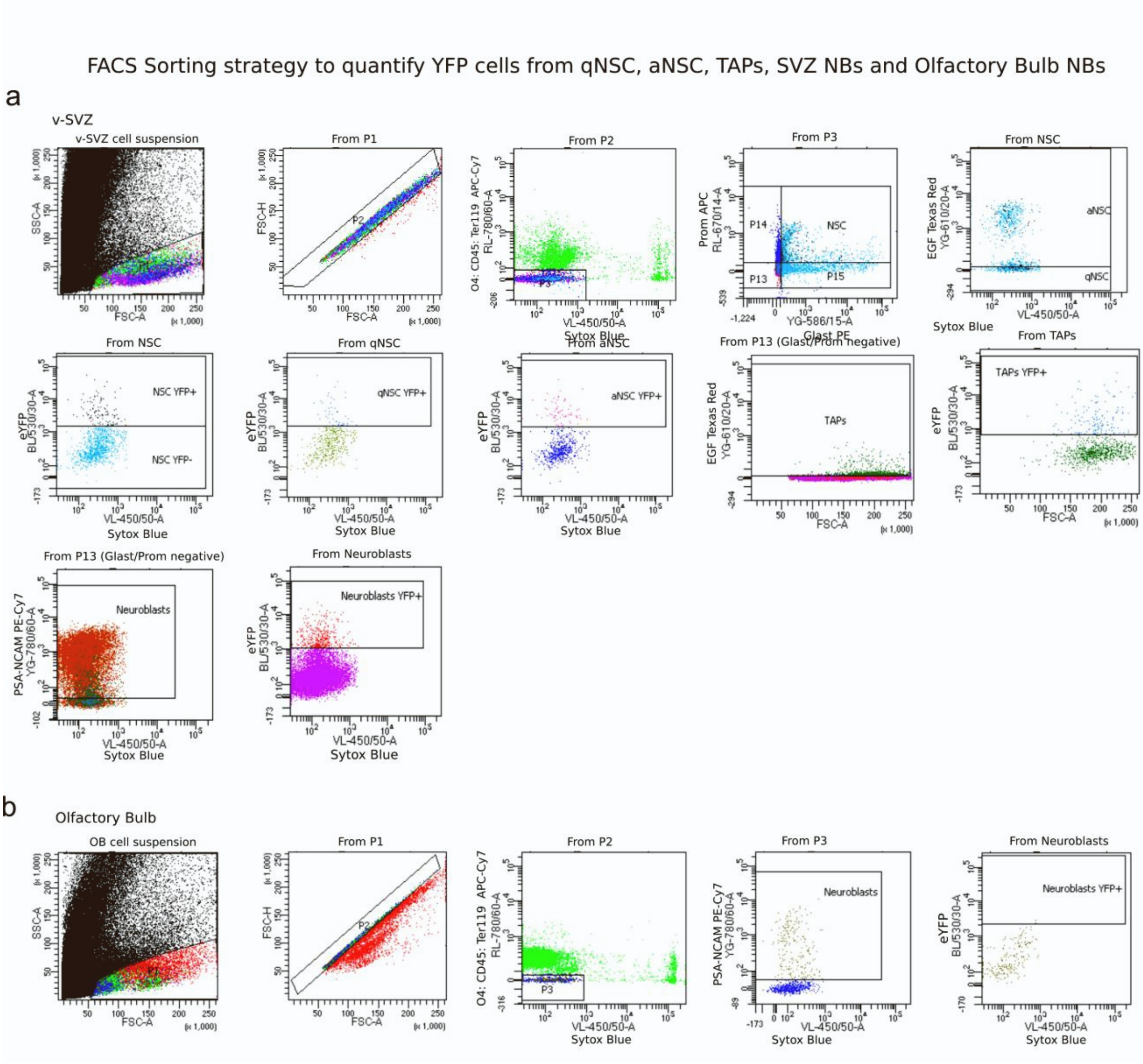

a FACS sorting strategy to quantify the percentage of YFP$^+$ cells from qNSCs, aNSCs, TAPs and NBs in the v-SVZ (related to Fig S3c-d and 4f-g). **b** FACS sorting strategy to quantify YFP$^+$ cells from NBs in the olfactory bulb.
